# α‐Arylation of Carbonyl Compounds through Oxidative C−C Bond Activation

**DOI:** 10.1002/anie.201904899

**Published:** 2019-06-06

**Authors:** Jing Li, Adriano Bauer, Giovanni Di Mauro, Nuno Maulide

**Affiliations:** ^1^ University of Vienna Institute of Organic Chemistry Währinger Strasse 38 1090 Vienna Austria

**Keywords:** arylation, C−C bond activation, enolonium species, ketones, umpolung

## Abstract

A synthetically useful approach for the direct α‐arylation of carbonyl compounds through a novel oxidative C−C bond activation is reported. This mechanistically unusual process relies on a 1,2‐aryl shift and results in all‐carbon quaternary centers. The transformation displays broad functional‐group tolerance and can in principle also be applied as an asymmetric variant.

The appendage of an aryl substituent to the α‐position of a carbonyl moiety remains a transformation of central importance in synthetic organic chemistry. The advent of powerful metal‐catalysed coupling processes has paved the way for the introduction of catalytic (involving mainly organometallic complexes of Pd and Cu) coupling reactions that join aryl halides (or equivalents) to carbonyl‐derived enolates.^1^, ^2^ Prior to and following these advances, useful transition‐metal‐free α‐arylation processes have been developed that involve stoichiometric reactions of enolate anions (or equivalents) with electrophilic aromatic derivatives of Bi^V^,^3^ Pb^IV^,^4^ I(III),^5^ S(IV),^6^ or benzyne.^7^ Stepwise methods via initial formation of *N*‐alkoxyenamines^8^ or enolonium equivalents followed by nucleophilic attack have also been used for the α‐arylation of ketones.9b, 9d

We have established a research program exploiting the electrophilic activation of amides by drawing on pioneering work from the groups of Ghosez,^10^ and more recently Charette,^11^ Movassaghi,^12^ Huang, and others.^13^ A current focus of interest resides in the implications of an umpolung strategy that exploits pyridine *N*‐oxide‐mediated formation of enolonium equivalents^9^, ^14^ under mild conditions, thereby enabling a series of novel transformations for the α‐functionalization of amides.^15^

During these studies, an unexpected result caught our attention (Scheme 1 b). Substrate **A**, which bears a phenyl group in the β‐position of the amide, generated trace amounts of an unexpected product (**C**). Our mechanistic interpretation of this result suggested that fragmentation of the enolonium **B** was triggered by nucleophilic attack of the neighboring arene to generate phenonium intermediate **D**.^16^ Ring opening by weakly nucleophilic triflate accounts for formation of the unexpected product **C**.

**Scheme 1:**
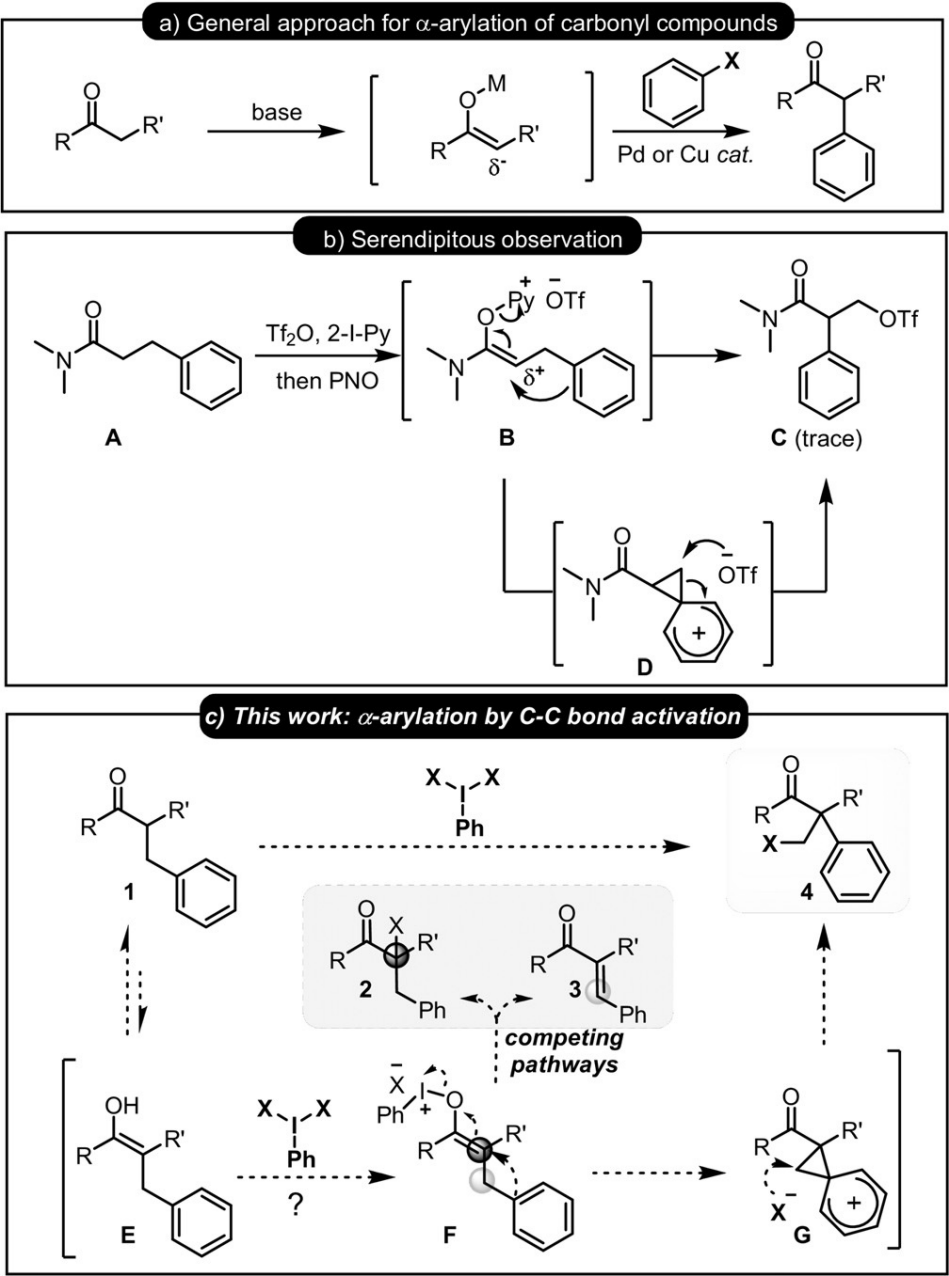
Representative approaches for α‐functionalisation of carbonyl compounds and a proposed arylation through C−C bond activation. PNO=pyridine‐*N*‐oxide, Tf_2_O=trifluoromethanesulfonic anhydride, 2‐I‐Py=2‐iodo‐pyridine.

Aiming to capitalize on this serendipitous observation in a more general context, we hypothesized that a metal‐free α‐arylation that proceeds through skeletal reorganization could be developed (Scheme 1 c). Our mechanistic postulate involved the conversion of a generic α‐disubstituted ketone nucleophile (**1**) to an enolonium (**F**).9a–9c We then hoped to funnel this intermediate selectively to the phenonium intermediate **G**, ring opening of which would effectively constitute a novel approach to the α‐arylation of carbonyl compounds and formation of a quaternary center. Herein we report the development of this approach into a formal metal‐free α‐arylation through oxidative C−C bond activation.

A number of potential pitfalls are readily apparent in this ambitious proposal. Most notably, 1) intermediate **F** has a readily available elimination pathway accessible to generate a particularly stable β‐aryl‐α,β‐unsaturated carbonyl compound (**3**) and 2) even if it survives elimination, intermediate **F** can suffer direct attack by any nucleophile in solution to form (in this case) undesired α‐functionalized umpolung products **2**.9a Bearing these possible problems in mind, we began our investigations on the proposed oxidative C−C bond activation reaction with ketoester **1 a**, a compound that exists to a significant extent in the favorable enol form. Aiming to develop an operationally simple method, we explored the use of commercially available ethyl 2‐benzylacetoacetate **1 a** and different oxidants to mediate the proposed process (see the Supporting Information for a detailed optimization). After considerable experimentation, we found that iodosobenzene (1.2 equiv) and MsOH (2.4 equiv) enable the reorganization of **1 a** to α‐arylation product **4 a** through oxidative C−C bond activation in an excellent 81 % yield (Scheme 2).

**Scheme 2:**
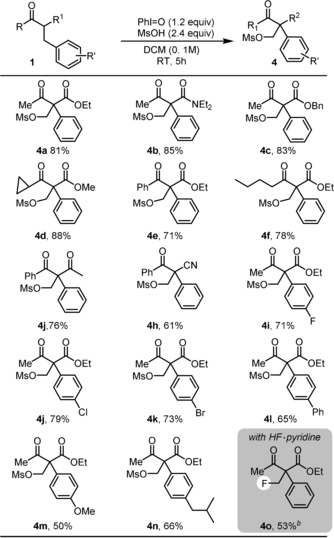
Scope of oxidative C−C bond activation of 2‐benzyl‐substituted 1,3‐dicarbonyl compounds. [a] Reaction Conditions: Reactions conducted on 0.2 mmol scale. All yields refer to pure, isolated materials (see the Supporting Information for details). [b] Pyridine⋅9HF (0.1 mL) used instead of MsOH. DCM=dichloromethane, MSOH=methanesulfonic acid, OMS=mesylate group.

With optimized conditions in hand, we then turned our attention to the scope of this transformation (Scheme 2). First, we evaluated a range of 2‐benzyl‐substituted 1,3‐dicarbonyl compounds under our conditions, including ketoester **4 a**, ketoamide **4 b**, diketone **4 j**, or ketonitrile **4 h**. The reactions proceeded smoothly, affording the desired products in good chemical yield. Furthermore, this transformation exhibited good tolerance to diverse aromatic substitution (**4 i**–**4 n**). Finally, we turned our attention to nucleophiles other than methanesulfonate (MsO^−^). Gratifyingly, when Pyridine⋅9 HF was used instead of MsOH, the β‐fluoride product **4 o** was obtained in moderate yield.

After these promising investigations on oxidative C−C bond activation of active methylene compounds, we turned our attention to simple ketones. Our investigations showed that ketone‐derived silyl enol ethers featuring an arene residue in the allylic position are also amenable to this transformation, resulting in α‐arylated products (Scheme 3). As shown, electron‐donating groups such as 3,4‐di‐OMe are well tolerated in the migrating arene (**6 d**). Their electron‐poor counterparts (e.g., *p*‐CF_3_ in **6 m**)16b afforded lower yields, likely as a consequence of diminished migratory ability. Interestingly, this approach can be employed to convert β,β‐diphenyl‐substituted ketones into product **6 n** in very good yield and as a single diastereoisomer.

**Scheme 3:**
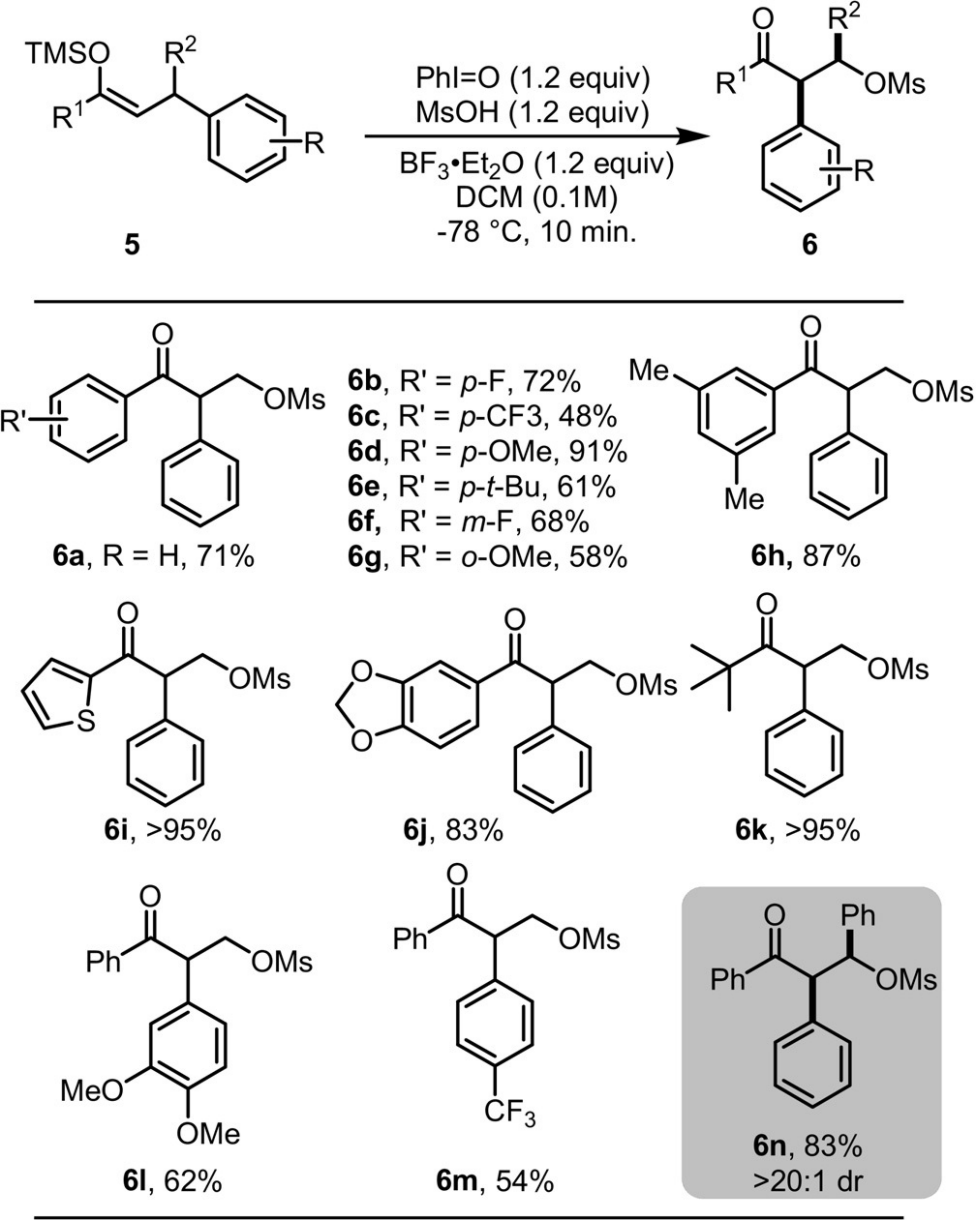
Scope of the oxidative C−C bond activation of ketone‐derived silyl enol ethers. [a] Reactions conducted on 0.2 mmol scale. All yields refer to pure, isolated materials (see the Supporting Information for details).

In a preliminary effort to identify asymmetric variants of this oxidative C−C bond formation, chiral hypervalent iodane **7** 17 was prepared and examined in the reaction of silyl enol ether **5 a** (Scheme 4). Promisingly, the reaction proceeded in good yield and an enantioselectivity value of 70 % *ee* was obtained after only 5 minutes at −78 °C. The resulting α‐arylated ketones lend themselves to further functionalization. For instance, diastereoselective reduction of **6 a** with LiAlH_4_ proceeds in quantitative yield (Scheme 5).^18^ This results in 1,3‐diol **8**, a single isomeric species containing vicinal stereocenters. Alternatively, simple treatment of **6 a** with NH_2_NH_2_ results in pyrazoline **9** 19 in very good chemical yield.

**Scheme 4:**
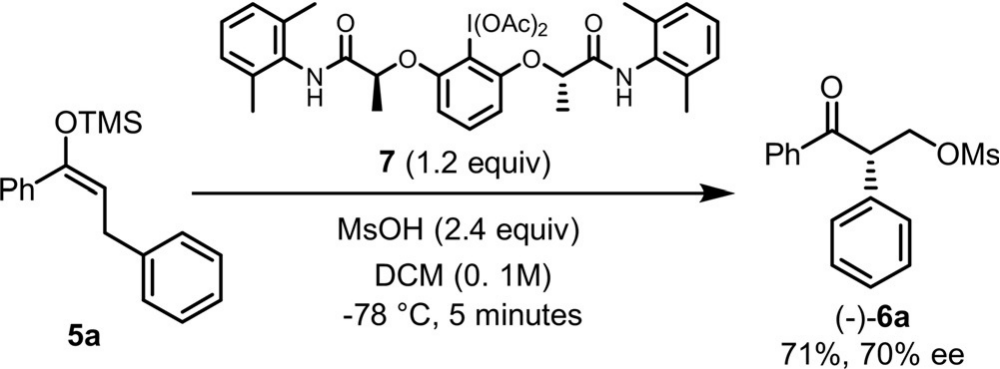
Enantioselective α‐arylation of **5 a** through oxidative C−C bond activation.

**Scheme 5:**
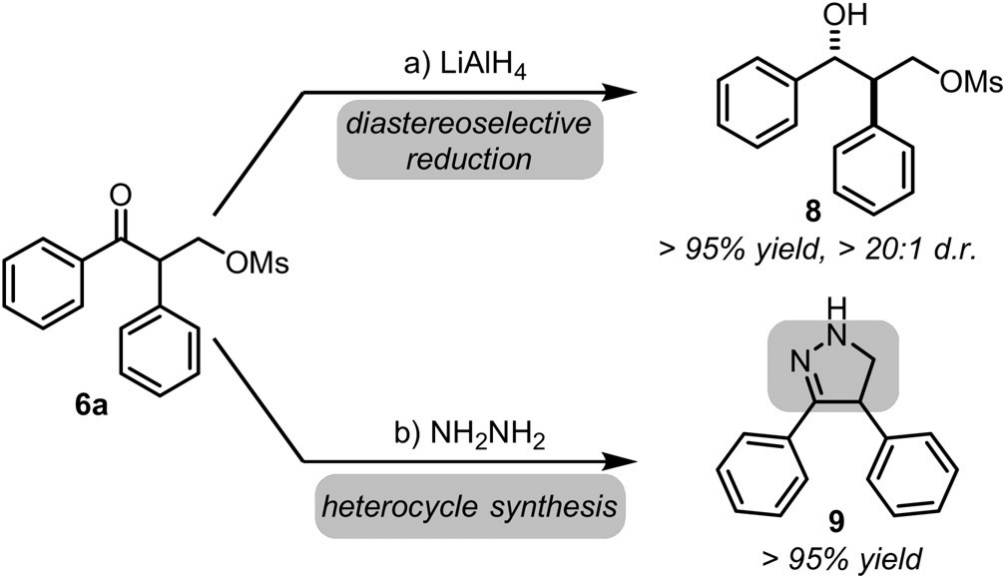
Functionalization of oxidative C−C bond activation product **6 a** (see the Supporting Information for details).

In conclusion, a metal‐free, stereoselective α‐arylation of carbonyl compounds through oxidative C−C bond activation was developed. The ability to use simple and easily available reagents under mild condition is a distinctive feature of this process, which effectively cleaves and reorganize C−C bonds in simple carbonyl‐containing feedstocks.

## Conflict of interest

The authors declare no conflict of interest.

## Supporting information

As a service to our authors and readers, this journal provides supporting information supplied by the authors. Such materials are peer reviewed and may be re‐organized for online delivery, but are not copy‐edited or typeset. Technical support issues arising from supporting information (other than missing files) should be addressed to the authors.

Supplementary
