# Supplementary material for: α‐Arylation of Carbonyl Compounds through Oxidative C−C Bond Activation
Source: Angew Chem Int Ed Engl. 2019 Jun 6;58(29):9816–9. doi: 10.1002/anie.201904899 (PMC6771532; doi:10.1002/anie.201904899)
Supplement: Supplementary file 1 — Supplementary [file ANIE-58-9816-s001.pdf]

Angewandte Chemie
Eine Zeitschrift der Gesellschaft Deutscher Chemiker

Supporting Information

α-Arylation of Carbonyl Compounds through Oxidative C−C Bond Activation

*Jing Li, Adriano Bauer, Giovanni Di Mauro, and Nuno Maulide**

anie_201904899_sm_miscellaneous_information.pdf

Contents

1. General Information 2

2. Optimization of reaction conditions 3

3. Synthesis of 2-benzyl-1,3-dicarbonyl compounds 3

4. Oxidative C-C bond activation 13

5. Enantioselective α-arylation of 5a 28

6. Application 29

7. Spectra 31

1. General Information

Unless otherwise stated, all glassware was flame-dried before use and all reactions were performed under an atmosphere of argon. All solvents were distilled from appropriate drying agents prior to use. All reagents were used as received from commercial suppliers unless otherwise stated. All aldehydes were distilled or purified *via* flash column chromatography before use. Reaction progress was monitored by thin layer chromatography (TLC) performed on aluminum plates coated with silica gel F_{254} with 0.2 mm thickness. Chromatograms were visualized by fluorescence quenching with UV light at 254 nm or by staining using potassium permanganate. Flash column chromatography was performed using silica gel 60 (230-400 mesh, Merck and co.). Neat infra-red spectra were recorded using a Perkin-Elmer Spectrum 100 FT-IR spectrometer. Wavenumbers ($\mathbf{v_{max}}$) are reported in cm^{-1}. Mass spectra were obtained using a Finnigan MAT 8200 or (70 eV) or an Agilent 5973 (70 eV) spectrometer, using electrospray ionization (ESI). All ^{1}H NMR and ^{13}C NMR spectra were recorded using a Bruker AV-400 or AV-600 spectrometer at 300K. Chemical shifts were given in parts per million (ppm, δ), referenced to the solvent peak of $CDCl_3$, defined at δ = 7.26 ppm (^{1}H NMR) and δ = 77.16 (^{13}C NMR). Coupling constants are quoted in Hz (*J*). ^{1}H NMR splitting patterns were designated as singlet (s), doublet (d), triplet (t), quartet (q), pentet (p). Splitting patterns that could not be interpreted or easily visualized were designated as multiplet (m) or broad (br).

2. Optimization of reaction conditions

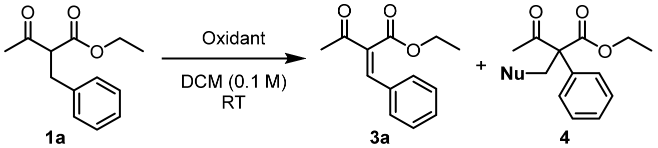

1a 3a 4

Entry	Oxidant	Addtive	Time	Yield (%)	
			(h)	3	4
1	**H**	Ts_2O (1 equiv)	48	<5	<5
2	**I**	Ts_2O (1 equiv)	48	<5	<5
3	**J**	Ts_2O (1 equiv)	48	<5	<5
4	**K**	Ts_2O (1 equiv)	24	80	10
5	**K**	TsOH (2 equiv)	24	81	11
6	**K**	CF_3COOH (2 equiv)	24	70	20
7	**K**	TfOH (2 equiv)	5	trace	trace
8	**K**	MsOH (2 equiv)	5	12	81

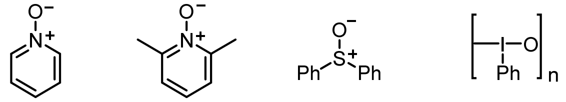

H **I** **J** **K**

[a] Reactions conducted on 0.2 mmol scale. All yields refer to pure, isolated materials. DCM: dichloromethane; Ts_2O: *p*-toluenesulfonic anhydride; *p*-toluenesulfonic acid: TfOH: trifluoromethanesulfonic acid; MSOH: methanesulfonic acid.

3. Synthesis of 2-benzyl-1,3-dicarbonyl compounds

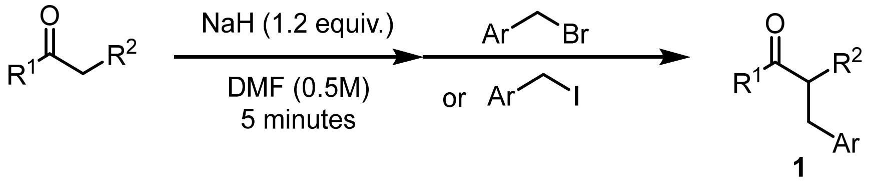

1

General Procedure A: Sodium hydride (12 mmol) added to a flame-dried round bottom flask with dry THF (20 mL) at 0 °C. Then ketone slowly added to the flask until no more gas released, then ice-bath was removed and stirred for further 10 minutes. Benzyl bromide or Benzyl iodide was added in one-portion, which further stirred at room temperature for 1 h. The mixture was poured into 1.0 *N* HCl (100 mL), and extracted with DCM (3 x 50 mL). The combined organic layers were dried with $MgSO_4$, concentrated and purified via column chromatography (silica gel, 20-25% EtOAc in heptanes) to obtain desired product.

Compound **1a** is commercially available.

<u>2-Benzyl-*N,N*-diethyl-3-oxobutanamide</u> (**1b**)

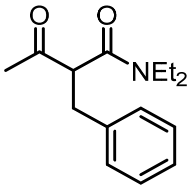

Following the **Procedure A**, compound **1b** obtained as sticky oil in 50% yield. Spectral data was found to be in accordance with the literature: A. T. Babayan, G. O. Torosyan, G. G. Gekchyan, R. T. Grigoryan, G. T. PiskoGudz, O. V. *Fiziol. Aktiv. Veshchestva* **1983** 15, 66

^{1}H NMR (400 MHz, $CDCl_3$) δ 7.38 – 7.17 (m, 5H), 3.79 (dd, *J* = 9.4, 5.3 Hz, 1H), 3.48 – 3.22 (m, 4H), 3.22 – 3.11 (m, 1H), 3.08 – 2.96 (m, 1H), 2.24 (s, 3H), 1.07 (t, *J* = 7.1 Hz, 3H), 0.94 (t, *J* = 7.2 Hz, 3H).

<u>Benzyl 2-benzyl-3-oxobutanoate</u> (**1c**)

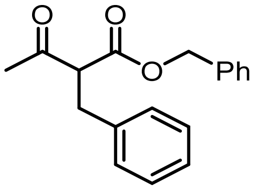

Following the **Procedure A**, compound **1c** obtained in 47% yield as sticky oil. Spectral data was found to be in accordance with the literature: A. M. R. Smith, H. S. Rzepa, A. J. P. White, D. Billen, K. K. M. Hii, *J. Org. Chem.* **2010**, *75*, 3085.

^{1}H NMR (400 MHz, $CDCl_3$) δ 7.41 – 7.33 (m, 4H), 7.32 – 7.22 (m, 4H), 7.17 (dt, *J* = 8.9, 3.2 Hz, 2H), 5.15 (s, 2H), 3.85 (t, *J* = 7.6 Hz, 1H), 3.19 (dd, *J* = 8.1, 6.4 Hz, 2H), 2.17 (s, 3H).

<u>Methyl 2-benzyl-3-cyclopropyl-3-oxopropanoate</u> (**1d**)

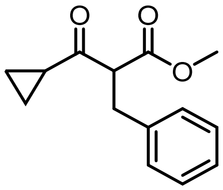

Following the **Procedure A**, compound **1d** obtained in 46% yield as sticky oil. Spectral data was found to be in accordance with the literature: S. M. Yu, K. Cui. F. Lv, Z. Y. Yang, Z. J. Yao, *Tetrahedron Lett.* **2016**, *57*, 2818.

^{1}H NMR (400 MHz, $CDCl_3$) δ 7.35 – 7.27 (m, 3H), 7.26 – 7.18 (m, 2H), 3.99 – 3.90 (m, 1H), 3.72 (s, 3H), 3.23 (d, *J* = 7.6 Hz, 2H), 2.05 (tt, *J* = 7.8, 4.5 Hz, 1H), 1.12 – 0.99 (m, 2H), 0.99 – 0.80 (m, 2H).

Ethyl 2-benzyl-4-methyl-3-oxopentanoate (**1e**)

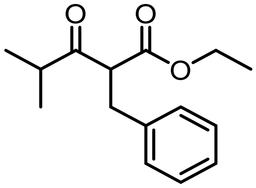

Following the **Procedure A**, compound **1e** obtained in 44% yield as sticky oil.

Spectral data was found to be in accordance with the literature: H. Kaku, T. Imai, R. Kondo, S. Mamba, Y. Watanabe, M. Inai, T. Nishii, M. Horikawa, T. Tsunoda, *Eur. J. Org. Chem.* **2013**, 8208.

^{1}H NMR (400 MHz, $CDCl_3$) δ 7.14 – 7.02 (m, 5H), 4.23 – 4.06 (m, 2H), 3.86 – 3.71 (m, 1H), 3.13 (dd, *J* = 15.3, 5.7 Hz, 2H), 2.45 (m, 1H), 1.26 – 1.14 (m, 3H), 0.90 (d, *J* = 6.6 Hz, 5H).

Ethyl 2-benzyl-3-oxoheptanoate (**1f**)

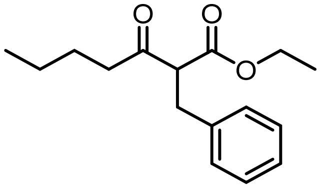

Following the **Procedure A**, compound **1f** obtained in 51% yield as sticky oil.

^{1}H NMR (400 MHz, $CDCl_3$) δ 7.41 – 7.08 (m, 5H), 4.28 – 4.04 (m, 2H), 3.80 (t, *J* = 7.6 Hz, 1H), 3.28 – 3.07 (m, 2H), 2.57 (ddd, *J* = 22.5, 17.3, 7.8 Hz, 1H), 2.36 (dt, *J* = 17.3, 7.2 Hz, 1H), 1.62 – 1.43 (m, 2H), 1.32 – 1.12 (m, 5H), 0.89 (dt, *J* = 14.7, 7.3 Hz, 3H).

^{13}C NMR (101 MHz, $CDCl_3$) δ = 204.8, 169.2, 138.3, 128.8, 128.5, 126.6, 61.4, 60.6, 42.51, 34.1, 25.4, 22.1, 14.0, 13.8.

HRMS (ESI): $[M+H]^+$ calculated for $C_{16}H_{22}O_3Na$ 285.1461; found: 285.1473.

FT–IR (neat): 1738, 1714, 1487, 1358, 1220, 1145, 1009, 851, 827, 769, 697 cm^{-1}.

2-Benzyl-1-phenylbutane-1,3-dione (**1j**)

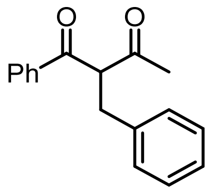

Following the **Procedure A**, compound **1j** obtained in 39% yield as sticky oil.

Spectral data was found to be in accordance with the literature: M. Rueping, B. J. Nachtsheim, A. Kuenkel, *Org. Lett.* 2007, 9, 825–828.

^{1}H NMR (400 MHz, $CDCl_3$) δ 7.94 (d, *J* = 7.4 Hz, 2H), 7.64 – 7.53 (m, 1H), 7.47 (t, *J* = 7.1 Hz, 2H), 7.26 (ddd, *J* = 19.2, 17.5, 15.7 Hz, 5H), 4.82 (t, *J* = 7.0 Hz, 1H), 3.42 – 3.24 (m, 2H), 2.14 (s, 3H).

2-Benzyl-3-oxo-3-phenylpropanenitrile (**1h**)

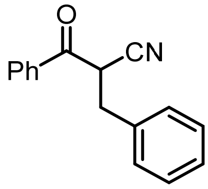

Following the **Procedure A**, compound **1h** obtained in 33% yield as sticky oil. Spectral data was found to be in accordance with the literature: Y. H. Ji, W. C. Trenkle, J. V. Vowles, *Org. Lett.* **2006**, *8*, 1161.

^{1}H NMR (400 MHz, $CDCl_3$) δ 7.99 (dt, *J* = 8.5, 1.6 Hz, 2H), 7.71 – 7.64 (m, 1H), 7.59 – 7.51 (m, 2H), 7.41 – 7.29 (m, 5H), 4.54 (dd, *J* = 8.8, 5.8 Hz, 1H), 3.39 (dd, *J* = 14.0, 5.8 Hz, 1H), 3.33 – 3.22 (m, 1H).

Ethyl 2-(4-fluorobenzyl)-3-oxobutanoate (**1i**)

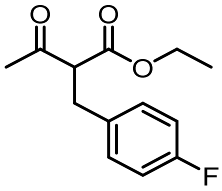

Following the **Procedure A**, compound **1i** obtained in 66% yield as sticky oil.

Spectral data was found to be in accordance with the literature: M. Pieroni, S. K. Tipparaju, S. C. Lun, Y. Song, A. W. Sturm, W. R. Bishai, A. P. Kozikowski, *ChemMedChem* **2011**, *6*, 334.

^{1}**H NMR** (400 MHz, $CDCl_3$) δ 7.23 – 7.06 (m, 2H), 7.04 – 6.92 (m, 2H), 4.23 – 4.10 (m, 2H), 3.75 (t, *J* = 7.6 Hz, 1H), 3.22 – 3.09 (m, 2H), 2.22 (s, 3H), 1.23 (t, *J* = 7.2 Hz, 3H).

Ethyl 2-(4-chlorobenzyl)-3-oxobutanoate (**1j**)

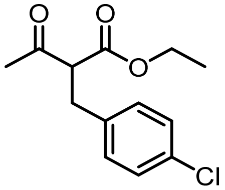

Following the **Procedure A**, compound **1j** obtained in 59% yield as sticky oil.

Spectral data was found to be in accordance with the literature: M. Pieroni, S. K. Tipparaju, S. C. Lun, Y. Song, A. W. Sturm, W. R. Bishai, A. P. Kozikowski, *ChemMedChem* **2011**, *6*, 334.

^{1}**H NMR** (400 MHz, $CDCl_3$) δ 7.34 – 7.20 (m, 2H), 7.20 – 7.11 (m, 2H), 4.25 – 4.10 (m, 2H), 3.75 (t, *J* = 7.6 Hz, 1H), 3.22 – 3.08 (m, 2H), 2.22 (s, 3H), 1.23 (td, *J* = 7.1, 3H)

Ethyl 2-(4-bromobenzyl)-3-oxobutanoate (**1k**)

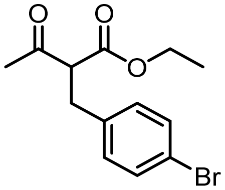

Following the **Procedure A**, compound **1k** obtained in 71% yield as sticky oil.

^{1}**H NMR** (400 MHz, $CDCl_3$) δ 7.48 – 7.35 (m, 2H), 7.11 – 7.05 (m, 2H), 4.26 – 4.09 (m, 2H), 3.80 – 3.68 (m, 1H), 3.20 – 3.07 (m, 2H), 2.22 (s, 3H), 1.28 – 1.19 (m, 3H).

^{13}**C NMR** (101 MHz, $CDCl_3$) δ = 201.9, 168.8, 137.2, 131.7, 130.59, 120.59, 61.61, 61.10, 33.25, 29.57, 14.02.

HRMS (ESI): $[M+Na]^+$ calculated for $C_{13}H_{15}BrO_3Na$ 321.0097, 323.0076; found: 321.0010, 323.0080.

FT–IR (neat): 1737, 1714, 1488, 1358, 1246, 1213, 1145, 1071, 1011, 812, 754 cm^{-1}.

Ethyl 2-([1,1'-biphenyl]-4-ylmethyl)-3-oxobutanoate (**1l**)

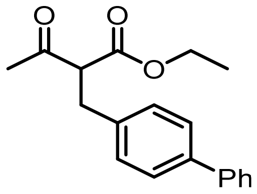

Following the **Procedure A**, compound **1a** obtained in 43% yield as sticky oil.

^{1}H NMR (400 MHz, $CDCl_3$) δ 7.52 – 7.46 (m, 2H), 7.46 – 7.40 (m, 2H), 7.39 – 7.30 (m, 2H), 7.29 – 7.22 (m, 1H), 7.21 – 7.14 (m, 2H), 4.15 – 4.03 (m, 2H), 3.74 (t, *J* = 7.6 Hz, 1H), 3.12 (dd, *J* = 8.1, 6.3 Hz, 2H), 2.15 (s, 3H), 1.18 – 1.09 (m, 3H).

^{13}C NMR (101 MHz, $CDCl_3$) δ = 202.4, 169.1, 140.8, 139.6, 137.3, 129.2, 128.8, 127.3, 127.2, 127.0, 61.5, 61.3, 33.6, 29.6, 14.0.

HRMS (ESI): $[M+Na]^+$ calculated for $C_{19}H_{20}O_3Na$ 319.1305; found: 319.1312.

FT–IR (neat): 2959, 1739, 1713, 1454, 1368, 1220, 1152, 1097, 1032, 776, 699 cm^{-1}.

Ethyl 2-(4-methoxybenzyl)-3-oxobutanoate (**1m**)

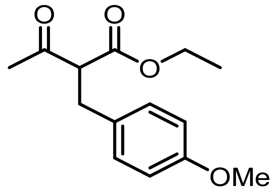

Following the **Procedure A**, compound **1a** obtained in 82% yield as sticky oil.

Spectral data was found to be in accordance with the literature: M. Pieroni, S. K. Tipparaju, S. C. Lun, Y. Song, A. W. Sturm, W. R. Bishai, A. P. Kozikowski, *ChemMedChem* **2011**, *6*, 334.

^{1}H NMR (400 MHz, $CDCl_3$) δ 7.18 – 7.03 (m, 2H), 6.90 – 6.75 (m, 2H), 4.23 – 4.06 (m, 2H), 3.80 (s, 3H), 3.75 (t, *J* = 7.6 Hz, 1H), 3.19 – 3.05 (m, 2H), 2.20 (s, 3H), 1.23 (t, *J* = 7.2 Hz, 3H).

Ethyl 2-(4-isobutylbenzyl)-3-oxobutanoate (**1n**)

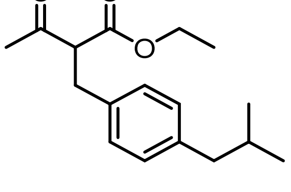

Following the **Procedure A**, compound **1a** obtained in 55% yield as sticky oil.

^{1}H NMR (400 MHz, $CDCl_3$) δ 7.14 – 6.95 (m, 4H), 4.22 – 4.09 (m, 2H), 3.84 – 3.69 (m, 1H), 3.19 – 3.08 (m, 2H), 2.45 (d, *J* = 7.2 Hz, 2H), 2.20 (s, 3H), 1.92 – 1.76 (m, 1H), 1.26 – 1.17 (m, 3H), 0.90 (d, *J* = 6.6 Hz, 6H).

^{13}C NMR (101 MHz, $CDCl_3$) δ = 202.6, 169.2, 140.1, 135.3, 129.3, 128.5, 61.4, 61.4, 45.0, 33.7, 30.2, 29.6, 22.3, 14.0.

HRMS (ESI): $[M+Na]^+$ calculated for $C_{17}H_{24}O_3Na$ 299. 1618; found: 299.1622.

FT–IR (neat): 1737, 1715, 1444, 1366, 1220, 1145, 1116, 1021, 853, 773 cm^{-1}.

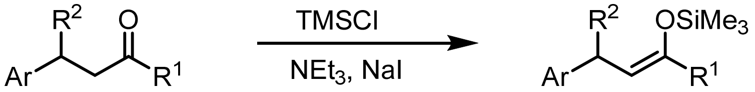

The enol silyl ethers were prepared according to reported procedure. (T. Murafuji, M. Tomura, K. Ishiguro, I. Miyakawa, *Molecules* **2014**, *19*, 11077–11095).

The *E* and *Z* assigned based previous report. (cf. H. J. Reich, R. C. Holtan, C. Bolm. *J. Am. Chem. Soc.* **1990**, *112*, 5609.)

Enol silyl ethers **5a**, **5d**, **5g**, **5h**, **5k, 5l** were reported compounds [cf. a) S. H. Pine, *Org. Reaction*, https://doi.org/10.1002/0471264180.or043.01; b) S. Chiba *et al. Angew. Chem. Int. Ed.* 2018, 57, 6181].

General procedure B: A flame-dried round bottom flask were charged with ketone (1.12 mL, 10 mmol 1 equiv.), CH_3CN (20 mL) and NaI (16 mmol, 1.6 equiv.), followed by the addition of TMSCl (15 mmol, 1.5 equiv.). The reaction mixture was further stirred for 10 minutes, then NEt_3 (20 mmol, 2 equiv.) was slowly added and the reaction was further stirred for 12 h at room temperature. The reaction mixture poured into *sat.* $NaHCO_3$ (100 mL) solution, and extracted with tthyl acetate (3 x 50 mL). The combined organic layers were washed with water (100 mL), dried with $MgSO_4$, concentrated and purified via column chromatography (silica gel, 50% Toluene in heptanes) to afford desired silica eonate.

(*Z*)-((1-(4-Fluorophenyl)-3-phenylprop-1-en-1-yl)oxy)trimethylsilane (5b)

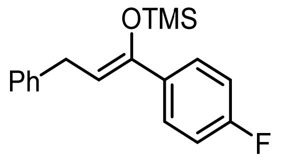

Following the **Procedure B**, compound **5b** was obtained in >95% yield as sticky oil.

^{1}H NMR (400 MHz, $CDCl_3$) δ 7.37 – 7.24 (m, 2H), 7.18 – 6.95 (m, 5H), 6.93 – 6.71 (m, 2H), 5.19 (t, *J* = 7.2 Hz, 1H), 3.40 (d, *J* = 7.2 Hz, 2H), -0.08 (m, 9H).

^{13}C NMR (101 MHz, $CDCl_3$) δ 163.0, 160.6, 148.3, 140.7, 134.6, 127.8, 127.8, 126.6, 126.6, 125.3, 114.4, 114.2, 109.0 (d, *J* = 1.0 Hz), 31.7, -0.0.

HRMS (ESI): $[M+H]^+$ calculated for $C_{18}H_{22}FOSi$ 301.1418; found: 301.1421.

FT–IR (neat): 1647, 1604, 1506, 1453, 1342, 1279, 1252, 1220, 1157, 1101, 1072, 1037, 837, 775, 697 cm^{-1}.

<u>(*Z*)-Trimethyl((3-phenyl-1-(4-(trifluoromethyl)phenyl)prop-1-en-1-yl)oxy)silane</u> (5c)

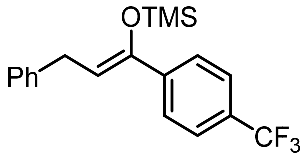

Following the **Procedure B**, compound **5c** was obtained in >95% yield as sticky oil.

^{1}H NMR (600 MHz, $CDCl_3$) δ 7.67 – 7.48 (m, 4H), 7.37 – 7.12 (m, 5H), 5.54 (t, *J* = 7.2 Hz, 1H), 3.58 (d, *J* = 7.2 Hz, 2H), 0.19 – 0.16 (m, 9H).

^{13}C NMR (151 MHz, $CDCl_3$) δ 148.5, 142.4, 140.9, 128.5 (2C), 128.4(2C), 126.0, 125.6 (2C), 125.1 (q, *J* = 3.8 Hz), 112.1, 32.4, 0.6.

HRMS (ESI): $[M+H]^+$ calculated for $C_{19}H_{22}F_3OSi$ 351.1387; found: 351.1383.

FT–IR (neat): 1644, 1616, 1409, 1322, 1220, 1164, 1123, 1108, 1067, 1038, 839, 776, 896 cm^{-1}.

<u>(*Z*)-((1-(4-(Tert-butyl)phenyl)-3-phenylprop-1-en-1-yl)oxy)trimethylsilane</u> (**5e**)

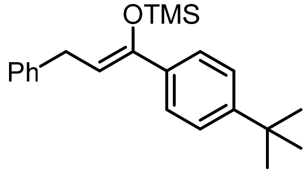

Following the **Procedure B**, compound **5e** was obtained as sticky oil in >95% yield

^{1}H NMR (400 MHz, $CDCl_3$) δ 7.30 – 7.22 (m, 2H), 7.17 – 6.98 (m, 7H), 5.23 (t, *J* = 7.2 Hz, 1H), 3.41 (dd, *J* = 14.5, 6.7 Hz, 2H), 1.15 (s, 9H), 0.00 (s, 9H).

^{13}C NMR (101 MHz, $CDCl_3$) δ = 156.6, 146.8, 141.5, 129.8, 128.6, 128.0, 127.8, 125.2, 119.7, 112.0, 110.4, 54.9, 31.5, -0.0.

HRMS (ESI): $[M+H]^+$ calculated for $C_{22}H_{31}OSi$ 339.2139; found: 339.2130.

FT–IR (neat): 1644, 1494, 1453, 1405, 1328, 1251, 1220, 1113, 1071, 1038, 1014, 837, 778 697 cm^{-1}.

(*Z*)-((1-(3-Fluorophenyl)-3-phenylprop-1-en-1-yl)oxy)trimethylsilane (**5f**)

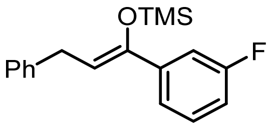

Following the **Procedure B**, compound **5f** was obtained as sticky oil in >95% yield

^{1}H NMR (600 MHz, $CDCl_3$) δ 7.33 – 7.24 (m, 6H), 7.23 – 7.18 (m, 2H), 6.98 – 6.93 (m, 1H), 5.47 (t, *J* = 7.2 Hz, 1H), 3.57 (d, *J* = 7.2 Hz, 2H), 0.18 (s, 9H).

^{13}C NMR (151 MHz, $CDCl_3$) δ = 163.6, 162.0, 148.6 (d, *J* = 2.6 Hz), 141.4 (d, *J* = 7.5 Hz), 141.1, 129.53 (d, *J* = 8.3 Hz), 128.41 (d, *J* = 7.4 Hz), 126.0, 121.03 (d, *J* = 2.7 Hz), 114.38 (d, *J* = 21.4 Hz), 112.5, 112.3, 112.38 (d, *J* = 22.7 Hz), 110.9, 32.4, 0.6.

HRMS (ESI): $[M+H]^+$ calculated for C18H22FOSi 301.1418; found: 301.1421.

FT–IR (neat): 1615, 1611, 1485, 1438, 1342, 1252, 1220, 1156, 1096, 1071, 1040, 965, 916, 839, 765, 696 cm^{-1}.

(*Z*)-Trimethyl((3-phenyl-1-(thiophen-2-yl)prop-1-en-1-yl)oxy)silane (**5i**)

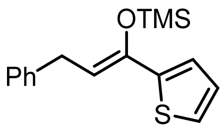

Following the **Procedure B**, compound **5i** was obtained as sticky oil in >95% yield

^{1}H NMR (600 MHz, $CDCl_3$) δ 7.33 – 7.28 (m, 2H), 7.26 (t, *J* = 3.4 Hz, 2H), 7.21 (t, *J* = 7.2 Hz, 1H), 7.15 – 7.11 (m, 1H), 7.09 (dd, *J* = 3.6, 1.1 Hz, 1H), 6.95 (dd, *J* = 5.0, 3.6 Hz, 1H), 5.44 (t, *J* = 7.3 Hz, 1H), 3.54 (d, *J* = 7.3 Hz, 2H), 0.25 (s, 9H).

^{13}C NMR (151 MHz, $CDCl_3$) δ = 144.6, 143.3, 141.0, 128.4 (2C), 127.1, 125.9, 124.1, 123.3, 109.0, 32.2, 0.7.

HRMS (ESI): $[M]^+$ calculated for $C_{16}H_{20}OSSi$ 288.1004; found: 288.0941.

FT–IR (neat): 1640, 1495, 1434, 1359, 1341, 1251, 1220, 1095, 1070, 1025, 839, 772, 693 cm^{-1}.

(*Z*)-((1-(Benzo[d][1,3]dioxol-5-yl)-3-phenylprop-1-en-1-yl)oxy)trimethylsilane (**5j**)

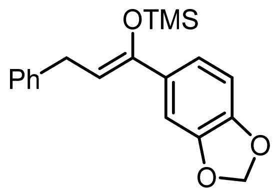

Following the **Procedure B**, compound **5j** was obtained as sticky oil in >95% yield

^{1}H NMR (600 MHz, $CDCl_3$) δ 7.34 – 7.11 (m, 5H), 7.04 – 6.93 (m, 2H), 6.76 (dd, *J* = 13.7, 8.1 Hz, 1H), 5.96 (d, *J* = 9.4 Hz, 2H), 5.29 (t, *J* = 7.2 Hz, 1H), 3.54 (d, *J* = 7.2 Hz, 2H), 0.16 (s, 9H).

^{13}C NMR (151 MHz, $CDCl_3$) δ = 149.4, 147.4, 147.2, 141.5, 133.5, 128.4, 128.4, 125.8, 119.3, 108.7, 107.8, 106.2, 101.0, 32.3, 0.6.

HRMS (ESI): $[M+H]^+$ calculated for $C_{19}H_{23}O_3Si$ 327.1411; found: 327.1408.

FT–IR (neat): 1646, 1486, 1438, 1357, 1288, 1248, 1220, 1141, 1107, 1069, 1035, 937, 839, 772, 697 cm^{-1}.

(*Z*)-Trimethyl((1-phenyl-3-(4-(trifluoromethyl)phenyl)prop-1-en-1-yl)oxy)silane (**5m**)

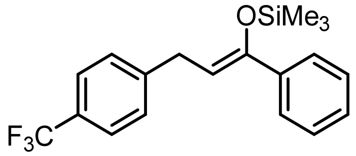

Following the **Procedure B**, compound **5m** was obtained as sticky oil in >95% yield

^{1}H NMR (600 MHz, $CDCl_3$) δ 7.50 (d, *J* = 7.3 Hz, 2H), 7.31 (t, *J* = 7.4 Hz, 2H), 7.28 – 7.22 (m, 2H), 7.04 (d, *J* = 7.6 Hz, 1H), 6.97 (d, *J* = 10.0 Hz, 1H), 6.88 (td, *J* = 8.5, 2.4 Hz, 1H), 5.38 (t, *J* = 7.2 Hz, 1H), 3.56 (d, *J* = 7.2 Hz, 2H), 0.15 (s, 9H).

^{13}C NMR (151 MHz, $CDCl_3$) δ 150.5, 144.08 (d, *J* = 7.1 Hz), 138.9, 129.69 (d, *J* = 8.3 Hz), 128.2, 127.9, 125.8, 123.99 (d, *J* = 2.7 Hz), 115.22 (d, *J* = 21.0 Hz), 112.70 (d, *J* = 21.1 Hz), 109.0, 32.2, 0.8.

^{19}F NMR (565 MHz, $CDCl_3$) δ -113.77, -113.78, -113.78, -113.80, -113.80, -113.81.

HRMS (ESI): $[M+Na]^+$ calculated for $C_{20}H_{26}NaO_3Si^+$ 365.1543; found 365.1543.

FT–IR (neat): 1646, 1615, 1589, 1487, 1446, 1337, 1251, 1220, 1074, 1041, 837, 770 686 cm^{-1}.

(*Z*)-Trimethyl((1,3,3-triphenylprop-1-en-1-yl)oxy)silane (5n)

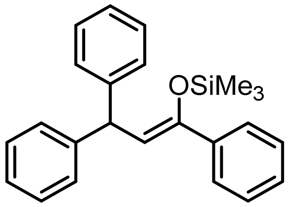

Following the **Procedure B**, compound **5n** was obtained as sticky oil in >95% yield

^{1}H NMR (600 MHz, $CDCl_3$) δ 7.57 – 7.49 (m, 2H), 7.36 – 7.24 (m, 10H), 7.23 – 7.12 (m, 3H), 5.77 – 5.64 (m, 1H), 5.24 (dd, *J* = 9.9, 2.5 Hz, 1H), 0.06 (dd, *J* = 4.5, 1.9 Hz, 9H).

^{13}C NMR (151 MHz, $CDCl_3$) δ = 149.6, 145.0, 139.1, 128.4, 128.3, 128.1, 127.9, 126.1, 126.0, 113.6, 47.2, 0.7.

HRMS: we cannot find desired peak by HRMS (ESI or EI).

FT–IR (neat): 1641, 1600, 1493, 1447, 1252, 1220, 1073, 1046, 1025, 922, 899, 842, 761, 729, 693, 674 cm^{-1}.

4. Oxidative C-C bond activation

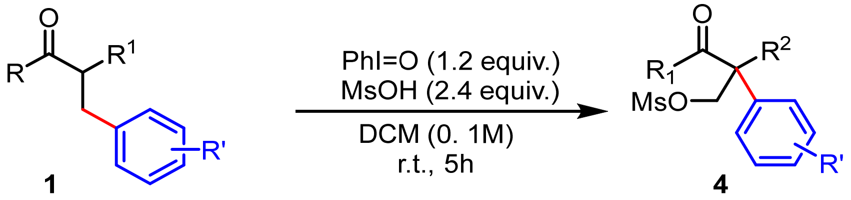

General procedure C: Carbonyl compounds **1** (0.2 mmol) was dissolved in dry DCM (2mL). Then iodosobenzene (0.24 mmol, 1.2 equiv.) and methanesulfonic acid (0.48 mmol, 2.4 equiv.) were added. The mixtures were stirred vigorously at room temperature until the starting material was completely consumed. After addition of *sat.* Na_2CO_3 (10 mL), the product was extracted with DCM (3 x 10 mL). The combined organic layers dried with $MgSO_4$, concentrated and purified by column chromatography (silica gel to afford the desired products).

Ethyl 2-(((methylsulfonyl)oxy)methyl)-3-oxo-2-phenylbutanoate (4a)

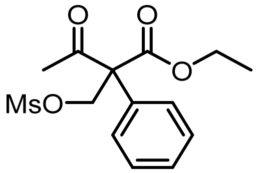

Following the **Procedure C**, compound **4a** obtained in 81% yield as colorless oil after column chromatography (3:1 of heptane:ethyl acetate).

^{1}H NMR (600 MHz, $CDCl_3$) δ 7.47 – 7.35 (m, 3H), 7.28 – 7.19 (m, 2H), 4.95 (d, *J* = 9.7 Hz, 1H), 4.69 – 4.59 (m, 1H), 4.47 – 4.33 (m, 2H), 2.97 (s, 3H), 2.23 (s, 3H), 1.37 (t, *J* = 7.1 Hz, 3H).

^{13}C NMR (151 MHz, $CDCl_3$) δ 201.5, 168.1, 133.8, 129.2, 128.8, 127.6, 70.9, 68.3, 62.5, 37.2, 28.1, 14.0.

HRMS (ESI): $[M+Na]^+$ calculated for $C_{14}H_{18}O_6SNa$ 337.0716; found: 337.0719.

FT-IR: 1736, 1680, 1448, 1358, 1240, 1220, 1176, 1099, 965, 772 cm^{-1}.

2-(Diethylcarbamoyl)-3-oxo-2-phenylbutyl methanesulfonate (4b)

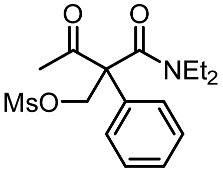

Following the **Procedure C**, compound **4b** obtained in 85% yield as colorless oil after column chromatography (1:1 of heptane:EtOAc).

^{1}H NMR (600 MHz, $CDCl_3$) δ 7.40 – 7.34 (m, 2H), 7.34 – 7.24 (m, 3H), 4.02 (d, *J* = 14.8 Hz, 1H), 3.72 (d, *J* = 14.8 Hz, 1H), 3.58 – 3.48 (m, 1H), 3.15 (s, 3H), 3.14 – 3.00 (m, 2H), 2.73 (dq, *J* = 14.0, 6.9 Hz, 1H), 2.33 (s, 3H), 1.08 (t, *J* = 7.1 Hz, 3H), 1.04 (t, *J* = 7.0 Hz, 3H).

^{13}C NMR (151 MHz, $CDCl_3$) δ = 201.0, 165.1, 133.8, 130.8, 128.4, 127.5, 95.2, 42.1, 41.2, 41.2, 40.8, 27.4, 13.7, 11.7.

HRMS (ESI): $[M+Na]^+$ calculated for $C_{19}H_{21}NONa$ 302.1515; found: 302.1517.

FT-IR: 1722, 1627, 1434, 1390, 1177, 1081, 960, 918, 889, 855, 703 cm^{-1}.

Benzyl 2-(((methylsulfonyl)oxy)methyl)-3-oxo-2-phenylbutanoate (4c)

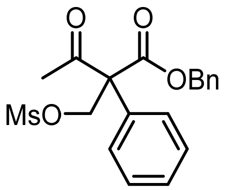

Following the **Procedure C**, Compound **4c** obtained in 83% yield as colorless oil after column chromatography (4:1 of heptane:EtOAc).

^{1}H NMR (400 MHz, $CDCl_3$) δ 7.40 – 7.23 (m, 8H), 7.14 – 7.05 (m, 2H), 5.26 (s, 2H), 4.87 (d, *J* = 9.7 Hz, 1H), 4.56 (d, *J* = 9.7 Hz, 1H), 2.78 (s, 3H), 2.08 (s, 3H).

^{13}C NMR (101 MHz, $CDCl_3$) δ = 201.2, 168.0, 134.6, 133.6, 129.2, 128.8, 128.8, 128.7, 128.7, 127.6, 70.8, 68.4, 68.1, 37.2, 28.1.

HRMS (ESI): $[M+Na]^+$ calculated for $C_{19}H_{20}O_6SNa$ 399.0873; found: 399.0843.

FT-IR: 1738, 1715, 1359, 1220, 1177, 994, 965, 822, 773 cm^{-1}.

Methyl 3-cyclopropyl-2-(((methylsulfonyl)oxy)methyl)-3-oxo-2-phenylpropanoate (**4d**)

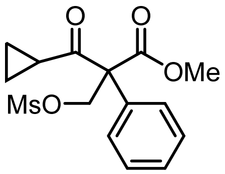

Following the **Procedure C**, compound **4d** obtained in 88% yield as colorless oil after column chromatography (3:1 of heptane:EtOAc).

^{1}H NMR (400 MHz, $CDCl_3$) δ 7.41 – 7.27 (m, 3H), 7.25 – 7.15 (m, 2H), 4.84 (d, *J* = 9.9 Hz, 1H), 4.58 (d, *J* = 9.9 Hz, 1H), 3.82 (s, 3H), 2.79 (s, 3H), 2.04 – 1.90 (m, 1H), 1.13 – 0.97 (m, 2H), 0.95 – 0.84 (m, 1H), 0.84 – 0.73 (m, 1H).

^{13}C NMR (101 MHz, $CDCl_3$) δ = 203.8, 168.7, 134.3, 129.0, 128.6, 128.3, 71.3, 68.6, 53.2, 37.1, 20.3, 13.3, 13.2.

HRMS (ESI): $[M+Na]^+$ calculated for $C_{15}H_{18}O_6SNa$ 349.0716; found: 349.0716.

FT-IR: 1738, 1699, 1358, 1220, 1176, 966, 941, 824, 773 cm^{-1}.

Ethyl 4-methyl-2-(((methylsulfonyl)oxy)methyl)-3-oxo-2-phenylpentanoate (**4e**)

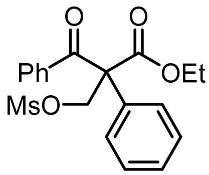

Following the **Procedure C**, compound **4e** obtained in 71% yield as colorless oil after column chromatography (3:1 of heptane:EtOAc).

^{1}H NMR (600 MHz, $CDCl_3$) δ 7.64 (dd, *J* = 8.4, 1.1 Hz, 2H), 7.52 – 7.46 (m, 2H), 7.38 (t, *J* = 7.4 Hz, 1H), 7.33 – 7.25 (m, 2H), 7.25 – 7.14 (m, 3H), 4.98 (d, *J* = 9.8 Hz, 1H), 4.58 (d, *J* = 9.8 Hz, 1H), 4.19 – 4.01 (m, 2H), 2.73 (s, 3H), 0.98 (t, *J* = 7.1 Hz, 3H).

^{13}C NMR (151 MHz, $CDCl_3$) δ = 193.6, 168.2, 135.0, 134.9, 133.2, 129.9, 128.9, 128.5, 128.4, 128.3, 74.2, 65.6, 62.4, 37.0, 13.6.

HRMS (ESI): $[M+Na]^+$ calculated for $C_{19}H_{20}O_6SNa$ 399.0872; found: 399.0872.

Ethyl 2-(((methylsulfonyl)oxy)methyl)-3-oxo-2-phenylheptanoate (**4f**)

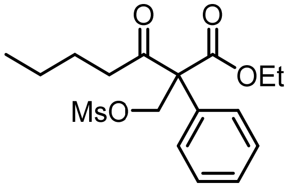

Following the **Procedure C**, compound **4f** obtained in 78% yield as colorless oil after column chromatography (4:1 of heptane:EtOAc).

^{1}H NMR (400 MHz, $CDCl_3$) δ 7.46 – 7.35 (m, 3H), 7.28 – 7.23 (m, 2H), 4.98 (d, *J* = 9.7 Hz, 1H), 4.65 (d, *J* = 9.7 Hz, 1H), 4.46 – 4.31 (m, 2H), 2.95 (s, 3H), 2.68 – 2.56 (m, 1H), 2.45 – 2.33 (m, 1H), 1.63 – 1.41 (m, 2H), 1.37 (t, *J* = 7.1 Hz, 3H), 1.25 – 1.12 (m, 2H), 0.89 – 0.75 (m, 3H).

^{13}C NMR (101 MHz, $CDCl_3$) δ = 204.0, 168.3, 133.9, 129.1, 128.7, 127.7, 71.2, 68.2, 62.4, 40.0, 37.2, 26.4, 22.0, 14.0, 13.7.

HRMS (ESI): $[M+Na]^+$ calculated for $C_{17}H_{24}O_6SNa$ 379.1186; found: 379.1187.

FT-IR: 1734, 1714, 1449, 1220, 1177, 1000, 966, 819, 773, 760 cm^{-1}.

2-Benzoyl-3-oxo-2-phenylbutyl methanesulfonate (4j)

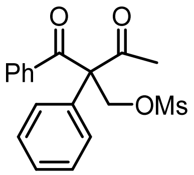

Following the **Procedure C**, compound **4j** obtained in 76% yield as colorless oil after column chromatography (2:1 of heptane:EtOAc).

^{1}H NMR (600 MHz, $CDCl_3$) δ 7.75 – 7.66 (m, 2H), 7.59 – 7.48 (m, 1H), 7.46 – 7.38 (m, 3H), 7.38 – 7.30 (m, 4H), 5.06 (q, *J* = 10.1 Hz, 2H), 2.86 (s, 3H), 2.27 (s, 3H).

^{13}C NMR (151 MHz, $CDCl_3$) δ = 202.3, 196.2, 135.2, 135.1, 133.6, 129.9, 129.23, 128.8, 128.6, 128.2, 71.6, 37.1, 28.6.

HRMS (ESI): $[M+Na]^+$ calculated for $C_{18}H_{18}O_5S$ 369.0767; found: 369.0767.

FT-IR: 1714, 1679, 1359, 1177, 966, 914, 773, 673 cm^{-1}.

<u>2-Cyano-3-oxo-2,3-diphenylpropyl methanesulfonate</u> (**4h**)

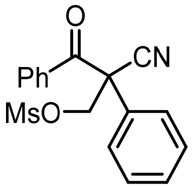

Following the **Procedure C**, compound **4h** obtained in 61% yield as white solid after column chromatography (3:1 of heptane:EtOAc).

^{1}H NMR (600 MHz, $CDCl_3$) δ 7.93 – 7.86 (m, 2H), 7.59 – 7.38 (m, 8H), 5.00 (d, *J* = 10.1 Hz, 1H), 4.53 (d, *J* = 10.1 Hz, 1H), 3.05 (s, 3H).

^{13}C NMR (151 MHz, $CDCl_3$) δ = 188.5, 134.5, 132.8, 130.8, 130.2, 130.1, 130.1, 128.7, 128.5, 126.4, 71.1, 56.4, 37.8.

HRMS (ESI): $[M+Na]^+$ calculated for $C_{17}H_{15}NO_4S$ 352.0614; found: 352.0614.

FT-IR: 2348, 1695, 1597, 1449, 1220, 1178, 1009, 969, 945, 914, 779 cm-1.

<u>Ethyl 2-(4-fluorophenyl)-2-(((methylsulfonyl)oxy)methyl)-3-oxobutanoate</u> (**4i**)

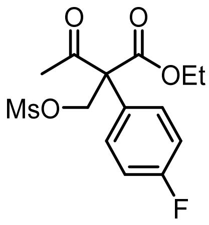

Following the **Procedure C**, compound **4i** obtained in 71% yield as colorless oil after column chromatography (3:1 of heptane:EtOAc).

^{1}H NMR (400 MHz, $CDCl_3$) δ 7.28 – 7.12 (m, 2H), 7.10 – 6.96 (m, 2H), 4.83 (d, *J* = 9.8 Hz, 1H), 4.54 (d, *J* = 9.8 Hz, 1H), 4.44 – 4.18 (m, 3H), 2.89 (3, 1H), 2.13 (s, 3H), 1.28 (t, *J* = 7.1 Hz, 3H).

^{13}C NMR (101 MHz, $CDCl_3$) δ 201.2, 168.0, 162.7 (d, *J* = 249.6 Hz), 129.7 (d, *J* = 8.3 Hz), 129.6 (d, *J* = 3.6 Hz), 116.2 (d, *J* = 21.7 Hz), 70.8, 67.7, 62.7, 37.3, 28.0, 14.0.

HRMS (ESI): $[M+Na]^+$ calculated for $C_{14}H_{17}FO_6SNa$ 355.0622; found: 355.0622.

FT-IR: 1734, 1715, 1512, 1358, 1220, 1177, 996, 966, 773 cm^{-1}.

Ethyl 2-(4-chlorophenyl)-2-(((methylsulfonyl)oxy)methyl)-3-oxobutanoate (**4j**)

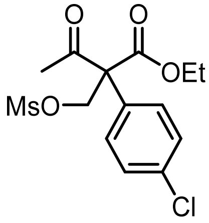

Following the **Procedure C**, compound **4j** obtained in 79% yield as colorless oil after column chromatography (3:1 of heptane:EtOAc).

^{1}H NMR (400 MHz, $CDCl_3$) δ 7.40 – 7.27 (m, 2H), 7.16 – 7.09 (m, 2H), 4.83 (d, *J* = 9.8 Hz, 1H), 4.54 (d, *J* = 9.8 Hz, 1H), 4.30 (qd, *J* = 7.1, 0.5 Hz, 2H), 2.89 (s, 3H), 2.13 (s, 3H), 1.27 (t, *J* = 7.1 Hz, 3H).

^{13}C NMR (101 MHz, $CDCl_3$) δ 200.9, 167.8, 135.0, 132.2, 129.4, 129.2, 70.6, 67.8, 62.7, 37.3, 28.0, 14.0.

HRMS (ESI): $[M+Na]^+$ calculated for C14H17ClO6SNa 371.0327; found: 371.0326.

FT-IR: 1735, 1716, 1517, 1473, 1314, 1127, 914, 895, 884, 703 cm-1.

Ethyl 2-(4-bromophenyl)-2-(((methylsulfonyl)oxy)methyl)-3-oxobutanoate (**4k**)

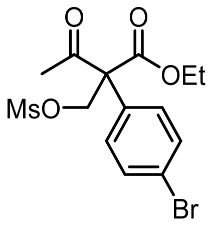

Following the **Procedure C**, compound **4k** obtained in 73% yield as colorless oil after column chromatography (3:1 of heptane:EtOAc).

^{1}H NMR (400 MHz, $CDCl_3$) δ 7.53 – 7.41 (m, 2H), 7.12 – 6.99 (m, 2H), 4.82 (d, *J* = 9.8 Hz, 1H), 4.54 (d, *J* = 9.8 Hz, 1H), 4.37 – 4.22 (m, 2H), 2.89 (s, 3H), 2.13 (s, 3H), 1.27 (t, *J* = 7.1 Hz, 3H).

^{13}C NMR (101 MHz, $CDCl_3$) δ = 200.8, 167.7, 132.8, 132.3, 129.5, 123.2, 70.5, 67.9, 62.6, 37.3, 28.0, 14.0.

HRMS (ESI): $[M+Na]^+$ calculated for $C_{14}H_{17}BrO_6SNa$; found: 414.9821; found: 414.9821.

FT-IR: 1734, 1714, 1492, 1356, 1294, 1220, 1175, 1010, 965, 822, 773 cm^{-1}.

Ethyl 2-([1,1'-biphenyl]-4-yl)-2-(((methylsulfonyl)oxy)methyl)-3-oxobutanoate (**4l**)

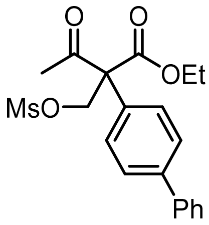

Following the **Procedure C**, compound **4l** obtained in 65% yield as colorless oil after column chromatography (3:1 of heptane:EtOAc).

^{1}H NMR (400 MHz, $CDCl_3$) δ 7.70 – 7.58 (m, 5H), 7.58 – 7.43 (m, 2H), 7.44 – 7.31 (m, 2H), 5.00 (d, *J* = 9.7 Hz, 1H), 4.70 (d, *J* = 9.7 Hz, 1H), 4.47 – 4.38 (m, 2H), 3.00 (s, 3H), 2.28 (s, 3H), 1.45 – 1.36 (m, 3H).

^{13}C NMR (101 MHz, $CDCl_3$) δ = 201.4, 168.2, 141.7, 139.8, 132.6, 128.9, 128.8, 128.7, 128.0, 127.8, 127.8, 127.1, 127.1, 126.8, 70.8, 68.1, 62.6, 37.3, 28.2, 14.0.

HRMS (ESI): $[M+Na]^+$ calculated for $C_{20}H_{22}O_6SNa$ 413.1029; found: 413.1031.

FT-IR: 1734, 1715, 1488, 1358, 1297, 1220, 1177, 1008, 995, 965, 932, 772, 699 cm^{-1}.

Ethyl 2-(4-methoxyphenyl)-2-(((methylsulfonyl)oxy)methyl)-3-oxobutanoate (**4m**)

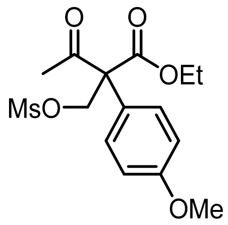

Following the **Procedure C**, compound **4m** obtained in 50% yield as colorless oil after column chromatography (3:1 of heptane:EtOAc).

^{1}H NMR (400 MHz, $CDCl_3$) δ 7.13 – 7.04 (m, 2H), 6.90 – 6.81 (m, 2H), 4.83 (d, *J* = 9.7 Hz, 1H), 4.52 (d, *J* = 9.6 Hz, 1H), 4.35 – 4.25 (m, 2H), 3.74 (s, 3H), 2.89 (s, 2H), 2.13 (s, 2H), 1.28 (t, *J* = 7.1 Hz, 3H).

^{13}C NMR (101 MHz, $CDCl_3$) δ = 201.7, 168.4, 159.8, 128.8, 125.5, 114.6, 71.0, 67.6, 62.4, 55.3, 37.3, 28.0, 14.0.

HRMS (ESI): $[M+Na]^+$ calculated for $C_{15}H_{20}O_7SNa$ 367.0822; found: 367.0822.

FT-IR: 1735, 1714, 1514, 1358, 1256, 1220, 1176, 830, 773 cm^{-1}.

Ethyl 2-(4-isobutylphenyl)-2-(((methylsulfonyl)oxy)methyl)-3-oxobutanoate (**4n**)

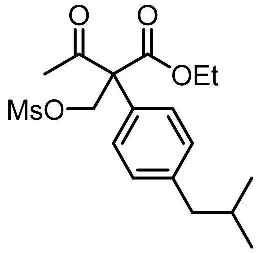

Following the **Procedure C**, compound **4n** obtained in 66% yield as colorless oil after column chromatography (3:1 of heptane:EtOAc).

^{1}H NMR (400 MHz, $CDCl_3$) δ 7.16 – 6.97 (m, 4H), 4.85 (d, *J* = 9.6 Hz, 1H), 4.53 (d, *J* = 9.6 Hz, 1H), 4.40 – 4.23 (m, 2H), 2.87 (s, 3H), 2.40 (d, *J* = 7.2 Hz, 2H), 2.13 (s, 3H), 1.88 – 1.68 (m, 1H), 1.28 (t, *J* = 7.1 Hz, 3H), 0.83 (t, *J* = 6.9 Hz, 6H).

^{13}C NMR (101 MHz, $CDCl_3$) δ = 201.7, 168.3, 142.6, 130.9, 129.9, 127.2, 71.0, 68.0, 62.4, 44.9, 37.2, 30.1, 28.1, 22.3, 14.0.

HRMS (ESI): $[M+Na]^+$ calculated for $C_{18}H_{26}O_6SNa$ 393.1342; found: 393.1341.

FT-IR: 1735, 1716, 1359, 1220, 1177, 995, 965, 932, 773 cm^{-1}.

Ethyl 2-(fluoromethyl)-3-oxo-2-phenylbutanoate (**4o**)

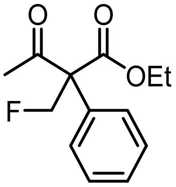

Following the **Procedure C**, Pyridine·9HF (0.1 mL) was used instead of $MeSO_3H$.

Compound **4o** obtained in 53% yield as colorless oil after column chromatography (4:1 of heptane:EtOAc).

^{1}H NMR (600 MHz, $CDCl_3$) δ 7.45 – 7.36 (m, 3H), 7.29 – 7.24 (m, 2H), 5.19 (dd, *J* = 46.4, 9.1 Hz, 1H), 4.95 (dd, *J* = 46.4, 9.1 Hz, 1H), 4.41 – 4.35 (m, 2H), 2.23 (s, 3H), 1.36 (t, *J* = 7.1, 3H).

^{13}C NMR (151 MHz, $CDCl_3$) δ = 202.14 (d, *J* = 2.0 Hz), 168.64 (d, *J* = 5.0 Hz), 140.5, 129.0, 128.4, 127.7, 84.2 (d, *J* = 178.0 Hz). 62.2, 61.6, 28.4, 14.0.

HRMS (ESI): $[M+Na]^+$ calculated for $C_{13}H_{15}FO_3$ 261.0897; found: 261.0899.

FT-IR: 1735, 1714, 1498, 1449, 1357, 1236, 1221, 1092, 1045, 1018, 773, 699 cm^{-1}.

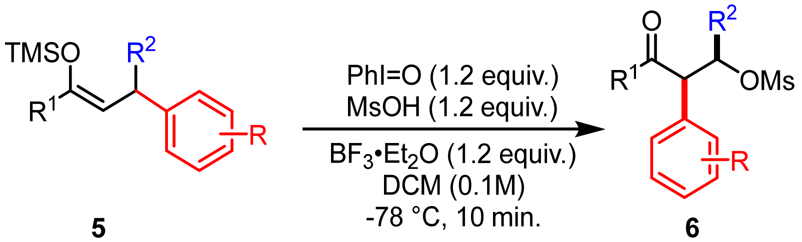

General procedure D: Iodosobenzene (0.24 mmol) was added to a flame dried flask, and DCM (2 mL) was added. Then MsOH (0.24 mmol) was slowly added and further stirred until all the yellow solid was dissolved. Then the flask was merged to a dry ice-acetone bath, and BF_3·Et_2O solution was slowly added and further stirred for 5 minutes. Finally, silica enonate **5** was added by one-and further stirred for 10 minutes at the same temperature. Then sat. $NaHCO_3$ solution was added to quench the reaction, the aqueous layer was extracted twice with EtOAc. The combined organic layers were washed with a saturated aqueous solution of $NaHCO_3$, then brine. The organic layer was dried over $MgSO_4$, and the solvent was evaporated under reduced pressure.

The crude mixture purified by column chromatography on silica gel with AcOEt/ hexanes (1:3) to provide desired products.

3-Oxo-2,3-diphenylpropyl methanesulfonate (**6a**)

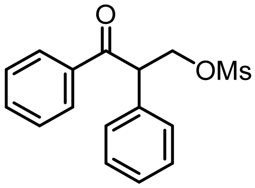

Following the **Procedure D**, compound **6a** obtained in 71% yield as white solid.

^{1}H NMR (400 MHz, $CDCl_3$) δ 7.96 (dt, *J* = 12.6, 6.1 Hz, 2H), 7.58 – 7.47 (m, 1H), 7.47 – 7.25 (m, 7H), 5.05 (dd, *J* = 9.1, 5.0 Hz, 1H), 4.99 – 4.90 (m, 1H), 4.45 (dd, *J* = 9.7, 5.0 Hz, 1H), 2.99 (s, 3H).

^{13}C NMR (101 MHz, $CDCl_3$) δ = 196.3, 135.7, 134.1, 133.6, 129.5, 128.8, 128.7, 128.5, 128.4, 70.4, 52.8, 37.1.

HRMS (ESI): $[M+Na]^+$ calculated for $C_{16}H_{16}O_4SNa$ 327.0662; found: 327.0667.

FT-IR: 1680, 1450, 1345, 1268, 1219, 1171, 979, 957, 917, 88, 828, 772, 696 cm^{-1}.

3-(4-fluorophenyl)-3-oxo-2-phenylpropyl methanesulfonate (**6b**)

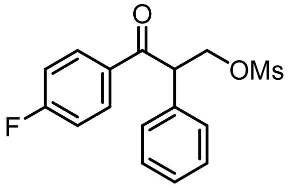

Following the **Procedure D**, compound **6b** obtained in 72% yield as sticky oil.

^{1}H NMR (600 MHz, $CDCl_3$) δ 8.00 – 7.89 (m, 2H), 7.35 (dd, *J* = 10.4, 4.4 Hz, 2H), 7.33 – 7.28 (m, 3H), 7.11 – 7.03 (m, 2H), 4.97 (dd, *J* = 9.1, 5.0 Hz, 1H), 4.91 (t, *J* = 9.4 Hz, 1H), 4.42 (dd, *J* = 9.7, 5.0 Hz, 1H), 2.98 (s, 3H).

^{13}C NMR (151 MHz, $CDCl_3$) δ 194.8, 166.1 (d, *J* = 256.3 Hz), 134.0, 132.2, 132.2, 131.7 (d, *J* = 9.5 Hz), 129.7, 128.7 (d, *J* = 3.2 Hz), 116.1 (d, *J* = 22.0 Hz), 70.4, 53.0, 37.3.

HRMS (ESI): $[M+Na]^+$ calculated for $C_{16}H_{15}FNaO_4S$ 345.0567; found: 345.0567.

FT-IR: 1686, 1588, 1486, 1442, 1354, 1264, 1220, 1173, 956, 779, 700 674 cm^{-1}.

3-oxo-2-phenyl-3-(4-(trifluoromethyl)phenyl)propyl methanesulfonate (**6c**)

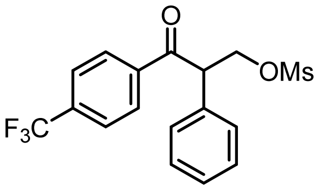

Following the **Procedure D**, compound **6c** obtained in 48% yield as sticky oil.

^{1}H NMR (600 MHz, $CDCl_3$) δ 8.04 (d, *J* = 8.2 Hz, 2H), 7.66 (d, *J* = 8.3 Hz, 2H), 7.38 – 7.27 (m, 5H), 5.02 (dd, *J* = 9.1, 5.1 Hz, 1H), 4.92 (t, *J* = 9.6 Hz, 1H), 4.44 (dd, *J* = 10.0, 5.1 Hz, 1H), 2.99 (s, 3H).

^{13}C NMR (151 MHz, $CDCl_3$) δ 195.5, 138.4, 134.9 (q, *J* = 32.9 Hz), 133.5, 129.9, 129.3, 128.9, 128.6, 125.9 (q, *J* = 3.6 Hz), 123.5 (q, *J* = 273 Hz), 70.1, 53.4, 37.3.

HRMS (ESI): $[M+Na]^+$ calculated for $C_{17}H_{15}F_3NaO_4S$ 395.0535; found: 395.0537.

FT-IR: 1691, 1359, 1324, 1220, 1173, 1130, 1067, 956, 775 cm^{-1}.

3-(4-methoxyphenyl)-3-oxo-2-phenylpropyl methanesulfonate (**6d**)

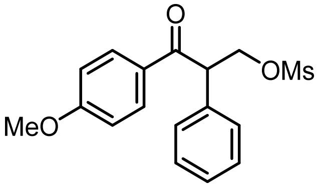

Following the **Procedure D**, compound **6d** obtained in 91% yield as sticky oil.

^{1}H NMR (400 MHz, $CDCl_3$) δ 7.90 – 7.79 (m, 2H), 7.30 – 7.13 (m, 5H), 6.86 – 6.68 (m, 2H), 4.89 (dd, *J* = 9.0, 4.5 Hz, 1H), 4.87 – 4.80 (m, 1H), 4.33 (dd, *J* = 9.1, 4.5 Hz, 1H), 3.73 (s, 3H), 2.88 (s, 3H).

^{13}C NMR (101 MHz, $CDCl_3$) δ 194.8, 164.0, 134.7, 131.3, 129.5, 128.8, 128.5, 128.4, 114.1, 70.8, 55.59, 52.6, 37.2.

HRMS (ESI): $[M+Na]^+$ calculated for $C_{17}H_{18}NaO_5S$ 357.0767; found: 357.0769.

FT-IR: 1672, 1599, 1511, 1455, 1260, 1220, 1170, 1028, 954, 876, 816, 773, 702 cm^{-1}.

3-(4-(*Tert*-butyl)phenyl)-3-oxo-2-phenylpropyl methanesulfonate (**6e**)

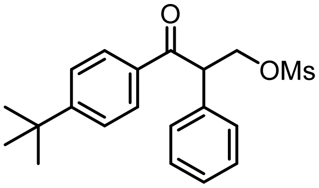

Following the **Procedure D**, compound **6e** obtained in 61% yield as sticky oil.

^{1}H NMR (400 MHz, $CDCl_3$) δ 7.93 – 7.86 (m, 2H), 7.46 – 7.39 (m, 2H), 7.37 – 7.26 (m, 5H), 5.01 (dd, *J* = 9.2, 5.0 Hz, 1H), 4.96 – 4.90 (m, 1H), 4.42 (dd, *J* = 9.6, 5.0 Hz, 1H), 2.96 (s, 3H), 1.29 (s, 9H).

^{13}C NMR (101 MHz, $CDCl_3$) δ 196.0, 157.6, 134.4, 133.3, 129.6, 129.0, 128.6, 128.5, 125.9, 70.7, 52.80, 37.2, 35.3, 31.1.

HRMS (ESI): $[M+Na]^+$ calculated for $C_{20}H_{24}NaO_4S$ 383.1288; found: 383.1288.

FT-IR: 2963, 1676, 1604, 1455, 1409, 1356, 1267, 1220, 1173, 1110, 953, 890, 812, 773 $cm^{-1.}$

3-(3-Fluorophenyl)-3-oxo-2-phenylpropyl methanesulfonate (**6f**)

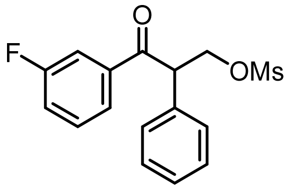

Following the **Procedure D**, compound **6f** obtained in 68% yield as sticky oil.

^{1}H NMR (400 MHz, $CDCl_3$) δ 7.77 – 7.65 (m, 1H), 7.66 – 7.58 (m, 1H), 7.45 – 7.14 (m, 7H), 4.97 (dd, *J* = 9.1, 4.7 Hz, 1H), 4.91 (t, *J* = 9.1 Hz, 1H), 4.42 (dd, *J* = 9.5, 4.7 Hz, 1H), 2.97 (s, 3H).

^{13}C NMR (101 MHz, $CDCl_3$) δ 195.1, 162.8 (d, *J* = 248.5 Hz), 137.8 (d, *J* = 6.3 Hz), 133.6, 130.4 (d, *J* = 7.7 Hz), 129.6, 128.6, 128.4, 124.6 (d, *J* = 3.0 Hz), 120.7 (d, *J* = 21.5 Hz), 115.5 (d, *J* = 22.5 Hz), 70.2, 53.1, 37.1.

HRMS (ESI): $[M+Na]^+$ calculated for $C_{16}H_{15}FNaO_4S$ 345.0567; found: 345.0568.

FT-IR: 1685, 1588, 1486, 1442, 1354, 1264, 1220, 1173, 956, 881, 820, 773, 700 674 cm^{-1}.

3-([1,1'-Biphenyl]-4-yl)-3-oxo-2-phenylpropyl methanesulfonate (**6g**)

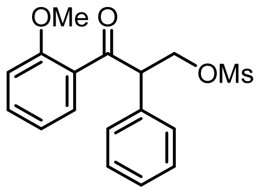

Following the **Procedure D**, compound **6g** obtained in 58% yield as sticky oil.

^{1}H NMR (400 MHz, $CDCl_3$) δ 7.62 (dd, *J* = 7.7, 1.8 Hz, 1H), 7.34 (ddd, *J* = 8.4, 7.3, 1.8 Hz, 1H), 7.25

– 7.13 (m, 5H), 6.87 (td, *J* = 7.7, 0.9 Hz, 1H), 6.80 (d, *J* = 8.4 Hz, 1H), 5.13 (dd, *J* = 8.8, 5.6 Hz, 1H), 4.91 – 4.74 (m, 1H), 4.31 (dd, *J* = 9.8, 5.6 Hz, 1H), 3.76 (s, 3H), 2.86 (s, 3H).

^{13}C NMR (101 MHz, $CDCl_3$) δ 198.5, 158.6, 134.5, 134.4, 131.3, 129.0, 129.0, 128.1, 127.0, 120.9, 111.8, 71.2, 56.6, 55.5, 37.2.

HRMS (ESI): $[M+Na]^+$ calculated for $C_{17}H_{18}O_5SNa$ 357.0767; found: 357.0769.

FT-IR (neat): 1673, 1599, 1456, 1260, 1220, 1028, 954, 876, 816, 773, 701 cm^{-1}

<u>3-(3,5-dimethylphenyl)-3-oxo-2-phenylpropyl methanesulfonate</u> (**6h**)

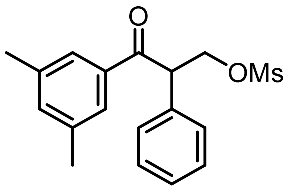

Following the **Procedure D**, compound **6h** obtained in 87% yield as sticky oil.

^{1}H NMR (400 MHz, $CDCl_3$) δ 7.47 (s, 2H), 7.30 – 7.15 (m, 5H), 7.10 – 7.06 (m, 1H), 4.94 (dd, *J* = 9.2, 5.0 Hz, 1H), 4.89 – 4.81 (m, 1H), 4.34 (dd, *J* = 9.7, 5.0 Hz, 1H), 2.89 (s, 3H), 2.24 (s, 3H), 2.24 (s, 3H).

^{13}C NMR (101 MHz, $CDCl_3$) δ 196.8, 138.5, 136.0, 135.5, 134.3, 129.6, 128.6, 128.5, 126.8, 70.7, 52.8, 37.2, 21.4.

HRMS (ESI): $[M+Na]^+$ calculated for $C_{18}H_{20}NaO_4S$ 355.0975; found: 355.0974.

FT-IR: 1676, 1601, 1493, 1454, 1355, 1297, 1220, 1174, 1047, 957, 889, 815 772, 701 cm^{-1}.

<u>3-oxo-2-phenyl-3-(thiophen-2-yl)propyl methanesulfonate</u> (**6i**)

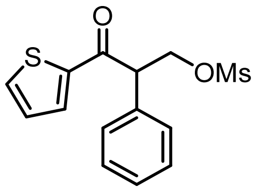

Following the **Procedure D**, compound **6i** obtained in >95% yield as sticky oil.

^{1}H NMR (400 MHz, $CDCl_3$) δ 7.70 (dd, *J* = 3.9, 1.0 Hz, 1H), 7.63 (dd, *J* = 4.9, 1.0 Hz, 1H), 7.42 –7.28 (m, 5H), 7.07 (dd, *J* = 4.9, 3.9 Hz, 1H), 4.95 – 4.76 (m, 2H), 4.41 (dd, *J* = 9.3, 4.5 Hz, 1H), 2.98 (s, 3H).

^{13}**C NMR** (101 MHz, $CDCl_3$) δ 189.3, 143.0, 134.8, 134.3, 133.4, 129.6, 128.7, 128.5, 128.5, 70.2, 54.2, 37.3.

HRMS (ESI): $[M+Na]^+$ calculated for $C_{14}H_{14}NaO_4S_2$ 333.0226; found: 333.0226.

FT-IR: 1657, 1413, 1355, 1220, 1174, 958, 815, 773 cm^{-1}.

3-(benzo[d][1,3]dioxol-5-yl)-3-oxo-2-phenylpropyl methanesulfonate (**6j**)

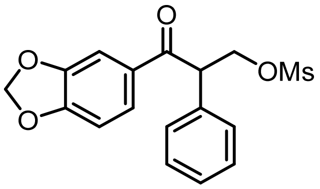

Following the **Procedure D**, compound **6j** obtained in 83% yield as sticky oil.

^{1}**H NMR** (400 MHz, $CDCl_3$) δ 7.40 – 7.36 (m, 1H), 7.25 (d, *J* = 1.7 Hz, 1H), 7.21 – 7.09 (m, 5H), 6.62 (d, *J* = 8.2 Hz, 1H), 5.84 (s, 2H), 4.79 – 4.69 (m, 2H), 4.30 – 4.18 (m, 1H), 2.80 (s, 3H).

^{13}**C NMR** (151 MHz, $CDCl_3$) δ 194.4, 152.3, 148.4, 134.5, 130.6, 129.6, 128.5, 128.5, 125.6, 108.6, 108.2, 102.1, 70.7, 52.7, 37.2.

HRMS (ESI): [M+Na]+ calculated for $C_{17}H_{16}NaO_6S$ 371.0560; found: 371.0561.

FT-IR: 1671, 1604, 1490, 1443, 1355, 1260, 1220, 1174, 1037, 957, 894, 808, 773 cm^{-1}.

4,4-dimethyl-3-oxo-2-phenylpentyl methanesulfonate (**6k**)

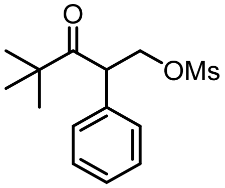

Following the **Procedure D**, compound **6k** obtained in >95% yield as sticky oil.

^{1}**H NMR** (600 MHz, $CDCl_3$) δ 7.36 – 7.22 (m, 5H), 4.68 (t, *J* = 9.8 Hz, 1H), 4.58 (dd, *J* = 9.8, 4.9 Hz, 1H), 4.22 (dd, *J* = 9.6, 4.9 Hz, 1H), 2.92 (s, 3H), 1.09 (s, 9H).

^{13}**C NMR** (151 MHz, $CDCl_3$) δ 212.4, 133.7, 129.4, 128.6, 128.4, 71.3, 51.9, 45.3, 37.2, 26.5.

HRMS (ESI): $[M+Na]^+$ calculated for $C_{14}H_{20}NaO_4S$ 307.0975; found: 307.0977.

FT-IR: 2971, 1704, 1358, 1220, 1175, 953, 684, 816, 773 cm^{-1}.

2-(3,4-dimethoxyphenyl)-3-oxo-3-phenylpropyl methanesulfonate (**6l**)

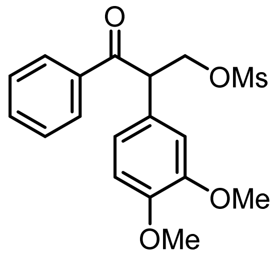

Following the **Procedure D**, compound **6l** obtained in 62% yield as sticky oil.

^{1}H NMR (600 MHz, $CDCl_3$) δ 7.94 (d, *J* = 7.4 Hz, 2H), 7.53 (t, *J* = 7.4 Hz, 1H), 7.41 (t, *J* = 7.8 Hz, 2H), 6.86 (dd, *J* = 8.3, 1.8 Hz, 1H), 6.81 (d, *J* = 8.3 Hz, 1H), 6.77 (d, *J* = 1.7 Hz, 1H), 4.96 (dd, *J* = 9.3, 4.7 Hz, 1H), 4.91 (t, *J* = 9.4 Hz, 1H), 4.41 (dd, *J* = 9.5, 4.7 Hz, 1H), 3.86 (s, 3H), 3.83 (s, 3H), 3.00 (s, 3H).

^{13}C NMR (151 MHz, $CDCl_3$) δ 196.6, 149.8, 149.3, 135.9, 133.7, 128.9, 128.9, 126.3, 121.2, 111.9, 111.1, 70.5, 56.2, 56.0, 52.5, 37.3.

HRMS (ESI): $[M+Na]^+$ calculated for $C_{18}H_{20}NaO_6S^+$ 387.0873; found: 387.0872.

FT–IR (neat): 2935, 1680, 1593, 1515, 1448, 1420, 1352, 1261, 1221, 1172, 1144, 1025, 952, 851, 817, 772, 890 cm^{-1}.

3-Oxo-3-phenyl-2-(4-(trifluoromethyl)phenyl)propyl methanesulfonate (**6m**)

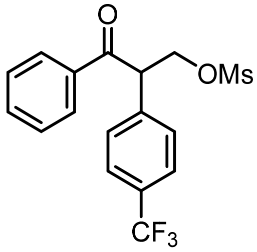

Following the **Procedure D**, compound **6m** obtained as sticky oil in 54% yield.

^{1}H NMR (600 MHz, $CDCl_3$) δ 7.93 (dd, *J* = 8.4, 1.2 Hz, 2H), 7.54 (dd, *J* = 10.6, 4.3 Hz, 1H), 7.43 (t, *J* = 7.8 Hz, 2H), 7.32 (td, *J* = 8.0, 5.9 Hz, 1H), 7.12 (d, *J* = 7.8 Hz, 1H), 7.05 – 7.02 (m, 1H), 7.01 – 6.96 (m, 1H), 5.03 (dd, *J* = 8.9, 5.3 Hz, 1H), 4.91 (dd, *J* = 9.9, 9.0 Hz, 1H), 4.43 (dd, *J* = 10.0, 5.3 Hz, 1H), 3.00 (s, 3H).

^{13}C NMR (151 MHz, $CDCl_3$) δ 195.8, 136.3 (d, *J* = 7.4 Hz), 135.5, 133.8, 131.1 (d, *J* = 8.3 Hz), 128.9, 128.8, 124.2 (d, *J* = 3.0 Hz), 123.5 (q, *J* = 273 Hz), 115.4 (q, J = 10.4 Hz) 70.0, 52.3, 37.2.

HRMS (ESI): $[M+Na]^+$ calculated for $C_{17}H_{16}F_3NaO_4S$ 395.0535; found: 395.0537.

FT–IR (neat): 1682, 1613, 1591, 1486, 1448, 1356, 1220, 1175, 1141, 960, 862, 774, 691 cm^{-1}.

<u>(1*S*,2*R*)-3-oxo-1,2,3-triphenylpropyl methanesulfonate</u> (**6n**)

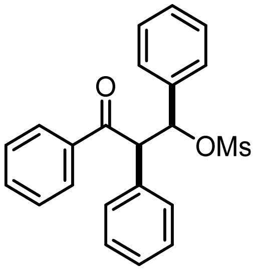

Following the **Procedure D**, compound **6n** obtained as white solid in 83% yield.

Based on previous report, we assign the structure was *syn* of diphenyl group. (*cf.* P. Singh, A. Bhardwaj, *J. Med. Chem.* **2010**, *53*, 3707–3717)

^{1}H NMR (600 MHz, $CDCl_3$) δ 8.05 – 7.97 (m, 2H), 7.55 (t, *J* = 7.4 Hz, 1H), 7.44 (t, *J* = 7.8 Hz, 2H), 7.26 – 7.17 (m, 5H), 7.17 – 7.11 (m, 3H), 7.08 (dd, *J* = 7.1, 2.5 Hz, 2H), 6.27 (d, *J* = 10.3 Hz, 1H), 5.09 (d, *J* = 10.3 Hz, 1H), 2.92 (s, 3H).

^{13}C NMR (151 MHz, $CDCl_3$) δ = 196.5, 136.6, 136.2, 133.5, 132.9, 129.1, 129.0 (2C), 128.8, 128.7, 128.4, 128.1, 127.2, 85.0, 59.5, 38.7.

HRMS (ESI): $[M+Na]^+$ calculated for $C_{22}H_{20}NaO_4S$ 403.0975; found: 403.0972.

FT-IR (neat): 1681, 1450, 1357, 1272, 1211, 1173, 913, 893, 828, 698, 609 cm^{-1}.

5. Enantioselective α-arylation of 5a

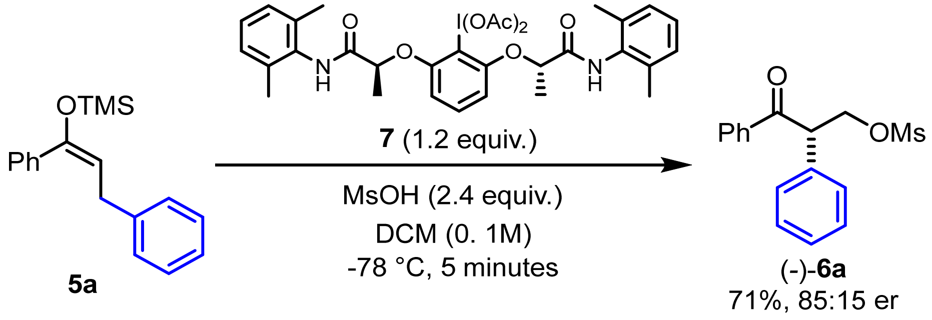

Hypervalent Iodine compound **7** was prepared according to previous reported procedure: M. Uyanik, T. Yasui, K. Ishihara, *Angew. Chem. Int. Ed.* **2010**, *49*, 2175

7 (0.24 mmol) was added to a flame dried flask, and DCM (2 mL) was added. Then MsOH (0.48 mmol) was slowly added and further stirred until all the yellow solid was dissolved. Then the flask

was merged to a dry ice-acetone bath and further stirred for 5 minutes. Finally, silica enonate **5a** was added and further stirred for 10 minutes at the same temperature. Then *sat.* NaHCO3 solution was added to quench the reaction, the aqueous layer was extracted twice with EtOAc. The combined organic layers were washed with a saturated aqueous solution of $NaHCO_3$, then brine. The organic layer was dried over MgSO4, and the solvent was evaporated under reduced pressure. The crude mixture was purified by column chromatography on silica gel with ethyl acetate/hexanes (1:3) to provide desired (-)-**6a** in 71% yield with 70% ee.

(**In here, we didn't conform the absolute configuration**)

HPLC analysis (Column: Chiralcel OD-H 250×4.6 mm; Solvent System: *n*-Heptane + 0.1% IPA: EtOH = 83:17); Flow: 0.70 mL/min, 254 nm) indicated > 99% ee (t_{minor} = 26.31 min, t_{major} = 30.39 min)

$[\alpha]_D^{20}$ = -73.5 (C = 1.0, $CHCl_3$)

6. Application

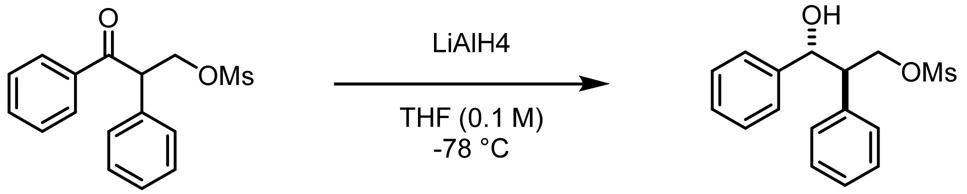

6a **8**

A solution of lithium aluminum hydride (1.0 M in THF; 0.30 mL, 0.30 mmol) was added over 2 min to a −78 °C solution of **6a** (0.2 mmol) in THF (2.0 mL) in a 10-mL round bottom flask under argon. The reaction mixture was allowed stirred at the same temperature for 1 h. Next, water (0.5 mL) was added dropwise to the reaction mixture at 0 °C to quench the reaction. Aqueous HCl (1.0 N; 5.0 mL) was added, and the mixture was extracted with EtOAc (3×5 mL). The combined organic extracts were dried over $MgSO_4$, filtered, and concentrated afforded the alcohol **8** as a white solid > 95% yield without further purification.

^{1}H NMR (600 MHz, $CDCl_3$) δ 7.35 – 7.23 (m, 6H), 7.22 – 7.12 (m, 4H), 5.10 (d, *J* = 6.3 Hz, 1H), 4.50 (dd, *J* = 10.0, 6.7 Hz, 1H), 4.34 (dd, *J* = 10.0, 6.8 Hz, 1H), 2.75 (s, 3H).

^{13}C NMR (151 MHz, $CDCl_3$) δ 141.4, 136.4, 129.2, 128.6, 128.4, 128.1, 127.8, 126.4, 73.8, 70.6, 52.8, 37.1.

HRMS (ESI): $[M+Na]^+$ calculated for $C_{16}H_{18}NaO_4S^+$ 329.0818; found: 329.0817.

FT-IR (neat): 3536, 3062, 1495, 1454, 1350, 1171, 954, 829, 759, 701 cm^{-1}.

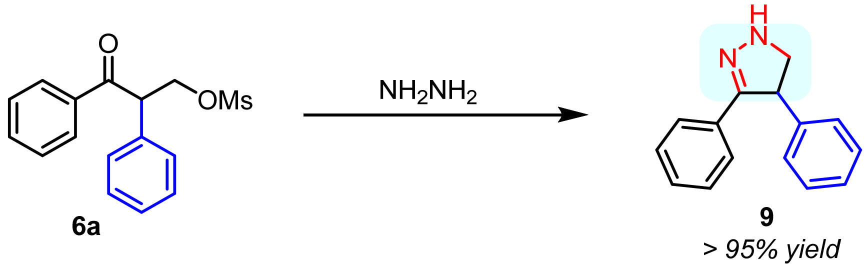

6a **9** *> 95% yield*

NH_2NH_2 solution (1 mL) was added to a C solution of Ketone **6a** (59.0 mg, 0.550 mmol) in EtOH (2.5 mL) in a 10-mL round-bottom flask. Then the flask merge to 100 °C oil bath. The reaction mixture was stirred at same temperature for 12h. After that, the EtOH was removed under vacuum. Then *sat.* $NaHCO_3$ was added. The resulting solution was extracted with EtOAc (3×15 mL), and the combined organic layers were dried over MgSO4, filtered, and concentrated, which afforded the product as a white solid in quantitative yield without further purification.

^{1}H NMR (600 MHz, $CDCl_3$) δ 7.62 – 7.56 (m, 2H), 7.34 – 7.15 (m, 8H), 4.52 (dt, *J* = 7.6, 5.5 Hz, 1H), 3.97 – 3.93 (m, 1H), 3.58 – 3.46 (m, 1H).

^{13}C NMR (151 MHz, $CDCl_3$) δ 154.4, 140.6, 132.1, 128.9, 128.5, 128.4, 127.6, 127.0, 126.5, 57.5, 51.3.

HRMS (ESI): $[M+Na]^+$ calculated for $C_{15}H_{15}N_2$ 223.1230; found: 223.1226.

FT-IR (neat): 3340, 3028, 1680, 1600, 1493, 1447, 1220, 773, 696 cm^{-1}.

7. Spectra

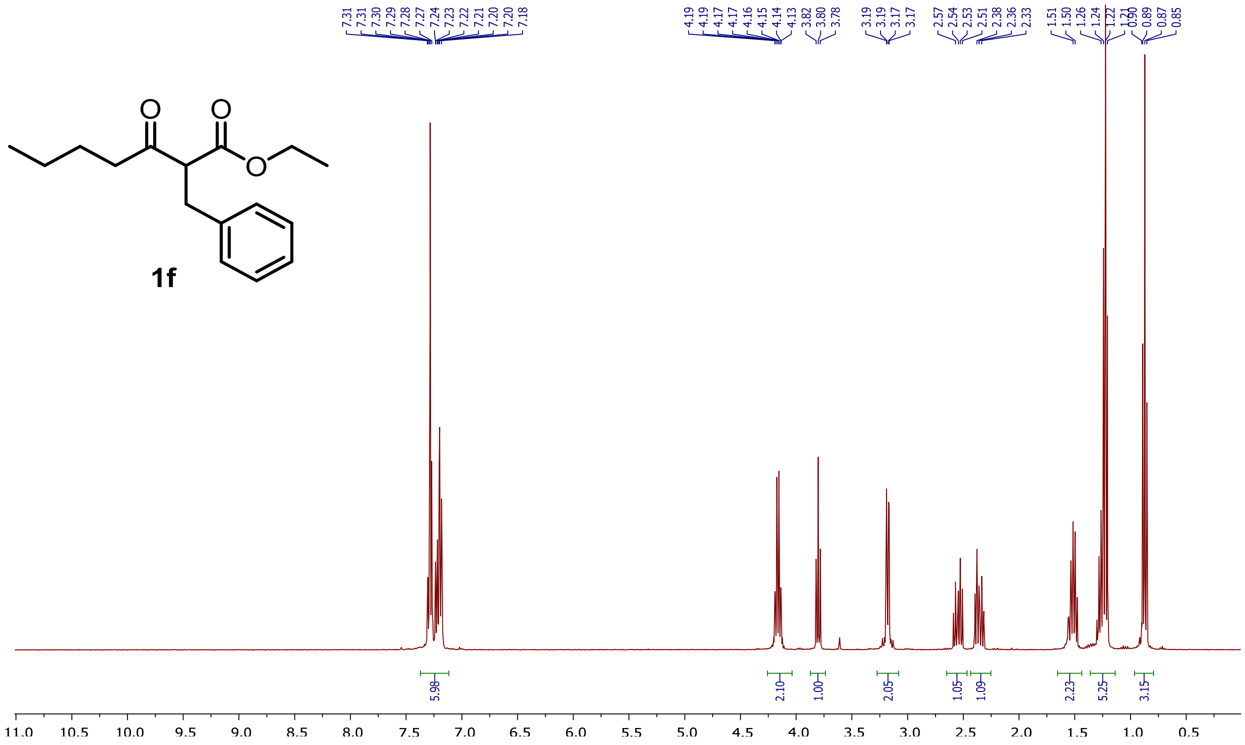

^{1}H NMR spectra of **1f**

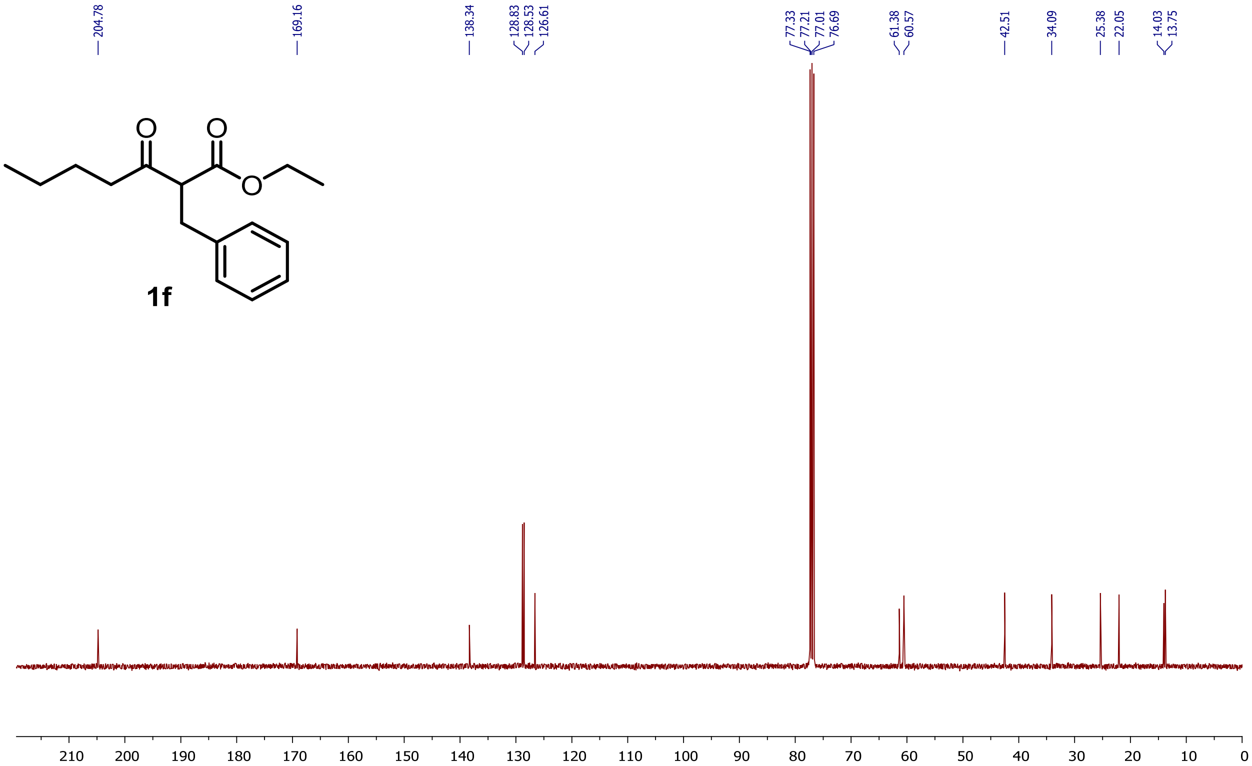

^{13}C NMR spectra of **1f**

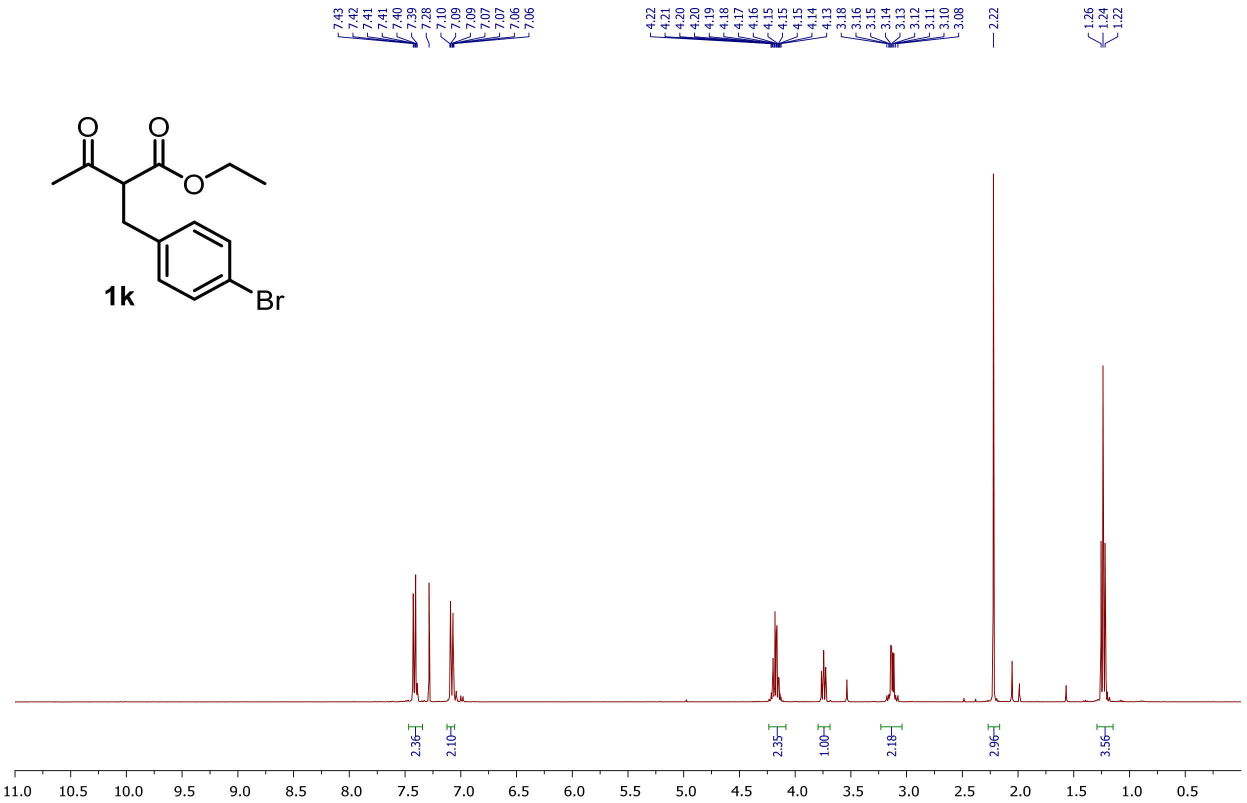

^{1}H NMR spectra of **1k**

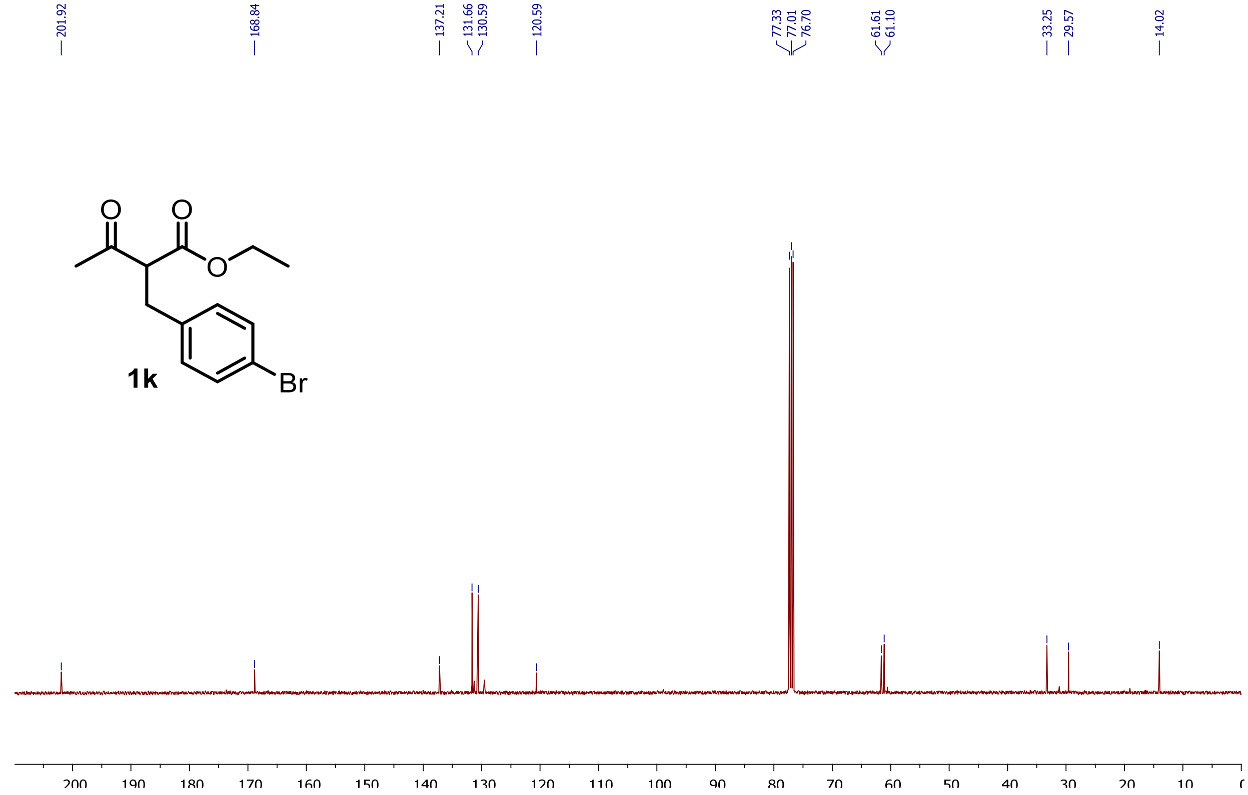

^{13}C NMR spectra of **1k**

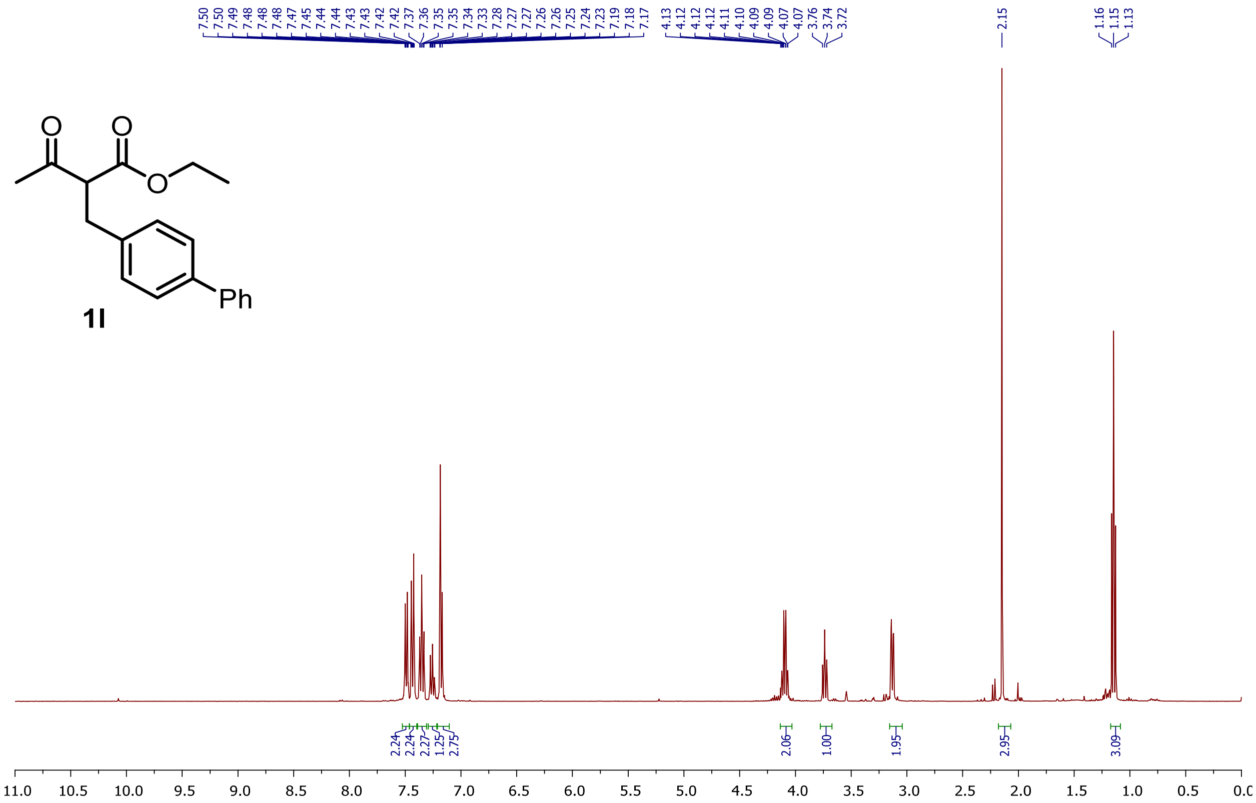

^{1}H NMR spectra of **1l**

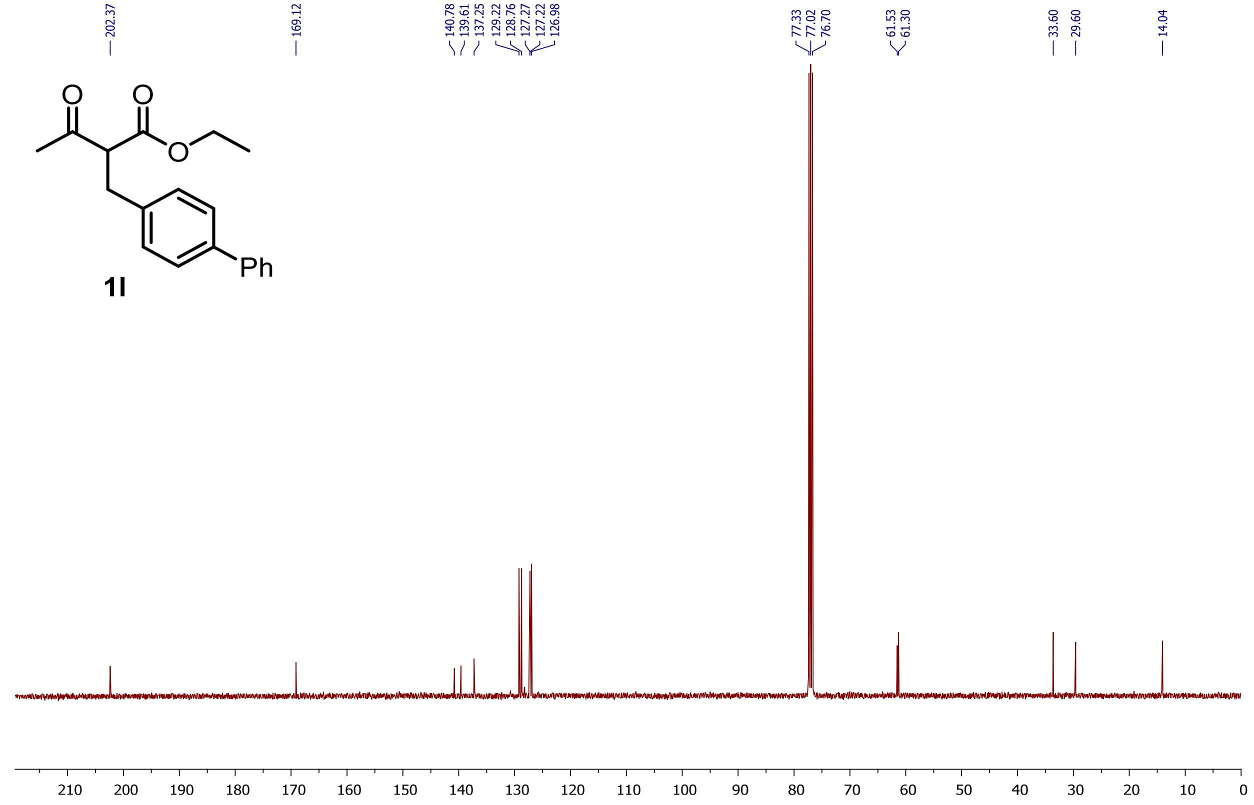

^{13}C NMR spectra of **1l**

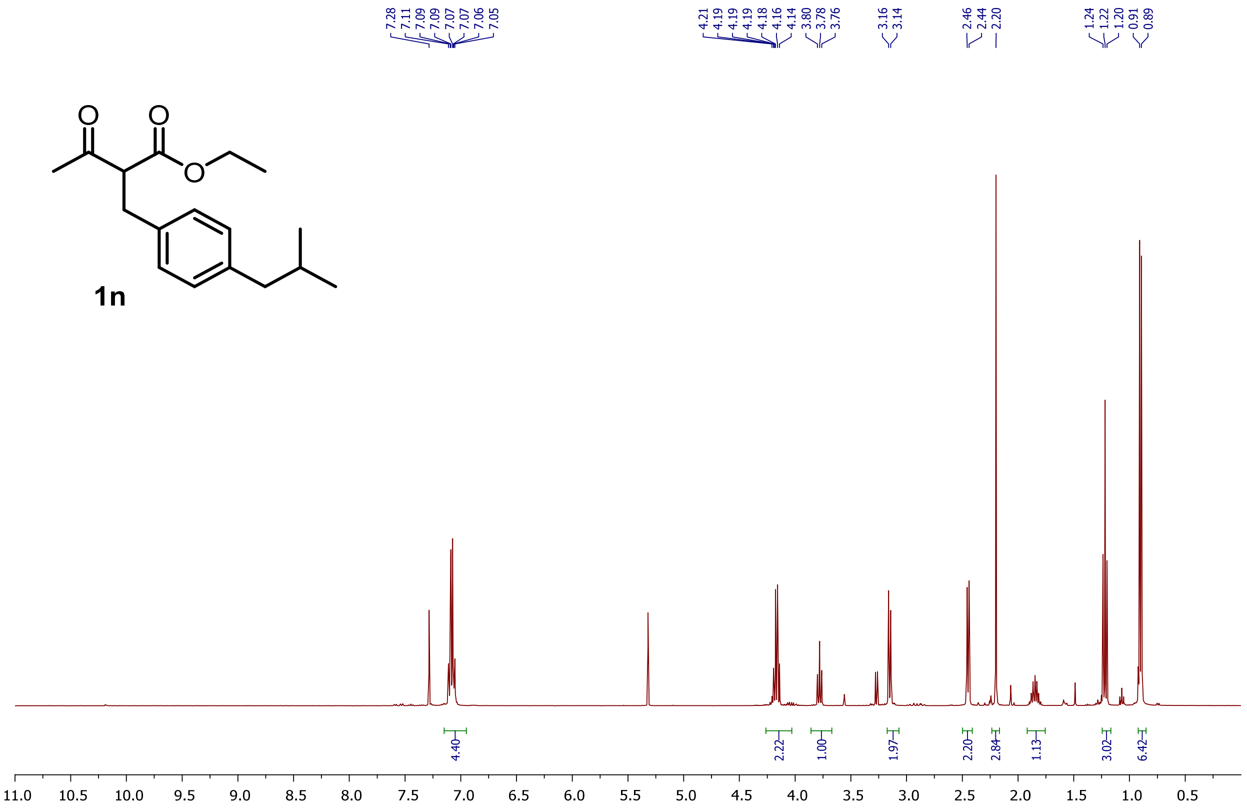

^{1}H NMR spectra of **1n**

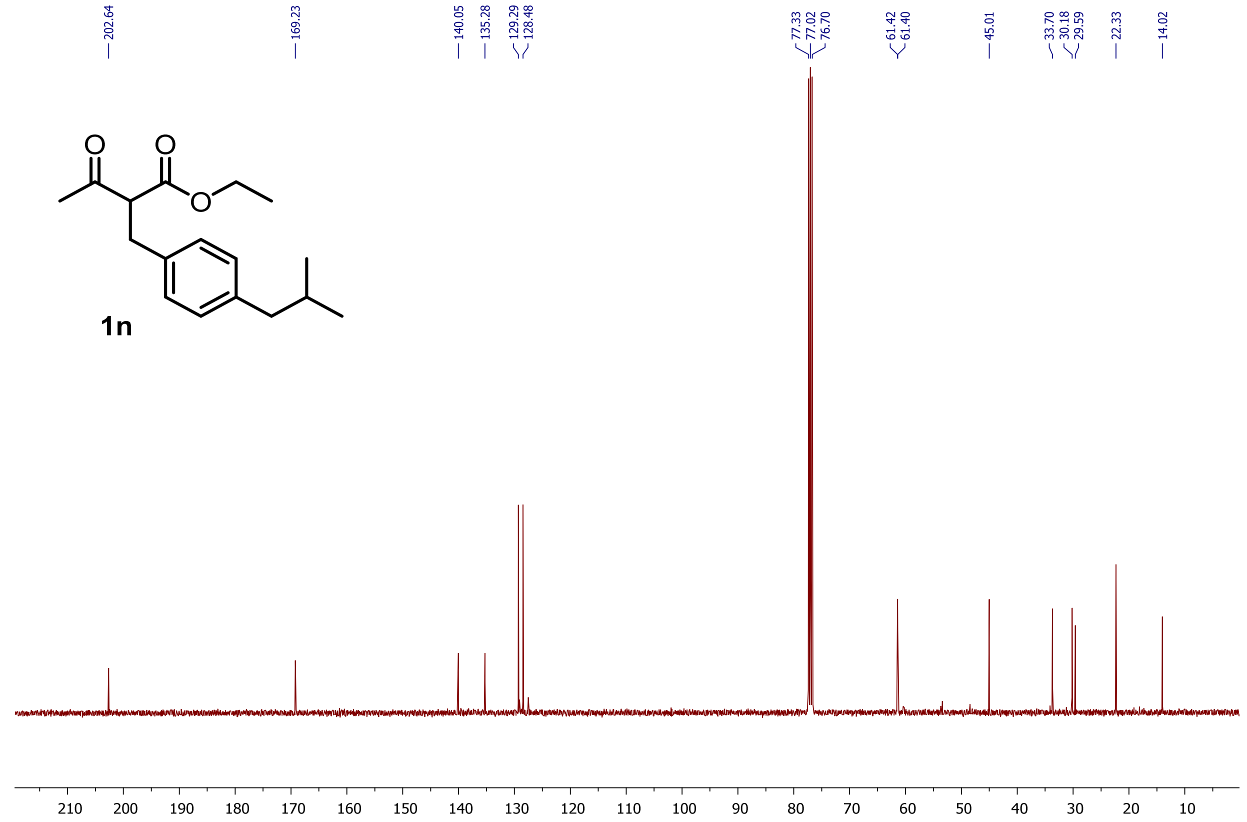

^{13}C NMR spectra of **1n**

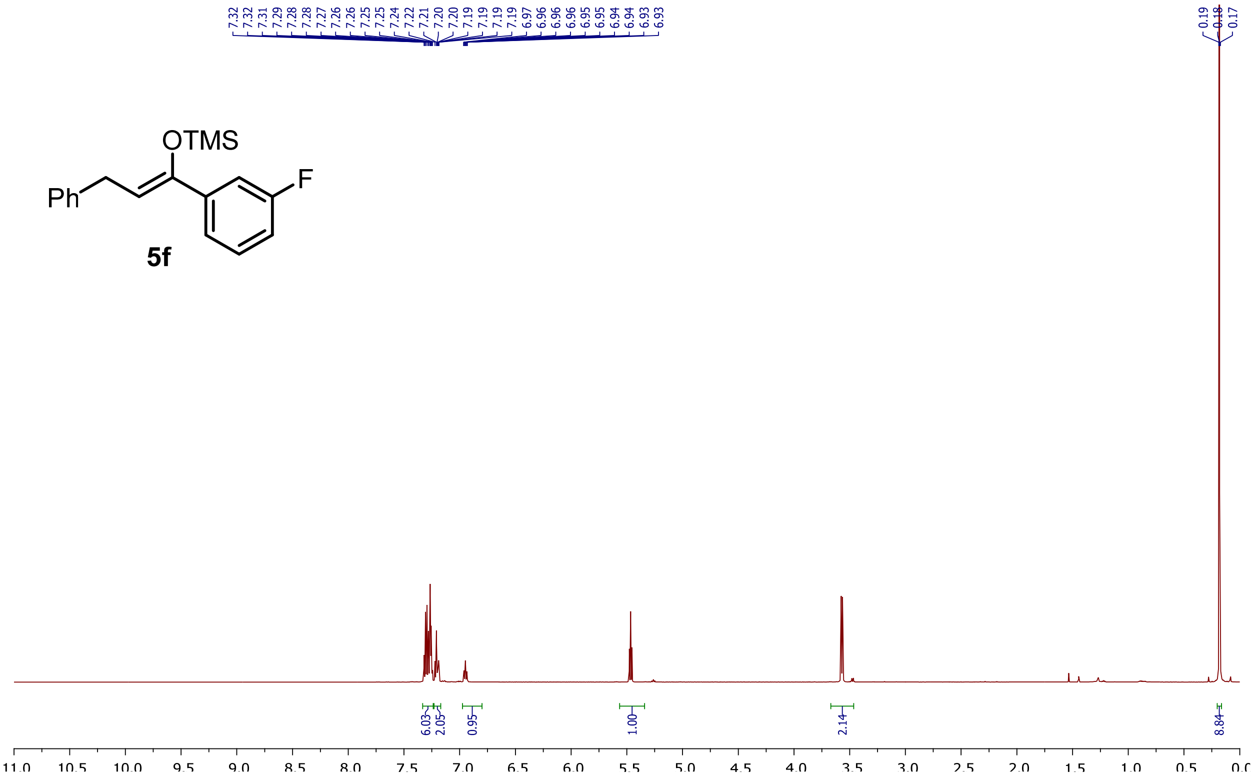

^{1}H NMR spectra of **5f**

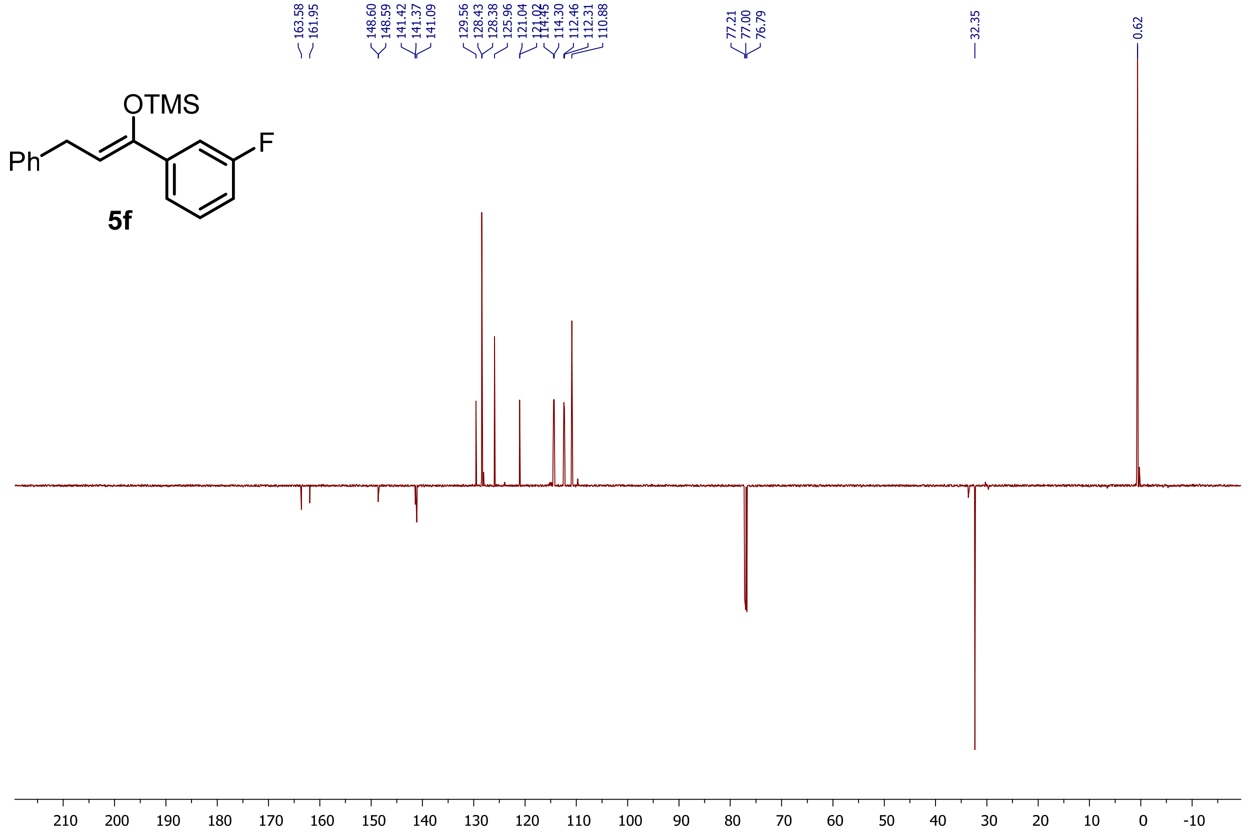

^{13}C NMR spectra of **5f**

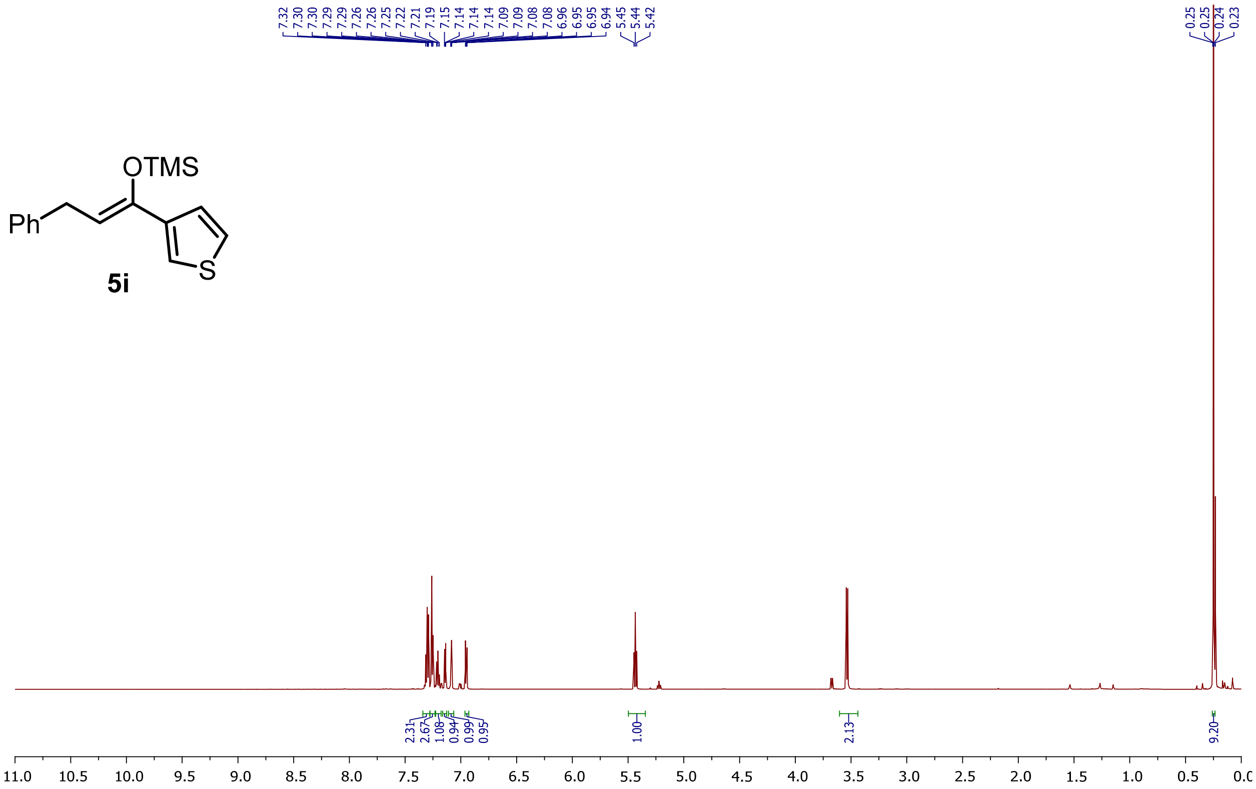

^{1}H NMR spectra of **5i**

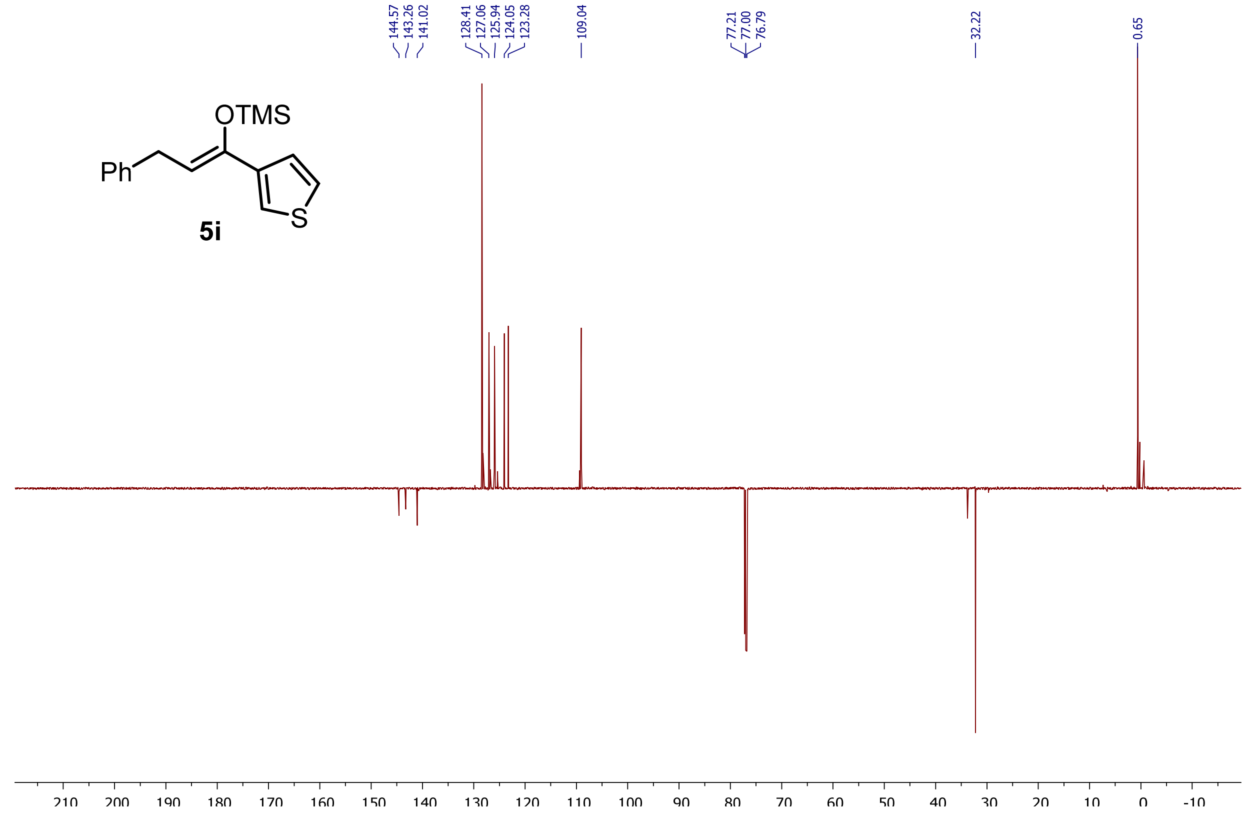

^{13}C NMR spectra of **5i**

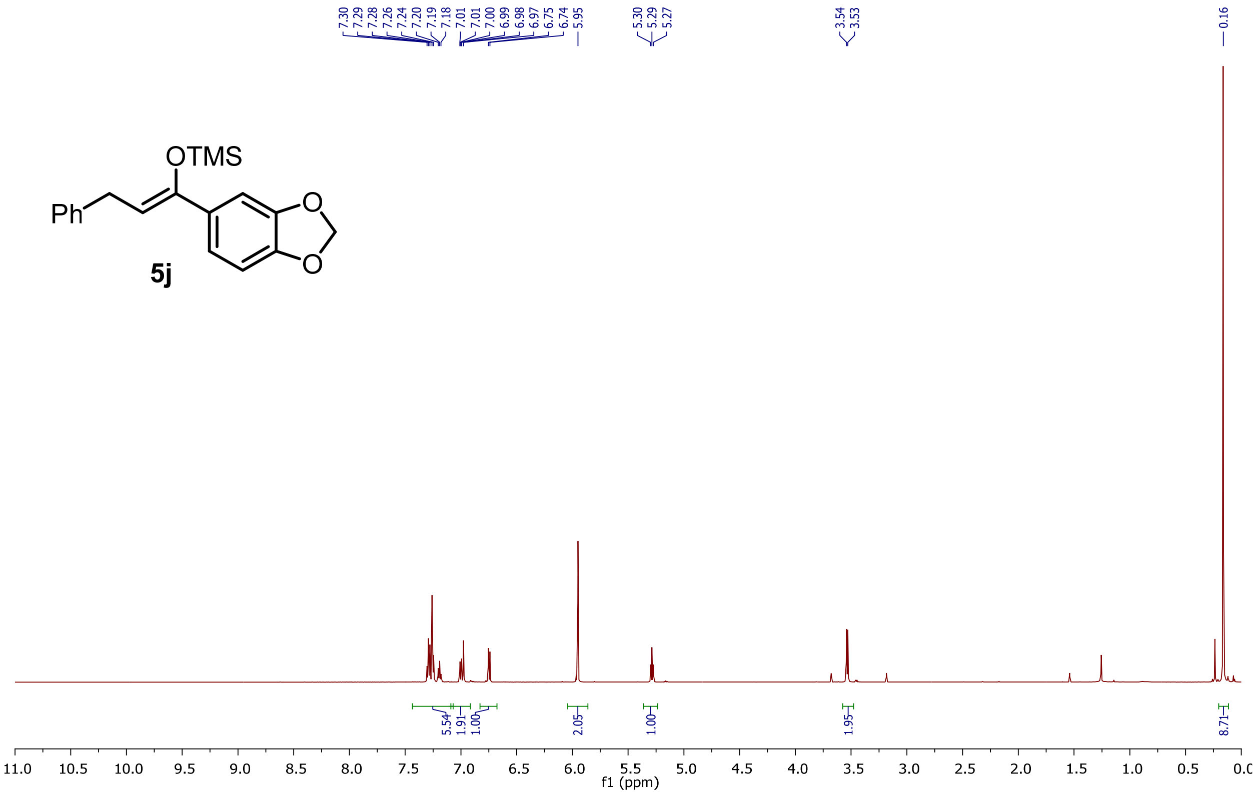

^{1}H NMR spectra of **5j**

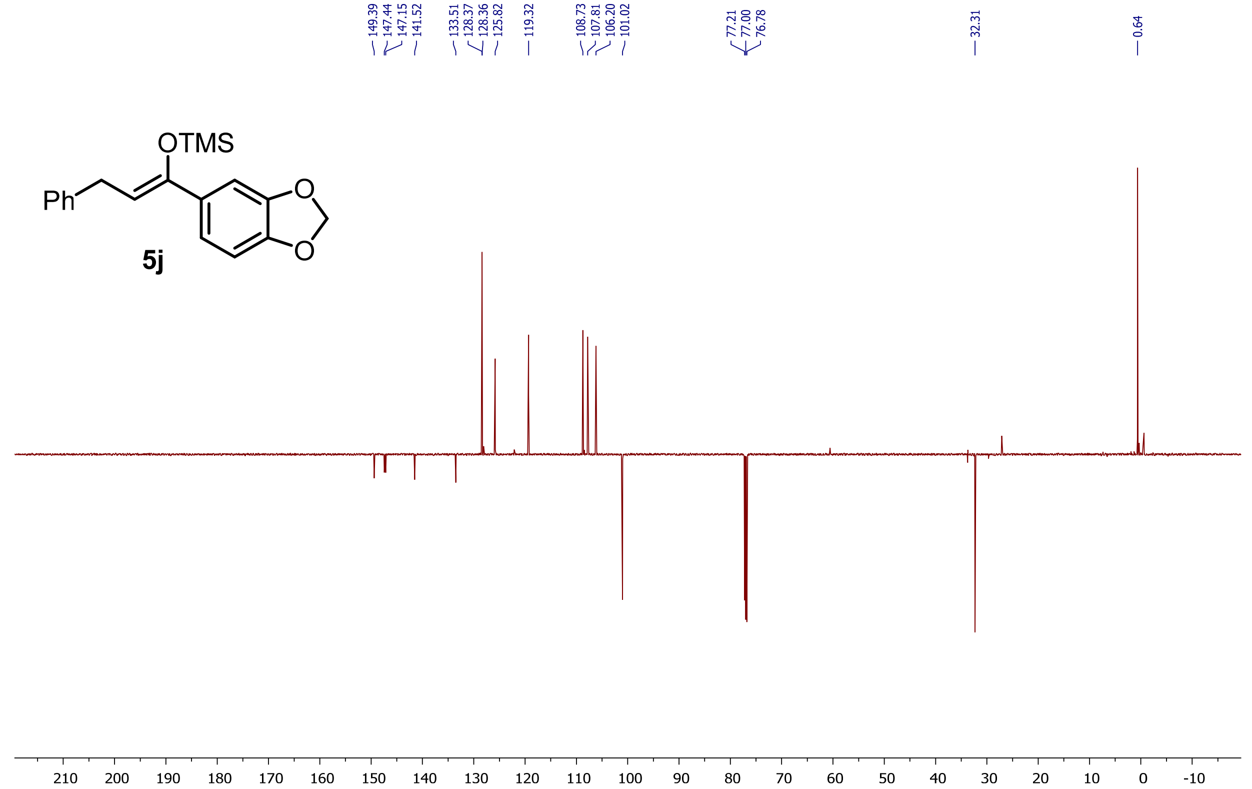

^{13}C NMR spectra of **5j**

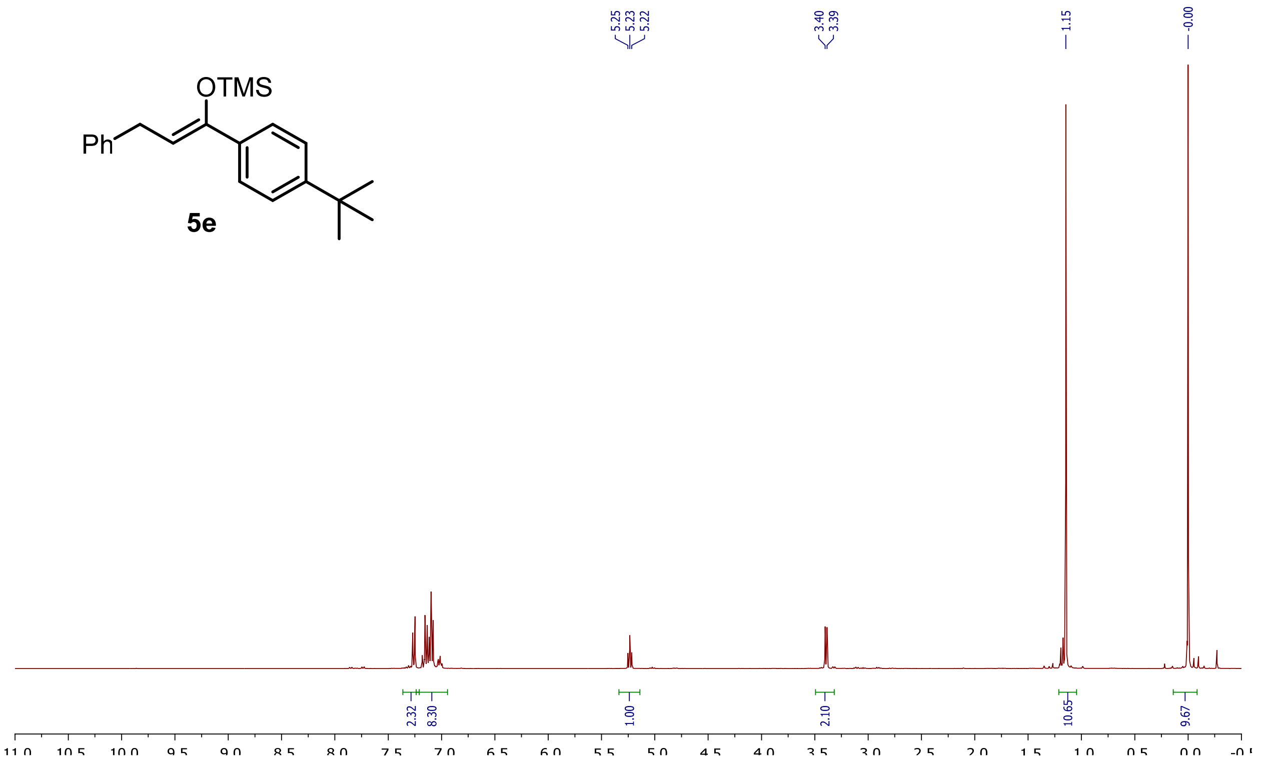

^{1}H NMR spectra of **5e**

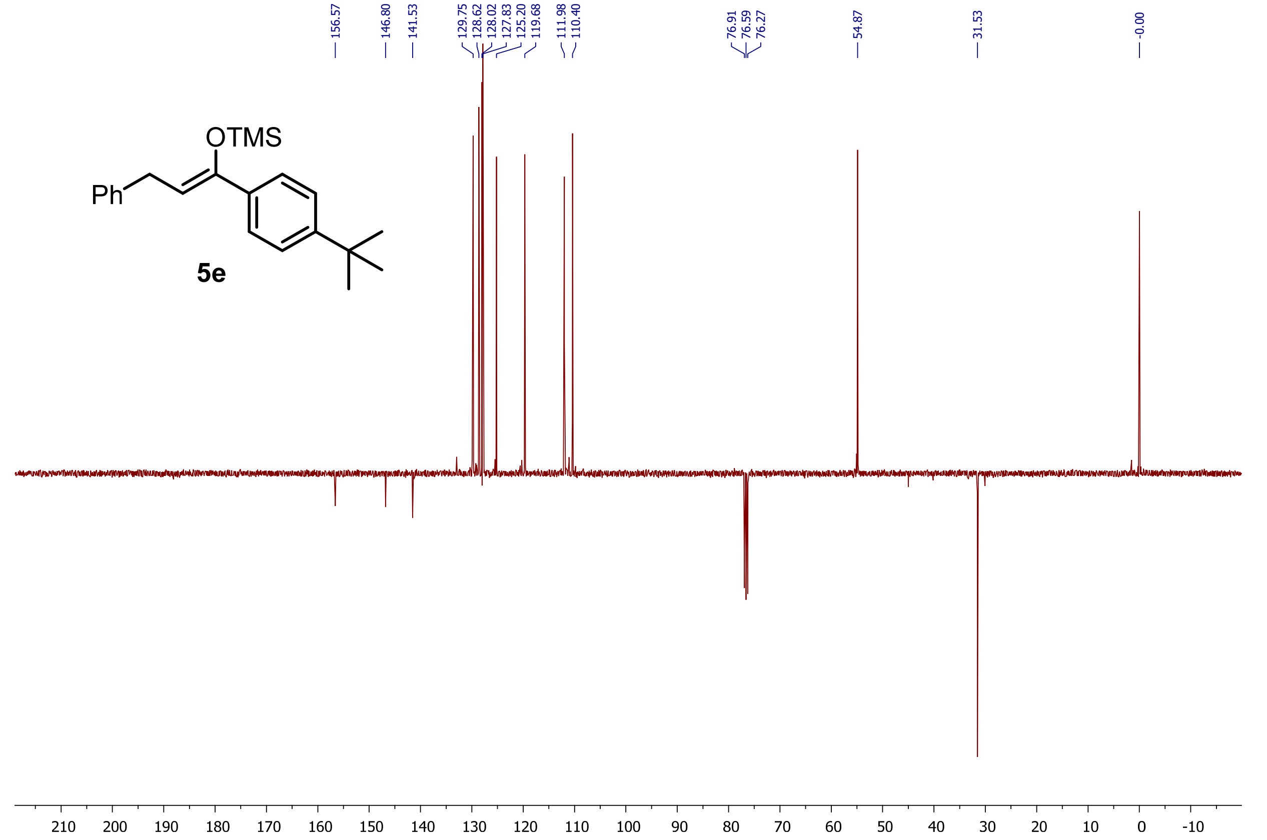

^{13}C NMR spectra of **5e**

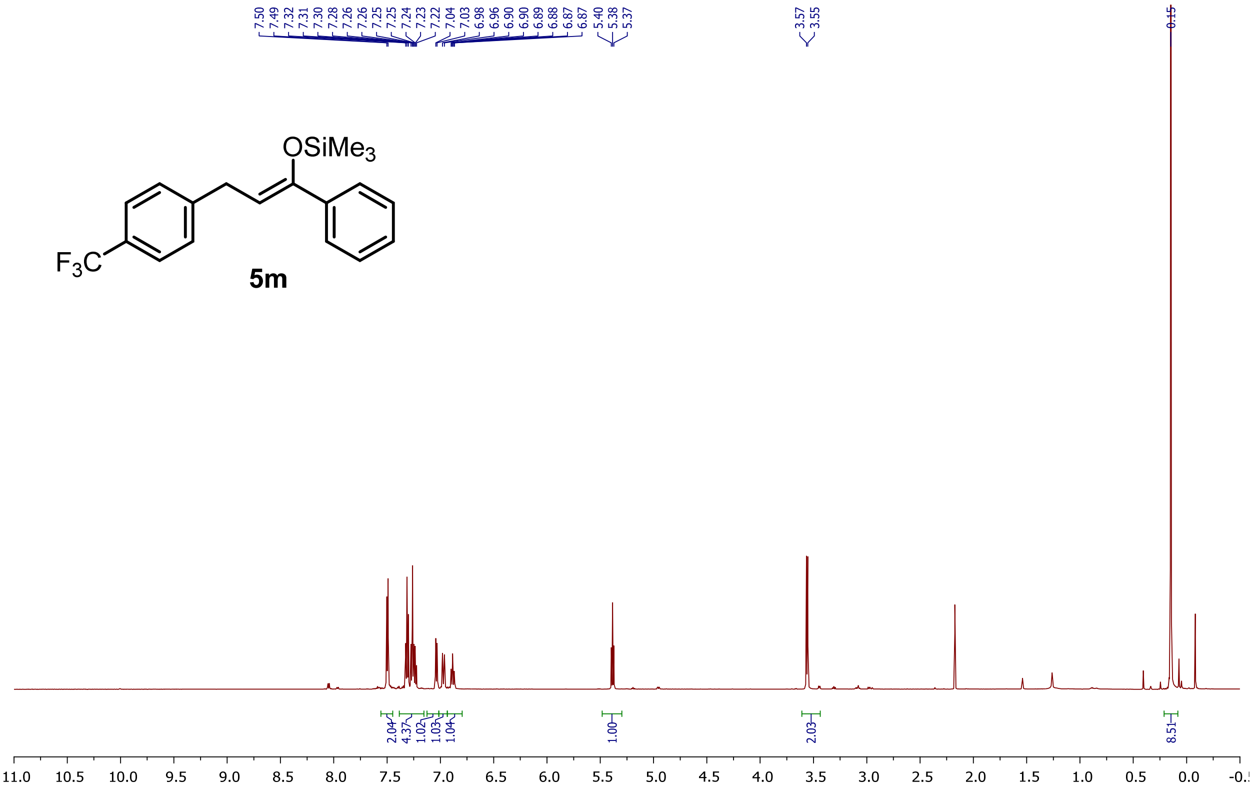

^{1}H NMR spectra of **5m**

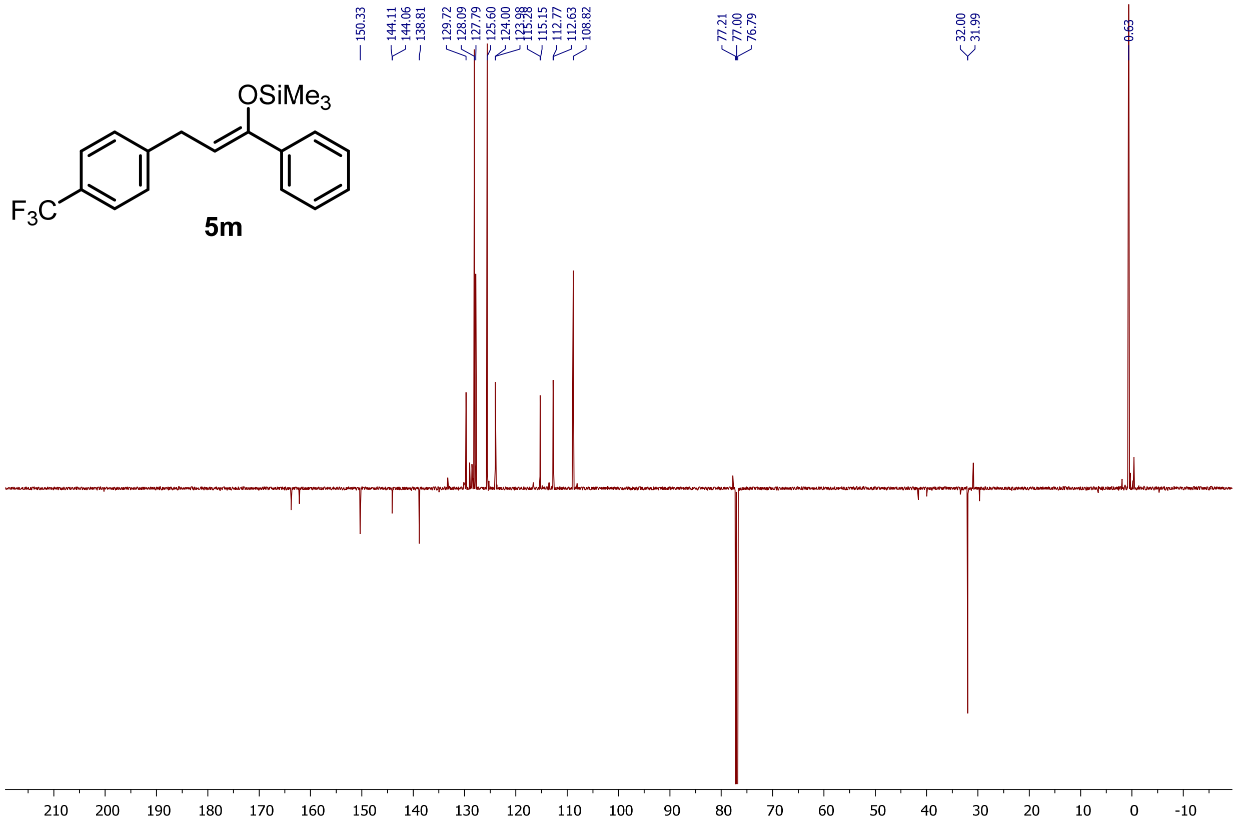

^{13}C NMR spectra of **5m**

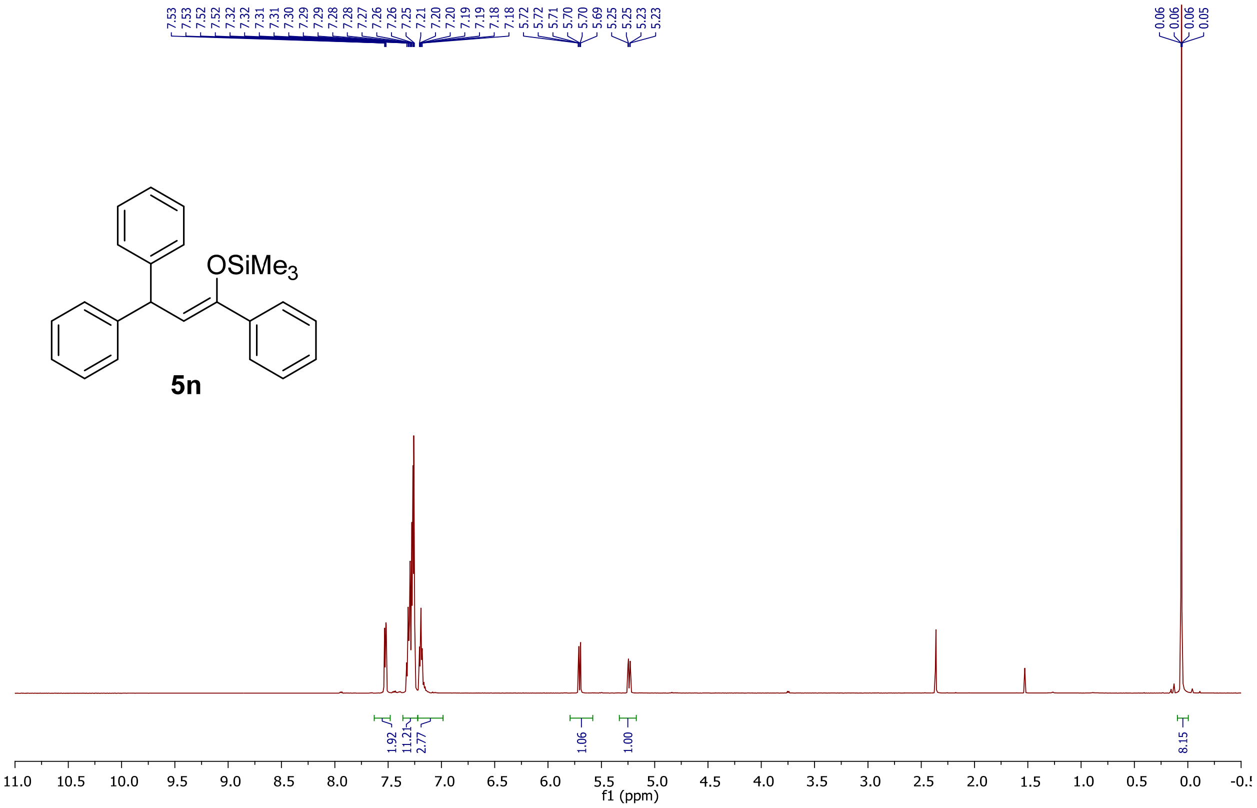

^{1}H NMR spectra of **5n**

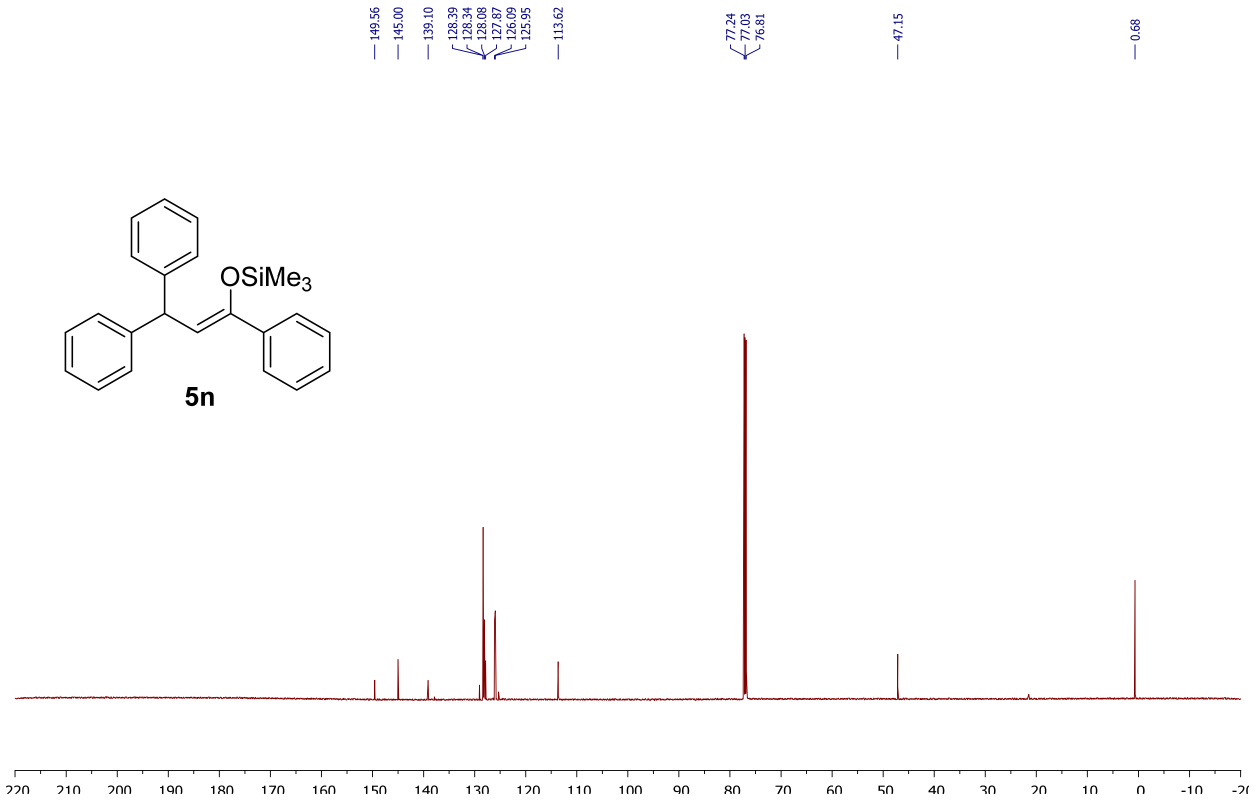

^{13}C NMR spectra of **5n**

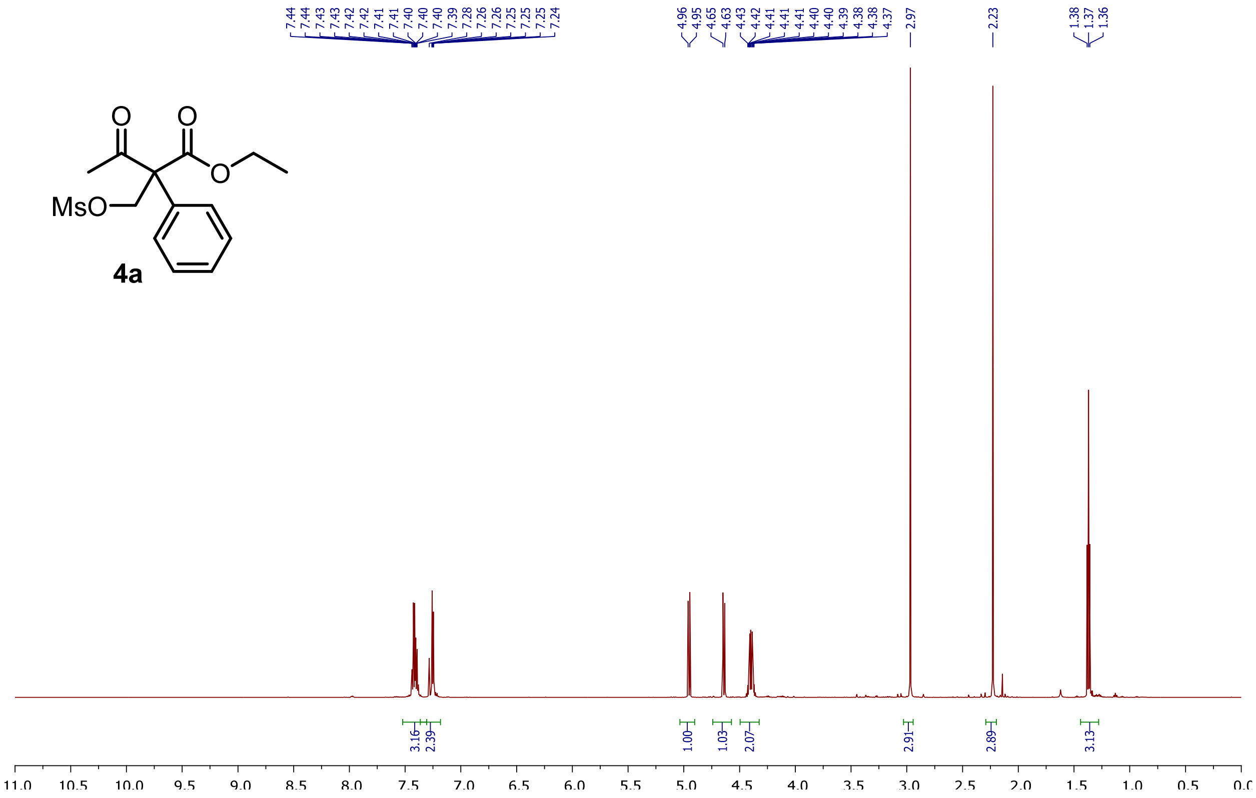

^{1}H NMR spectra of **4a**

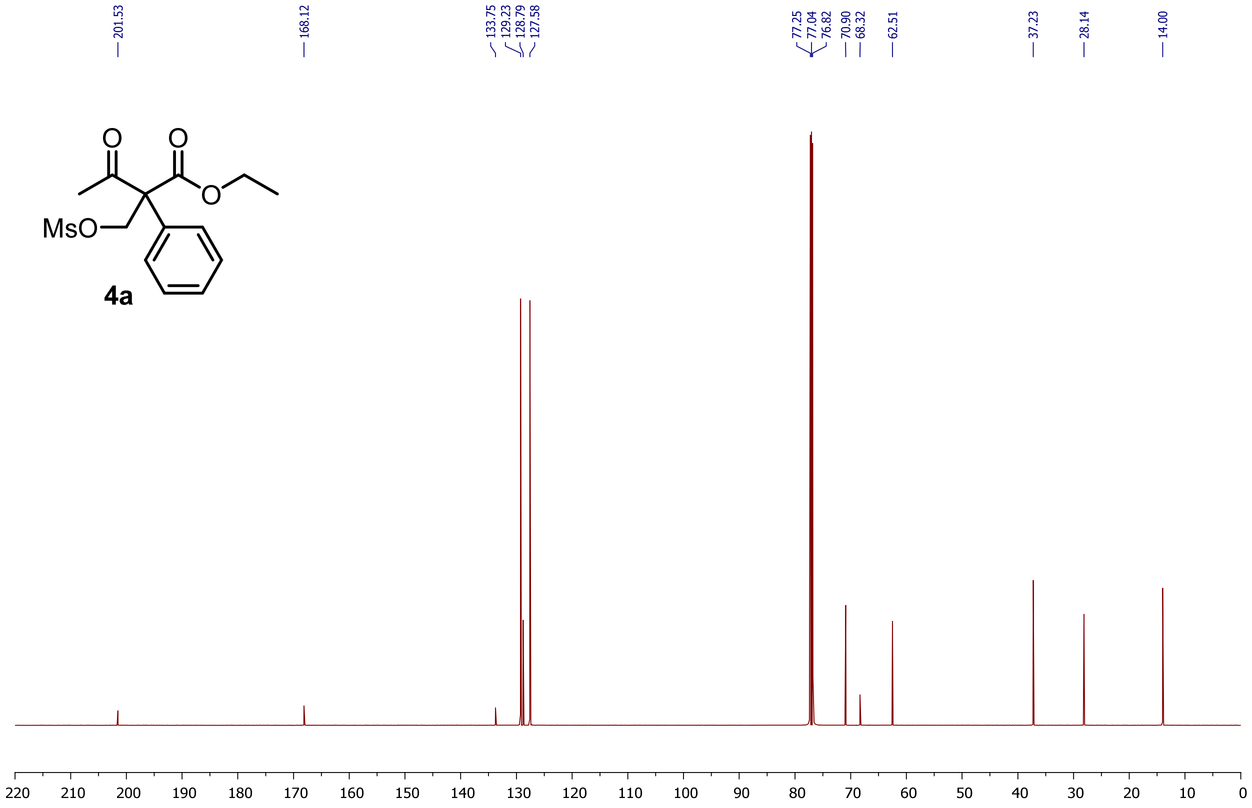

^{13}C NMR spectra of **4a**

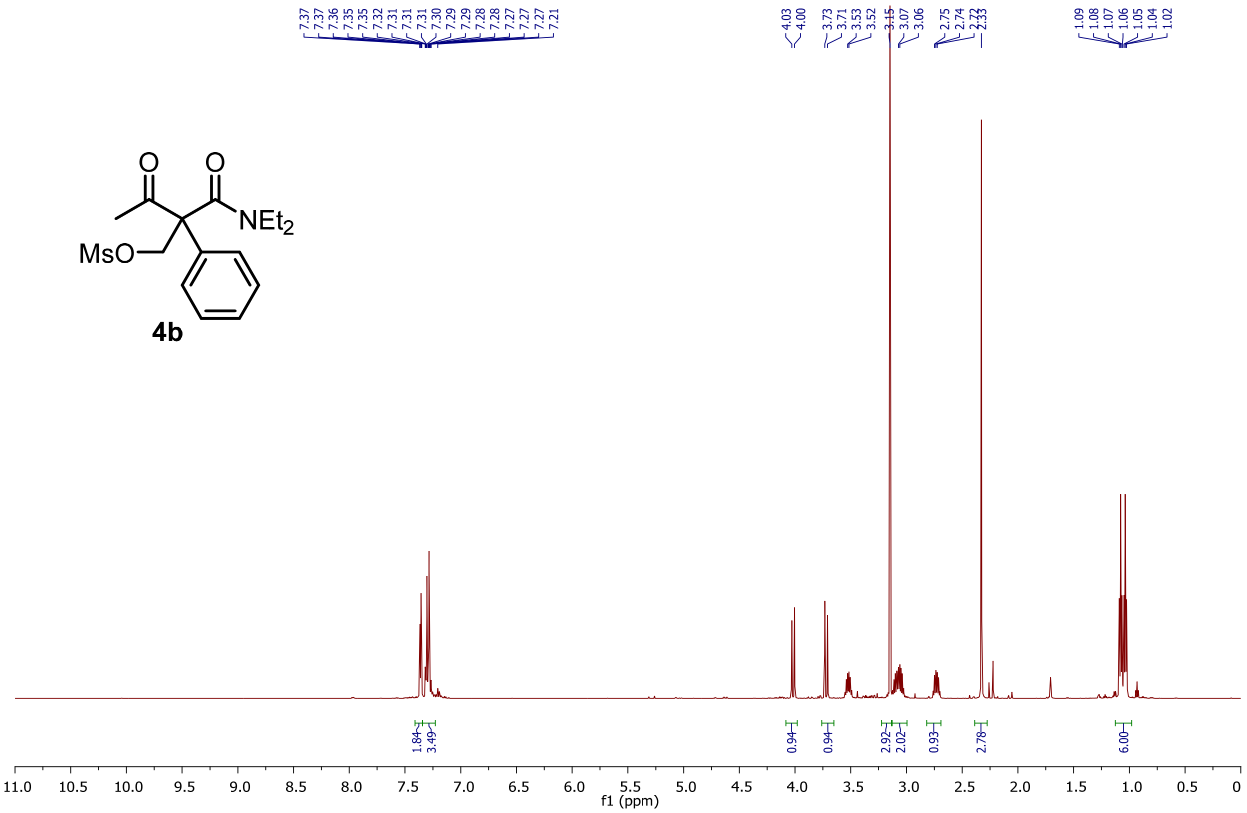

^{1}H NMR spectra of **4b**

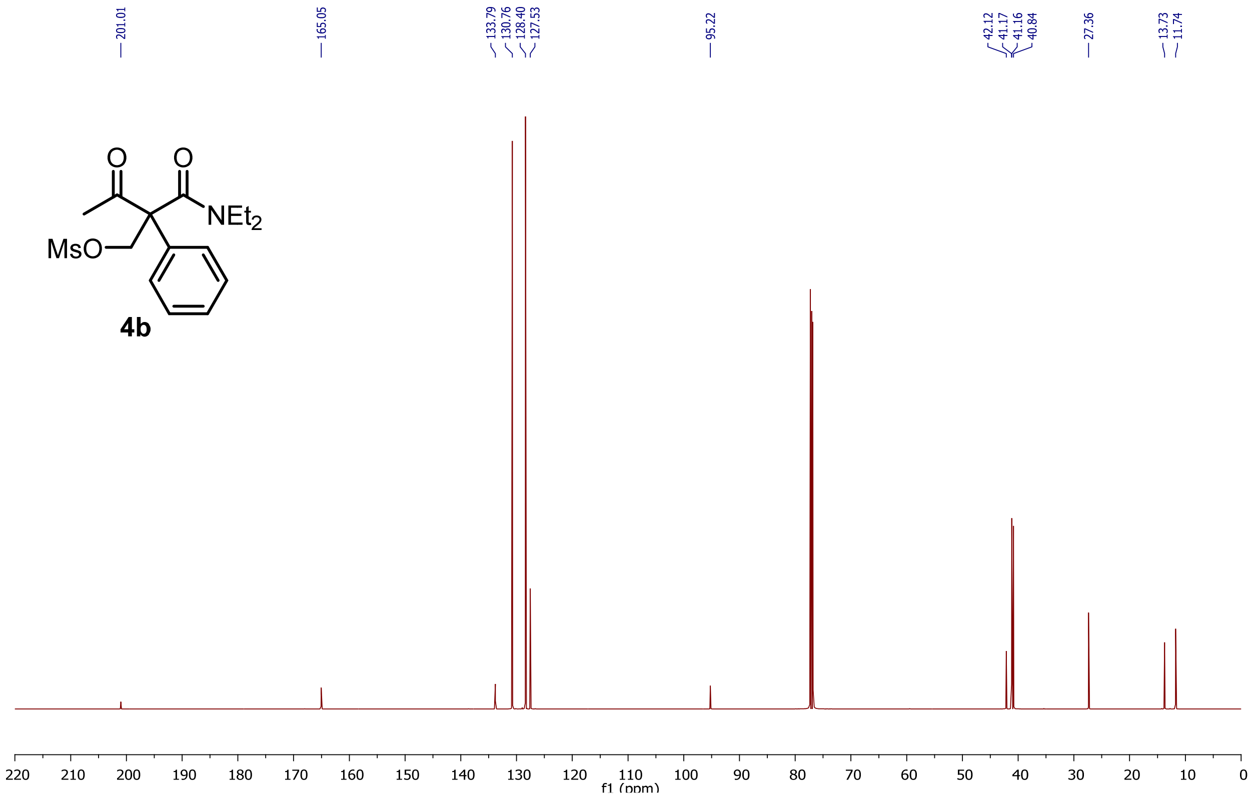

^{13}C NMR spectra of **4b**

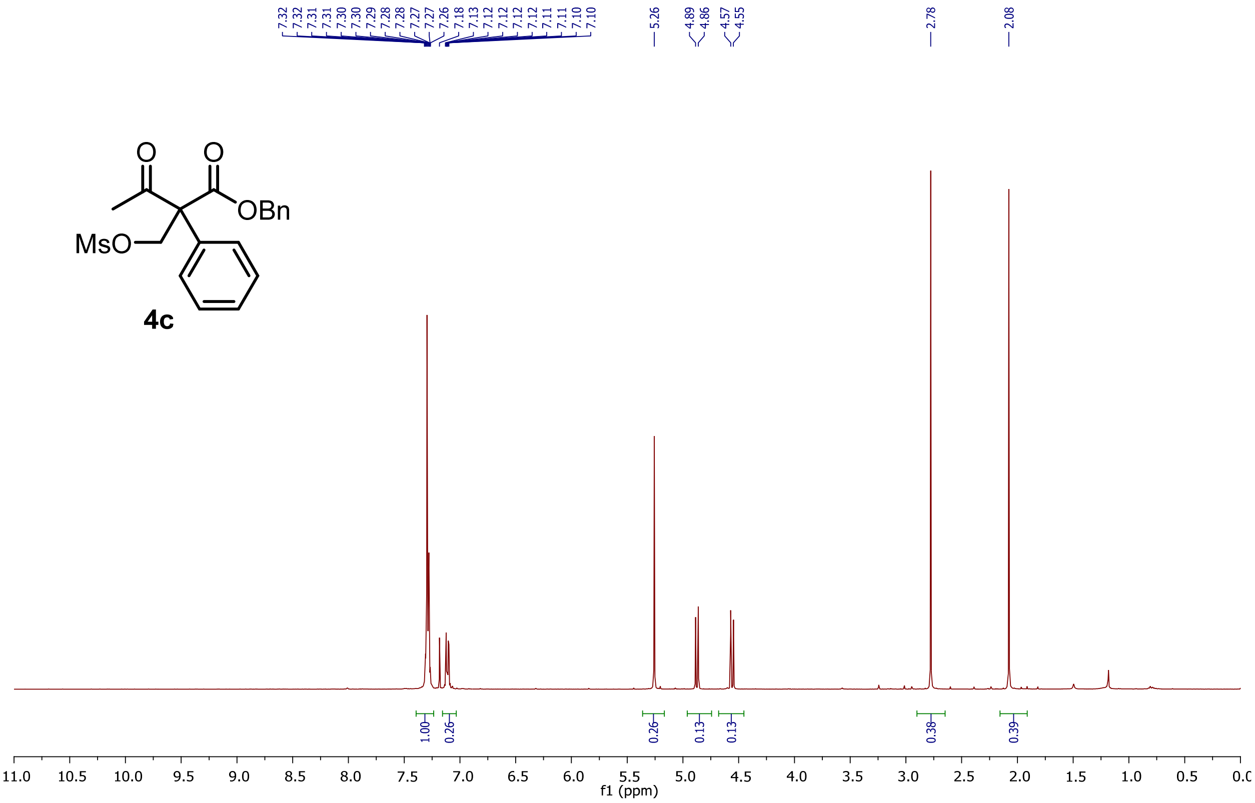

^{1}H NMR spectra of **4c**

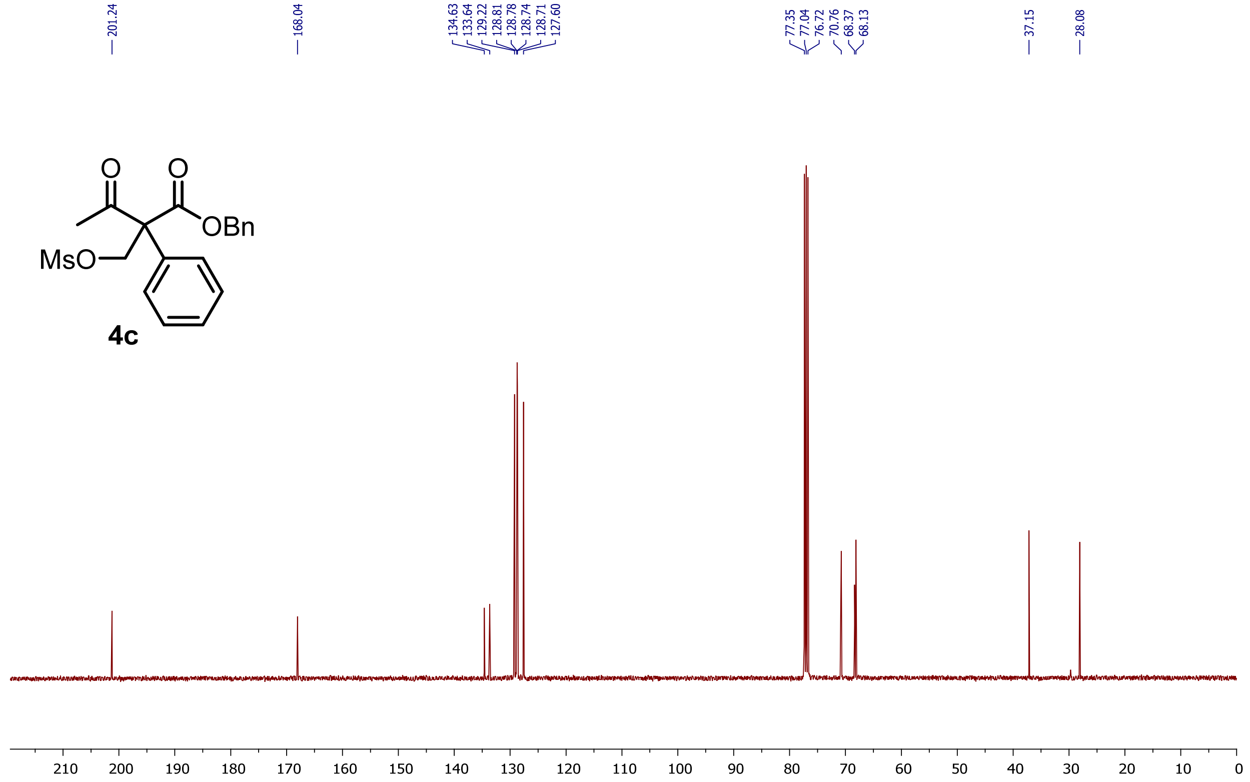

^{13}C NMR spectra of **4c**

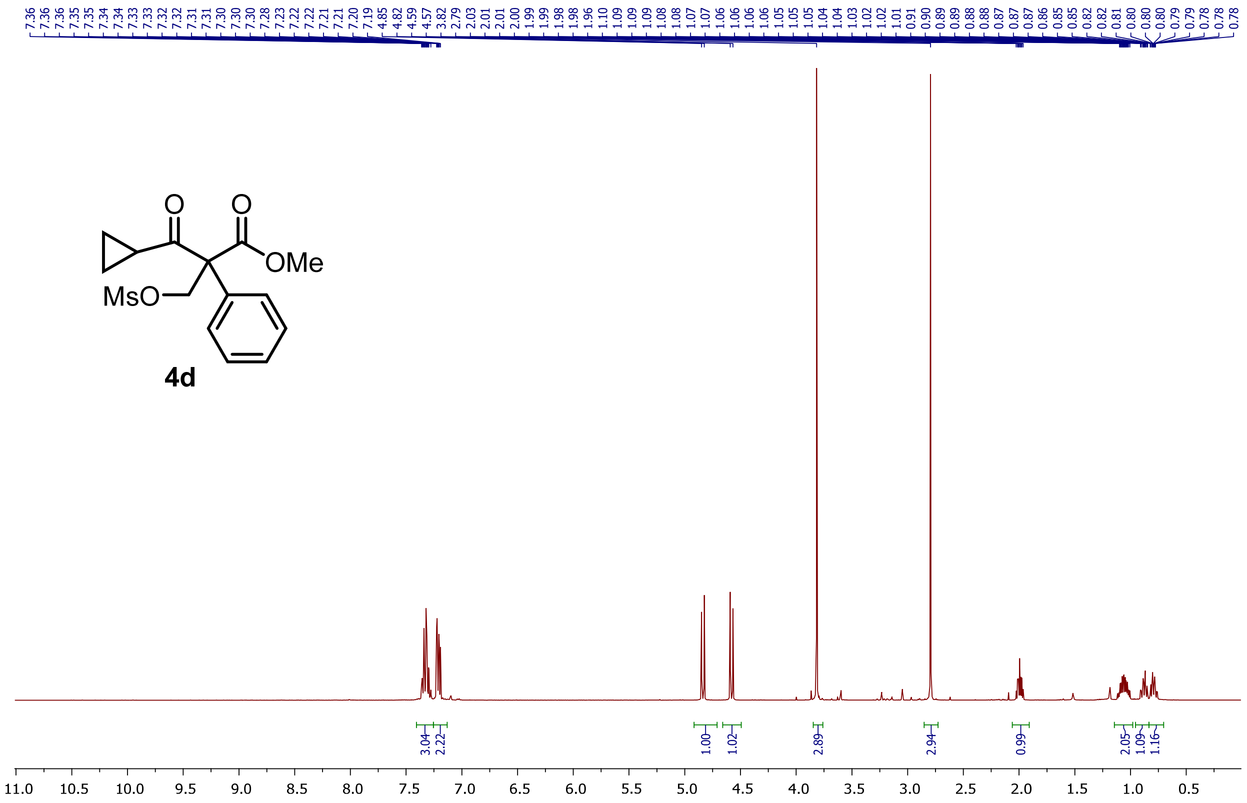

^{1}H NMR spectra of **4d**

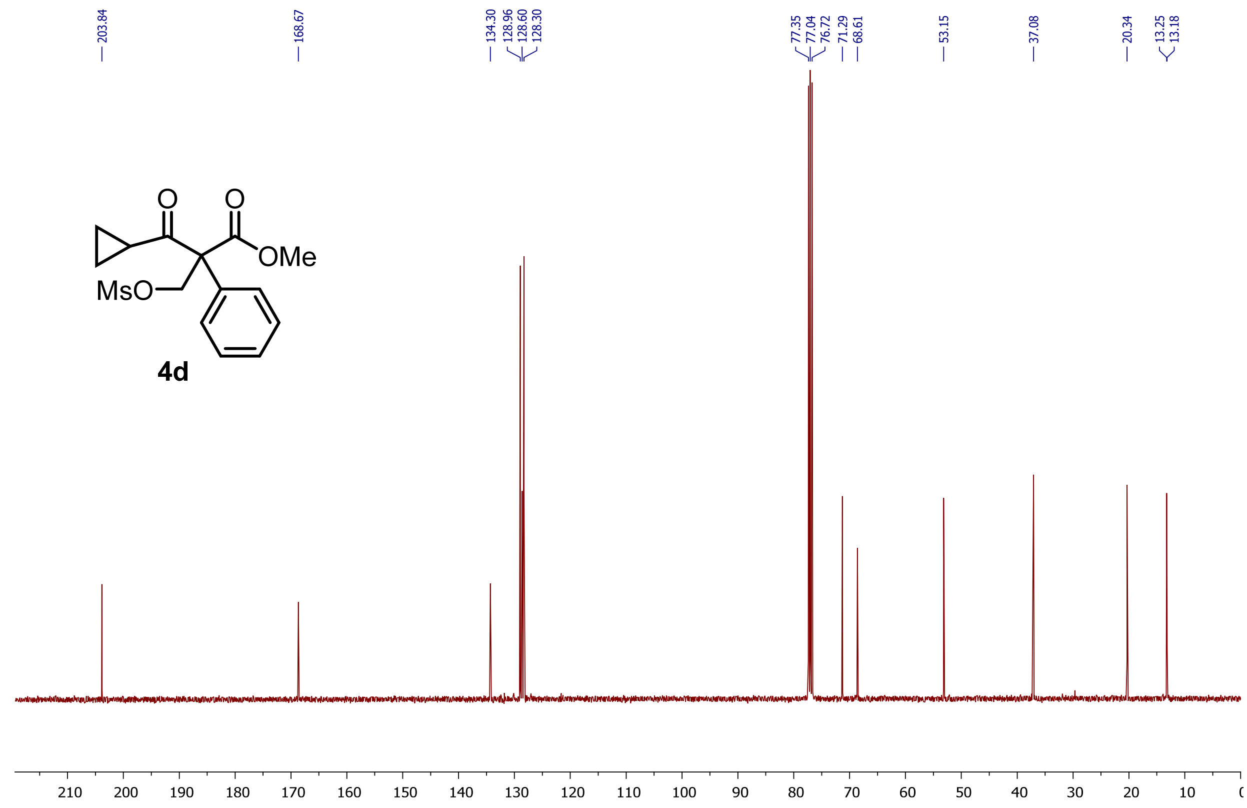

^{13}C NMR spectra of **4d**

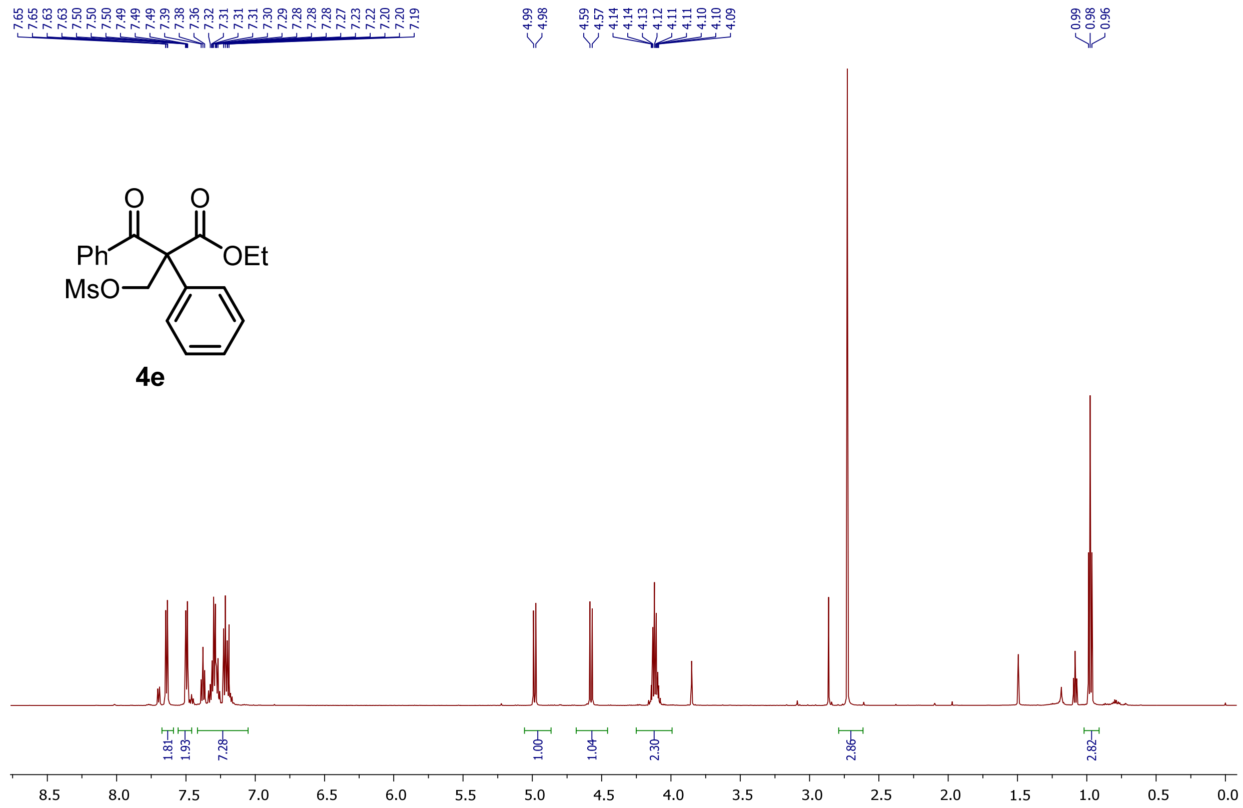

4e

^{1}H NMR spectra of **4e**

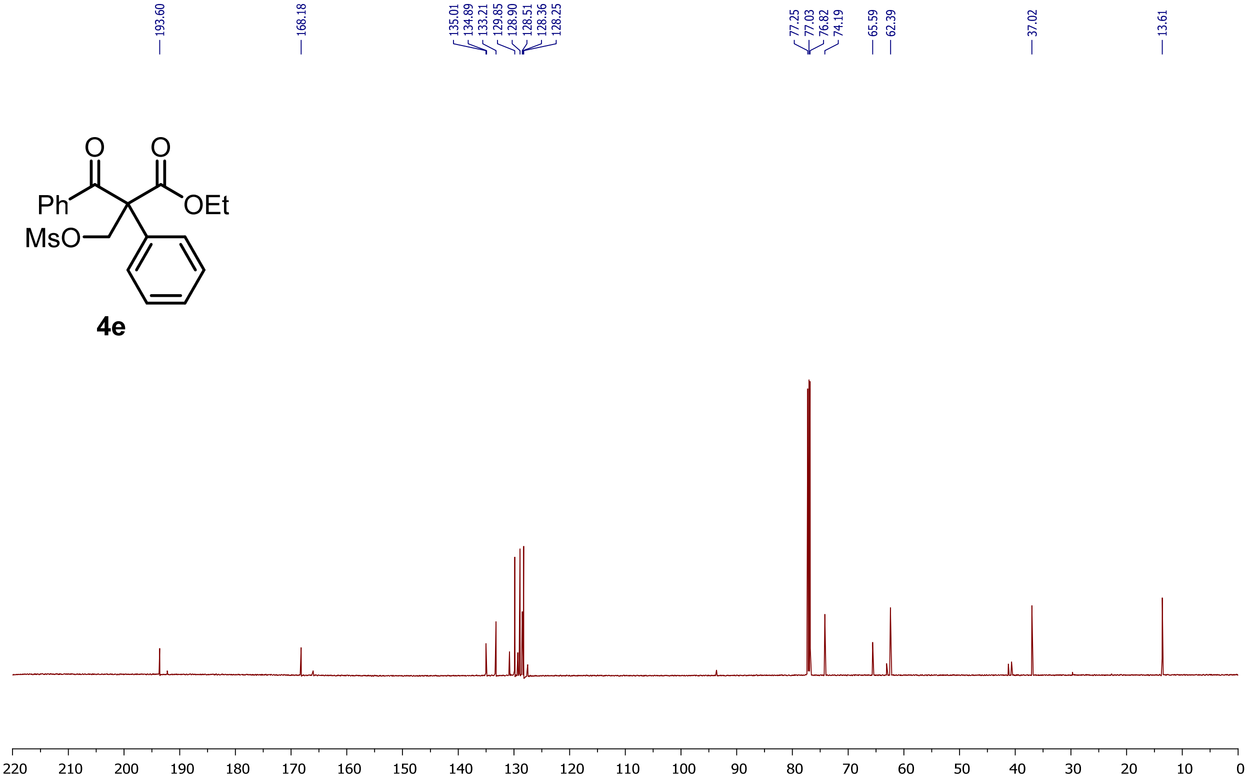

4e

^{13}C NMR spectra of **4e**

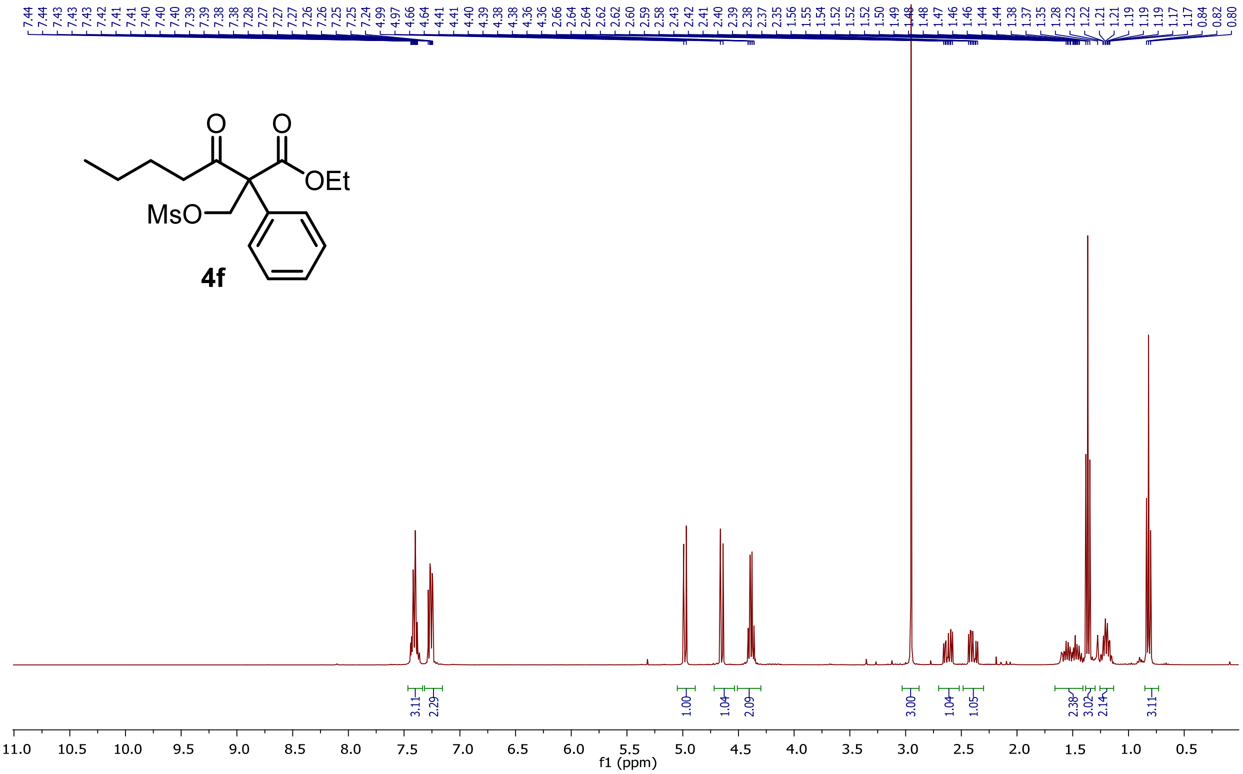

^{1}H NMR spectra of **4f**

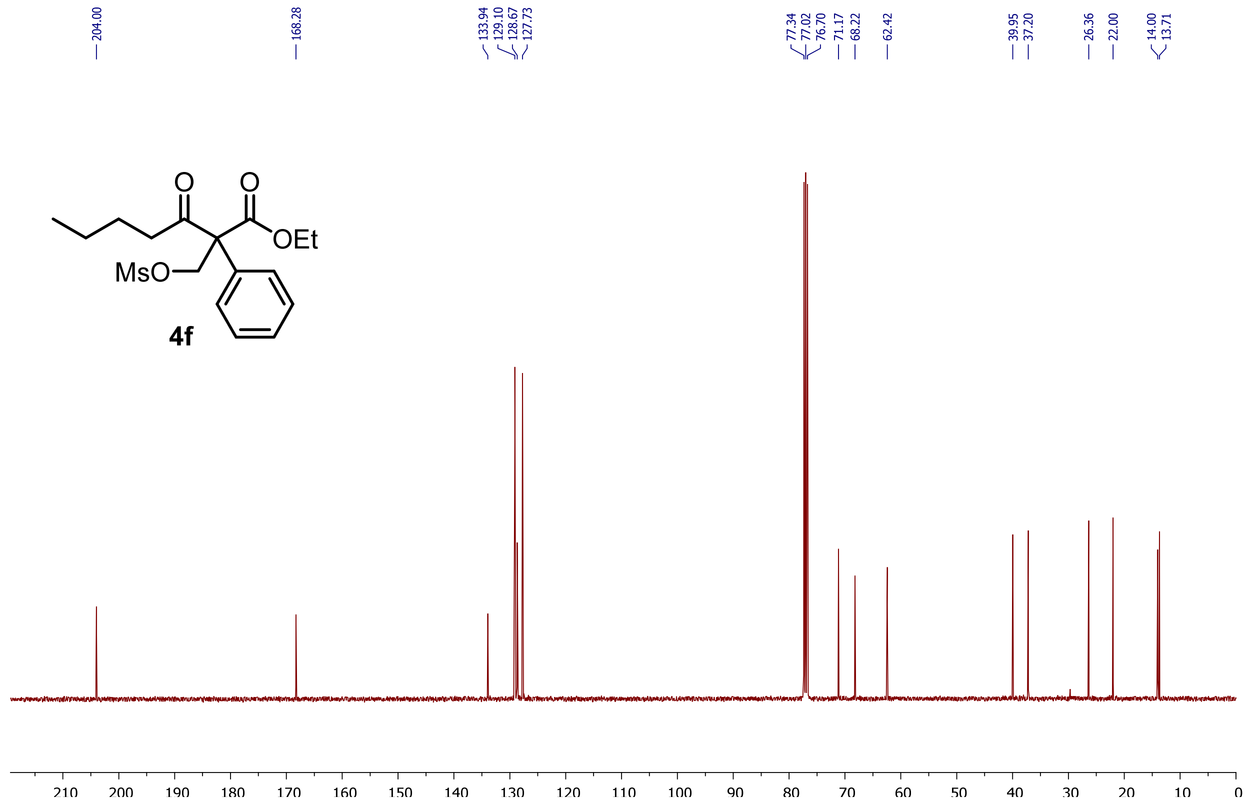

^{13}C NMR spectra of **4f**

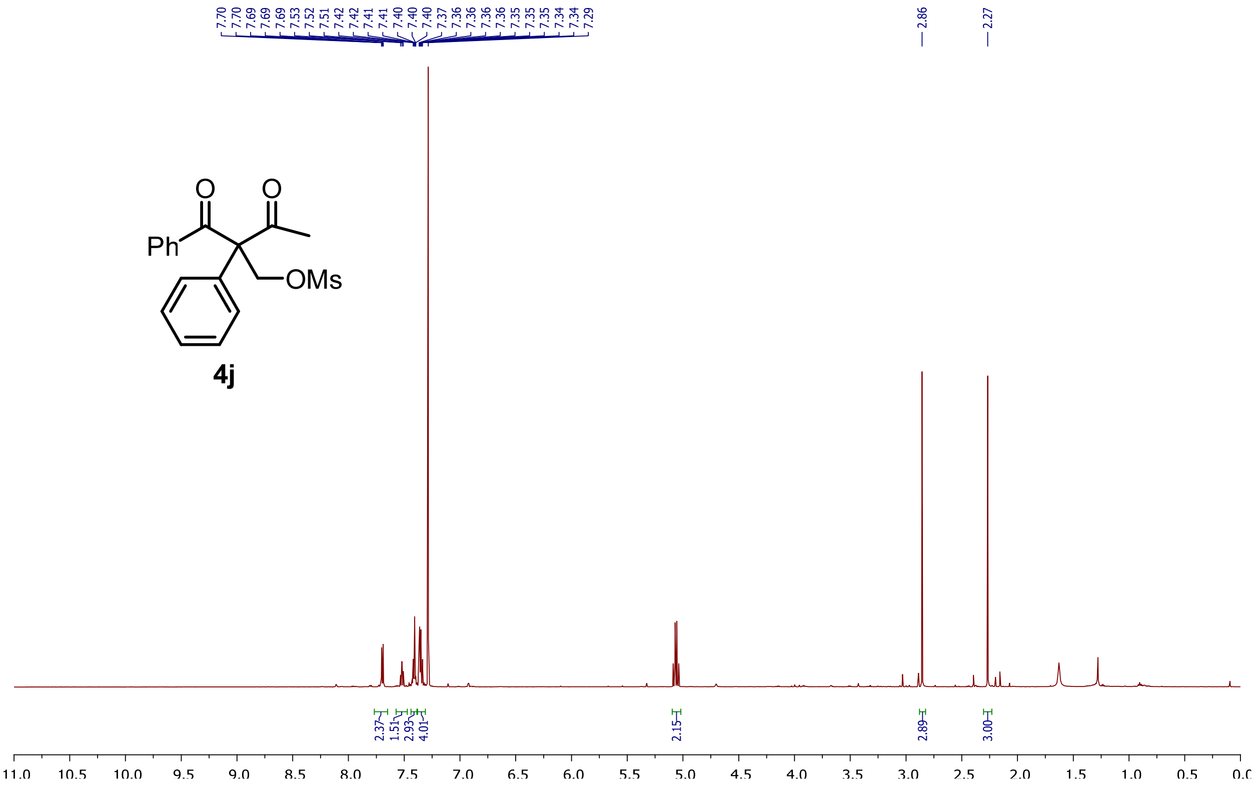

^{1}H NMR spectra of **4j**

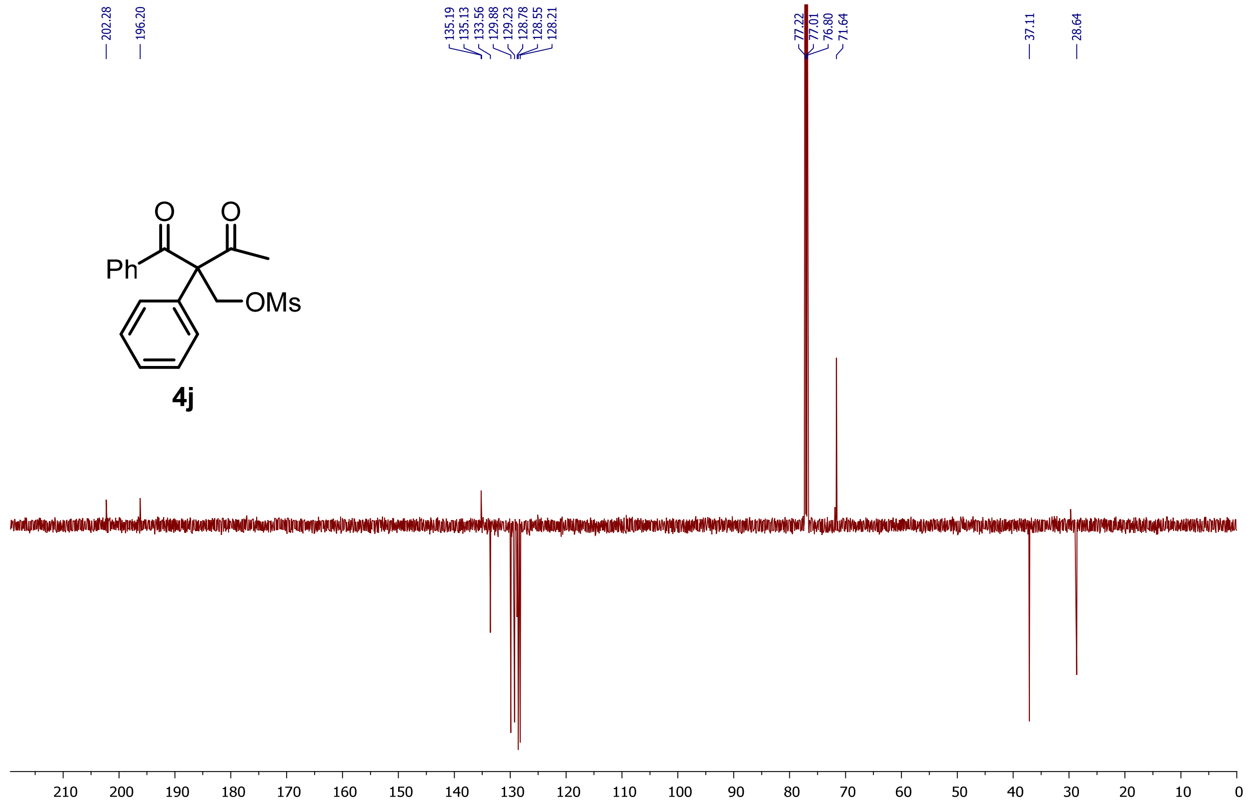

^{13}C NMR spectra of **4j**

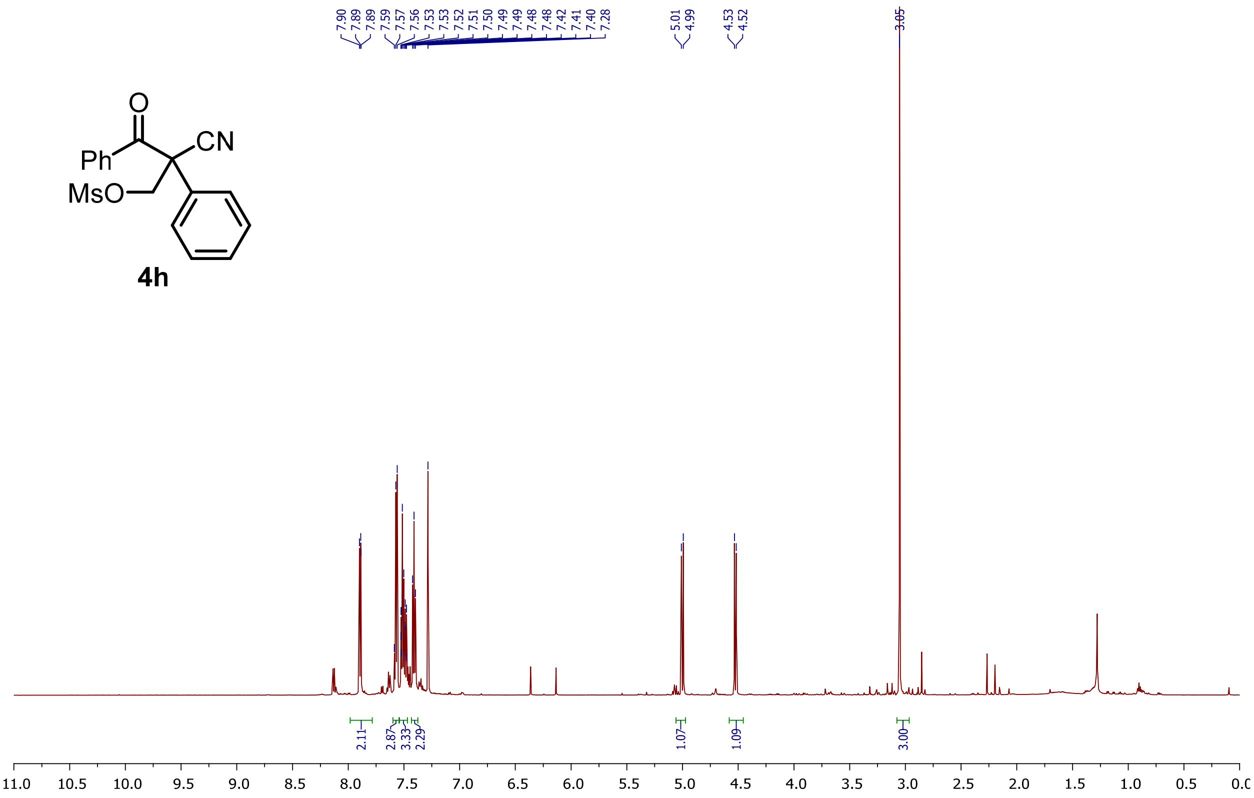

^{1}H NMR spectra of **4h**

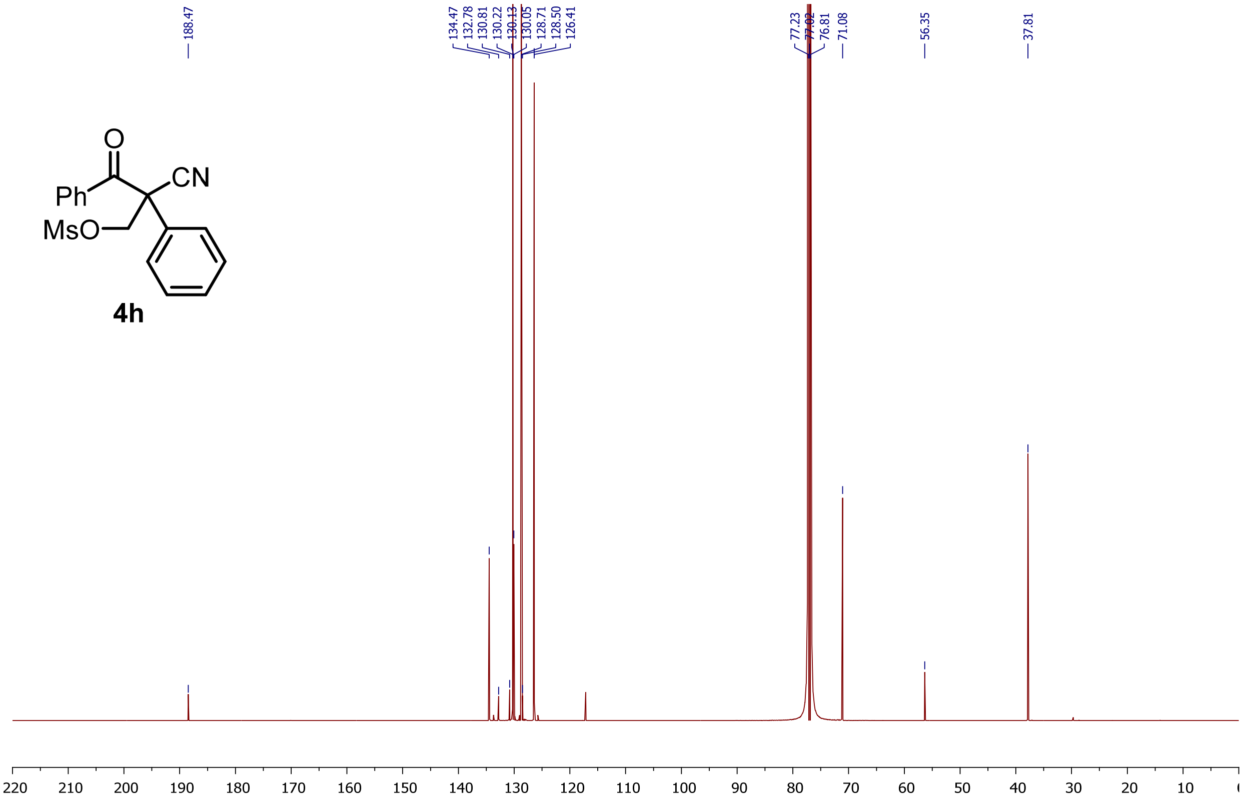

^{13}C NMR spectra of **4h**

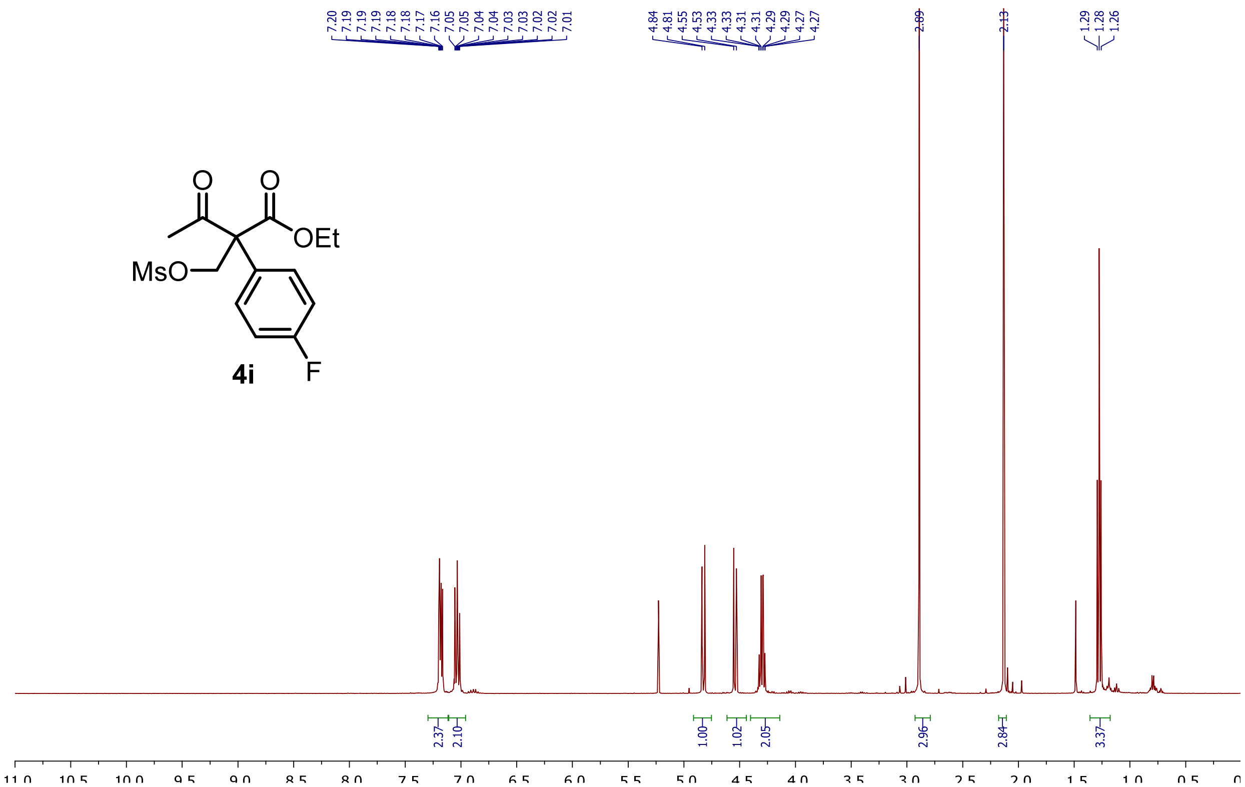

^{1}H NMR spectra of **4i**

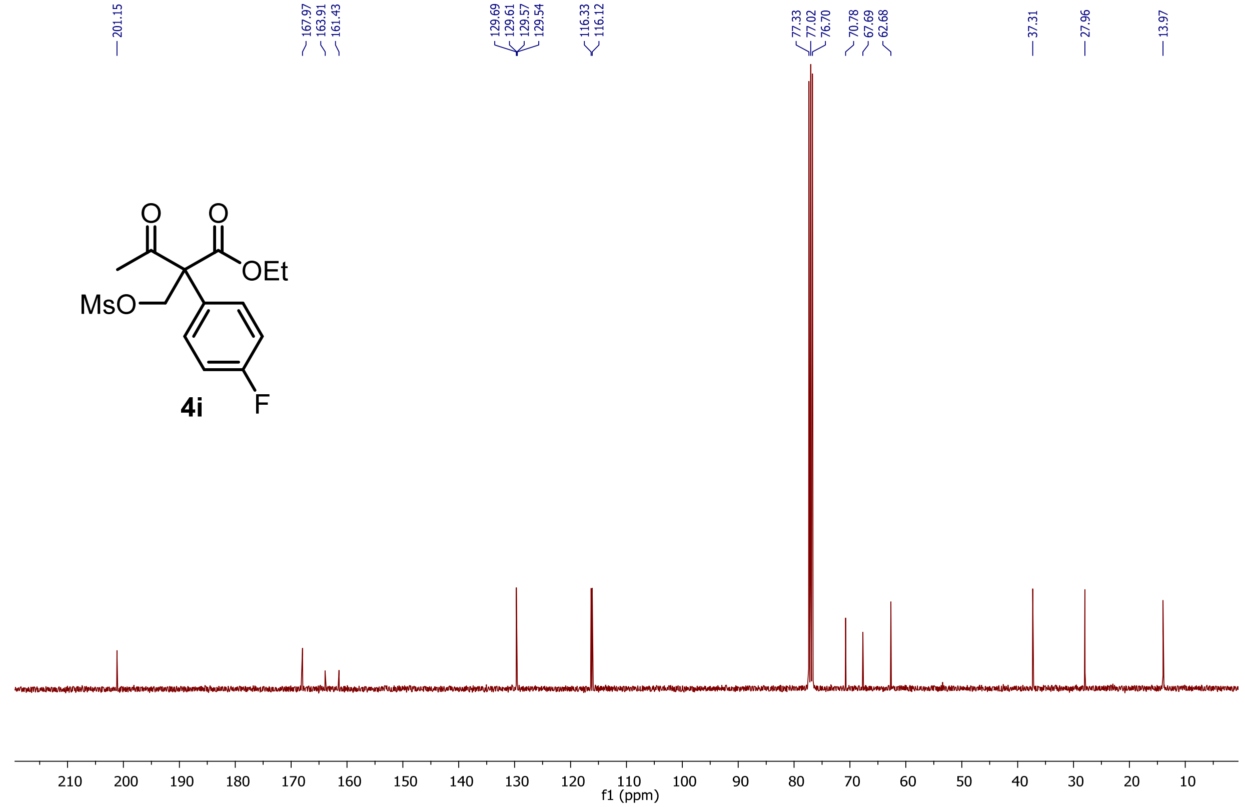

^{13}C NMR spectra of **4i**

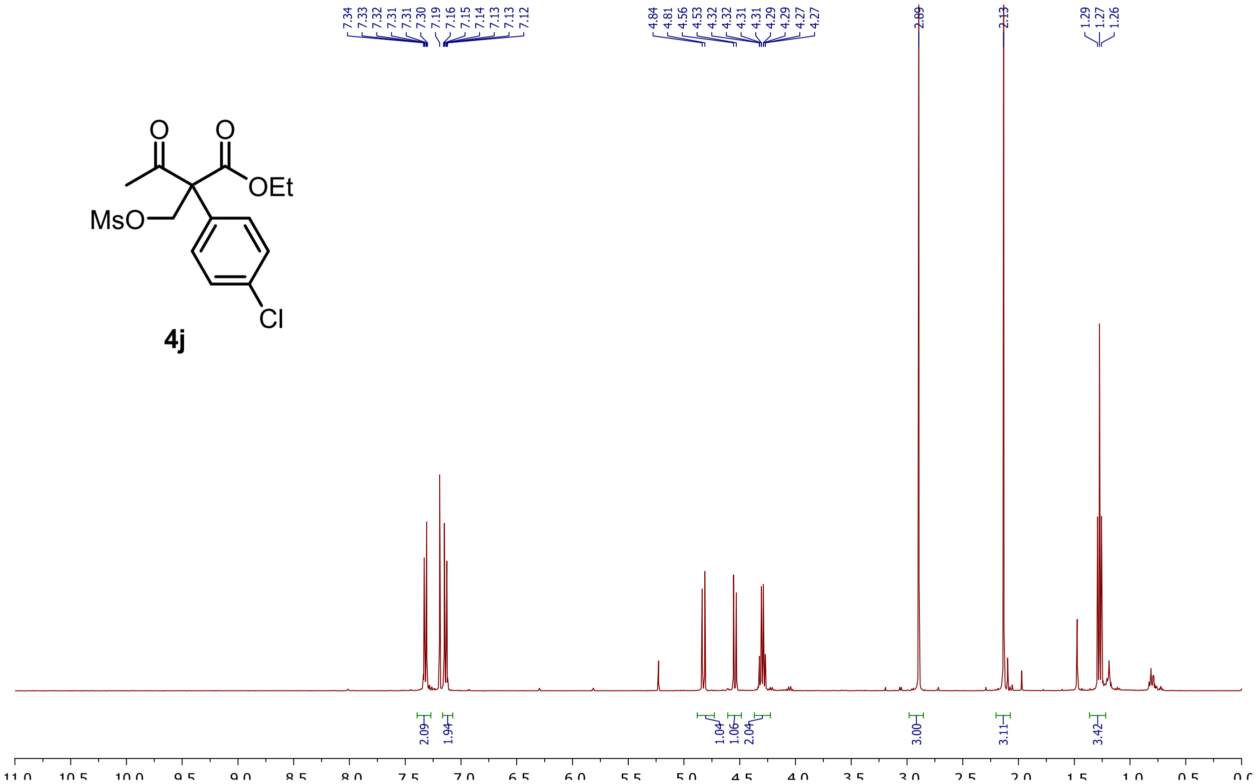

^{1}H NMR spectra of **4j**

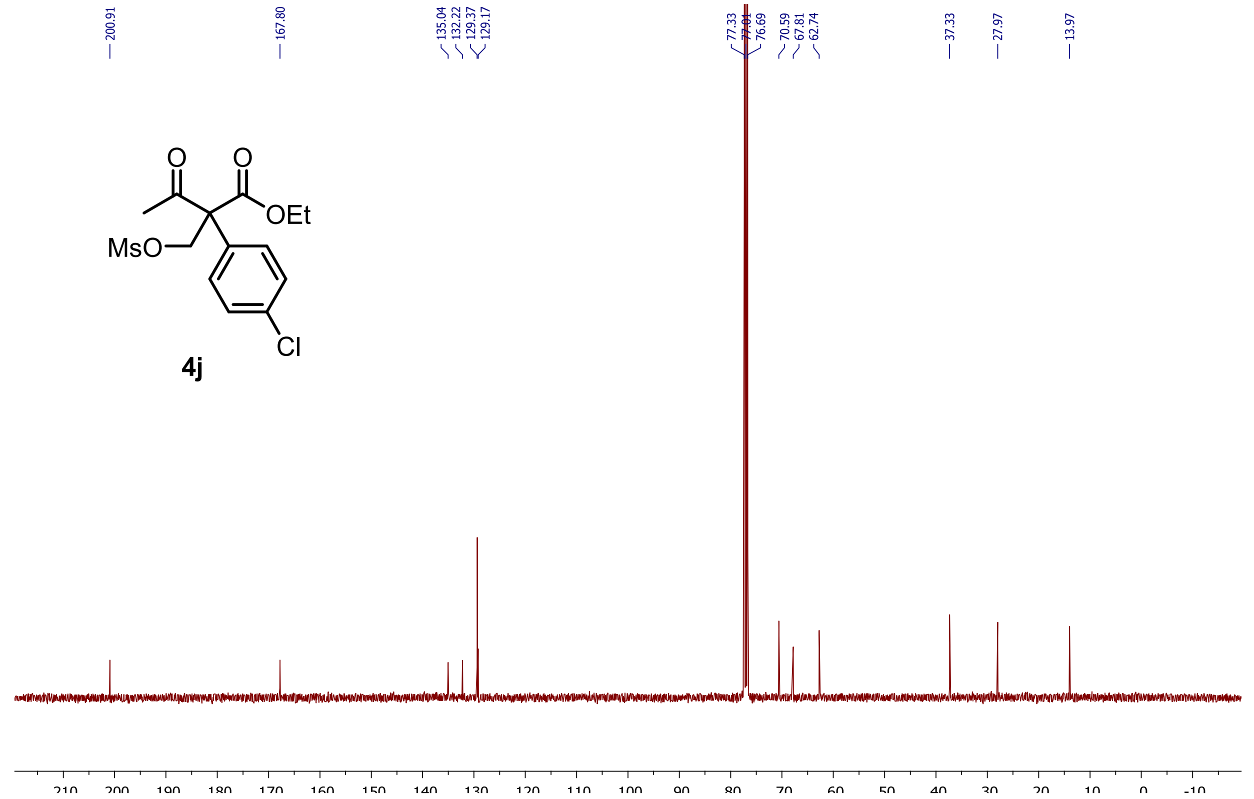

^{13}C NMR spectra of **4j**

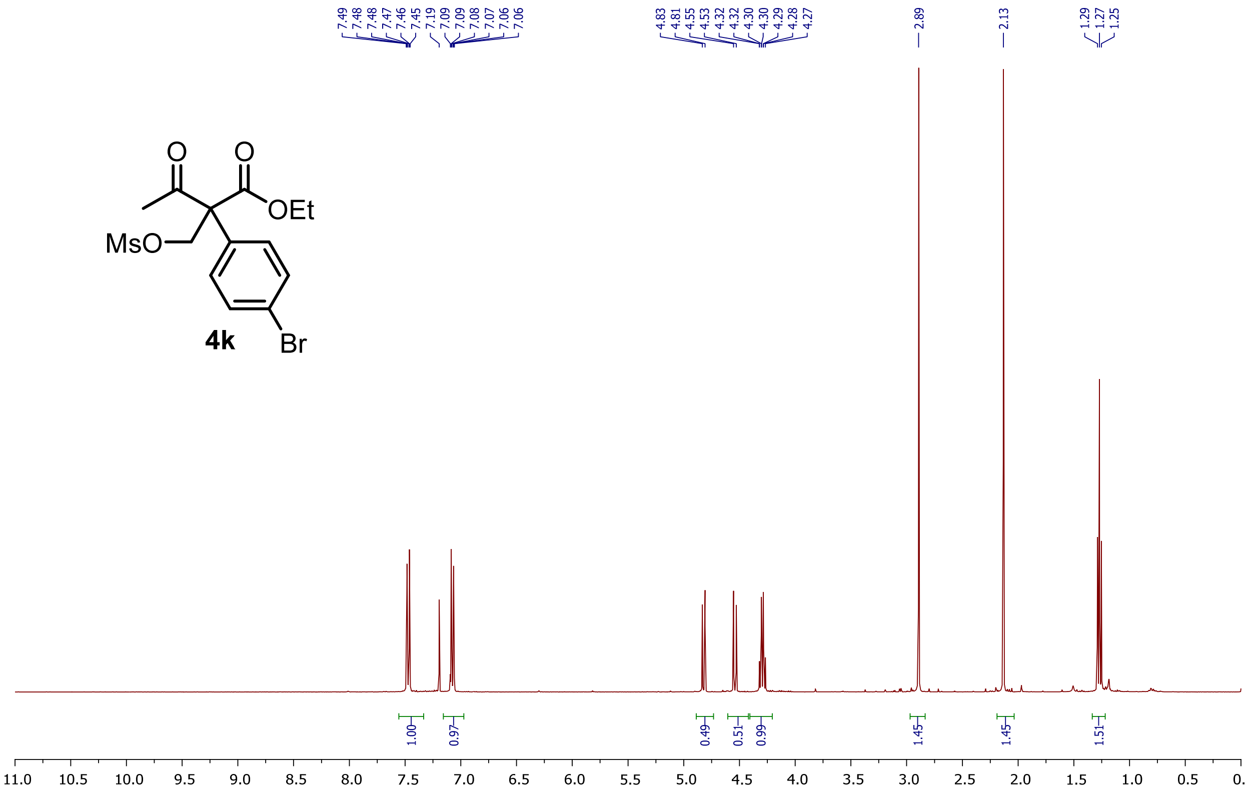

^{1}H NMR spectra of **4k**

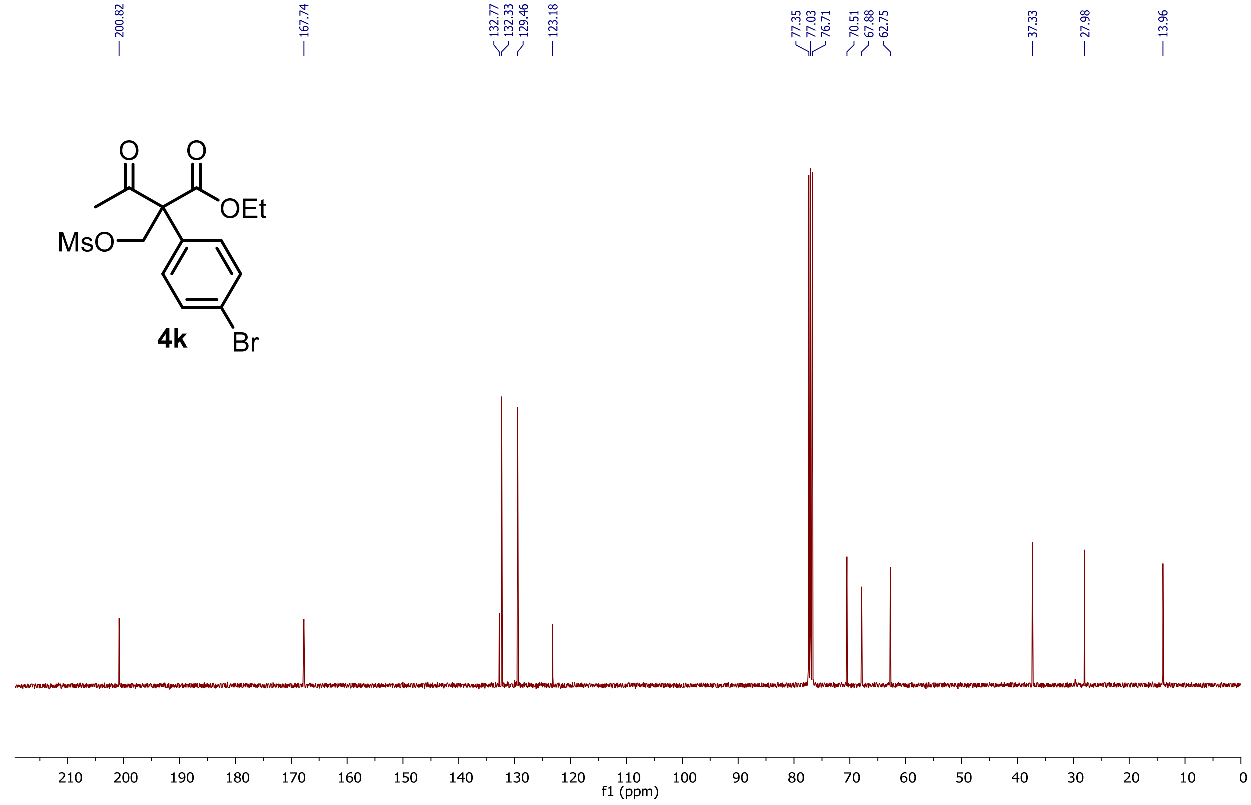

^{13}C NMR spectra of **4k**

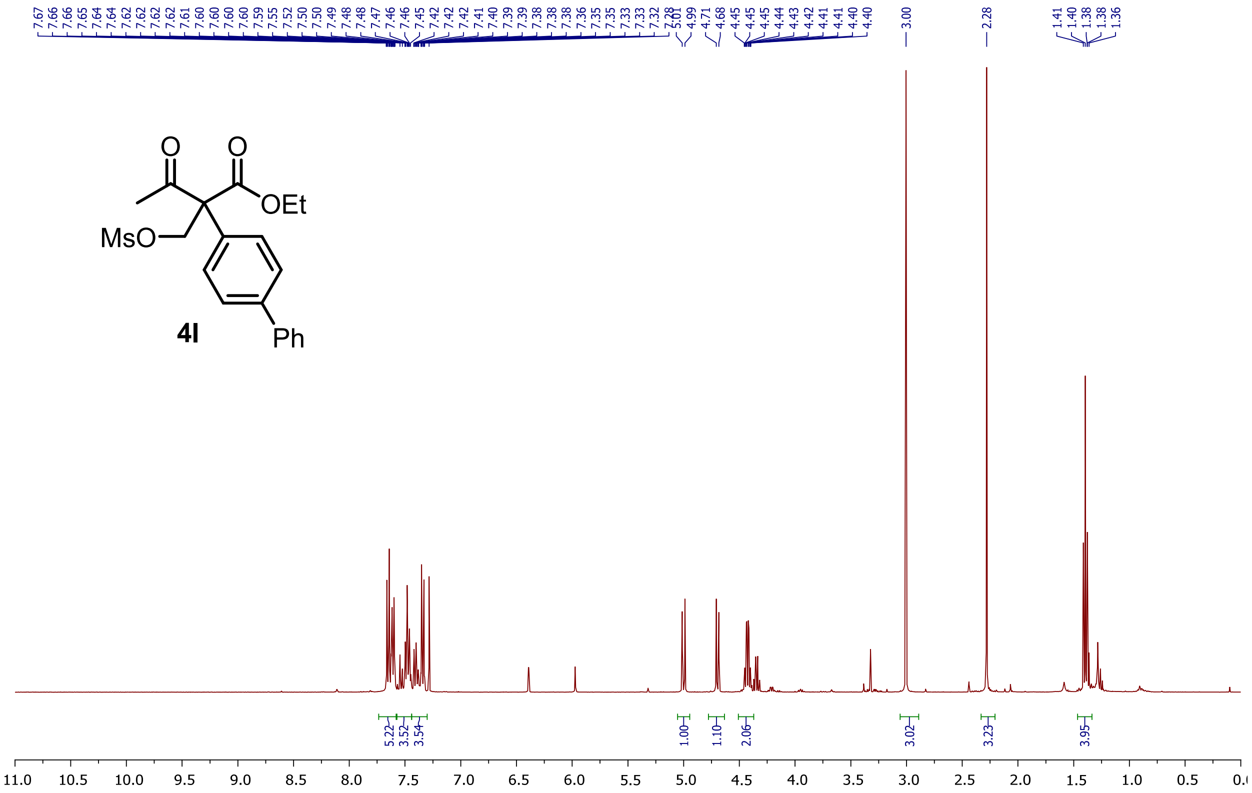

^{1}H NMR spectra of **4l**

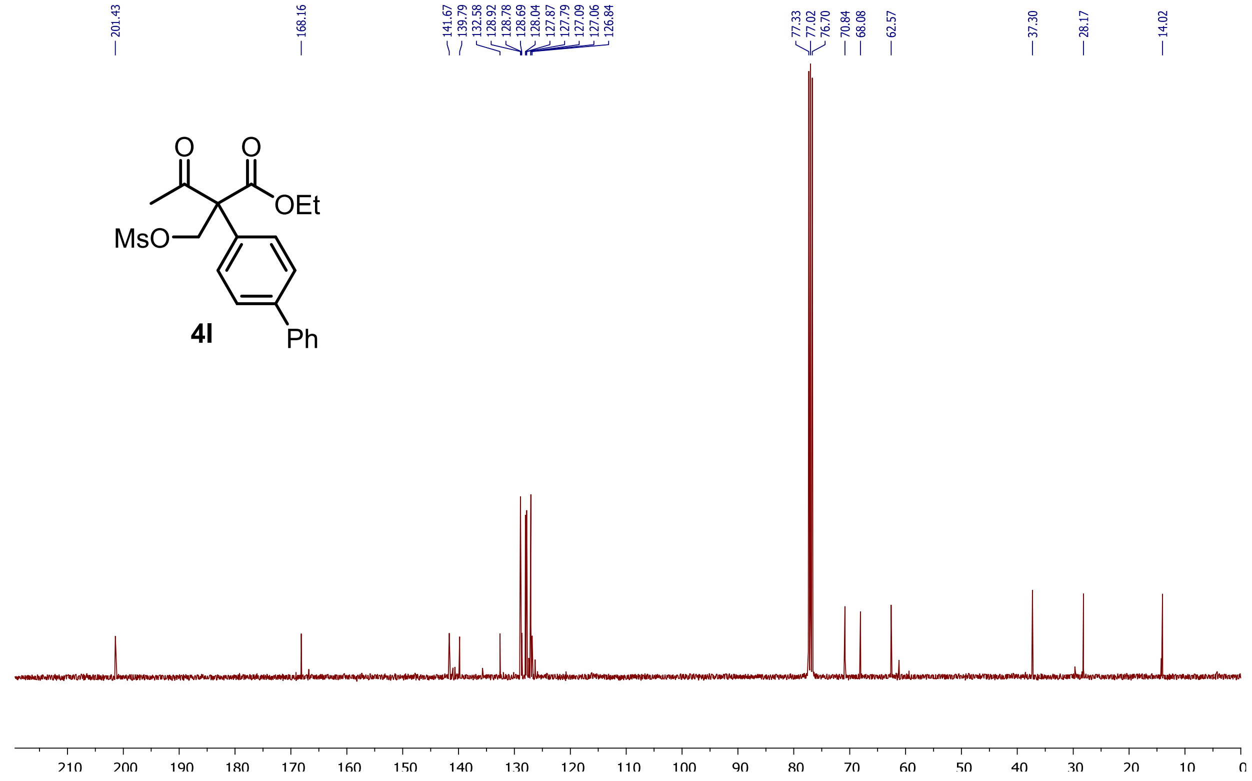

^{13}C NMR spectra of **4l**

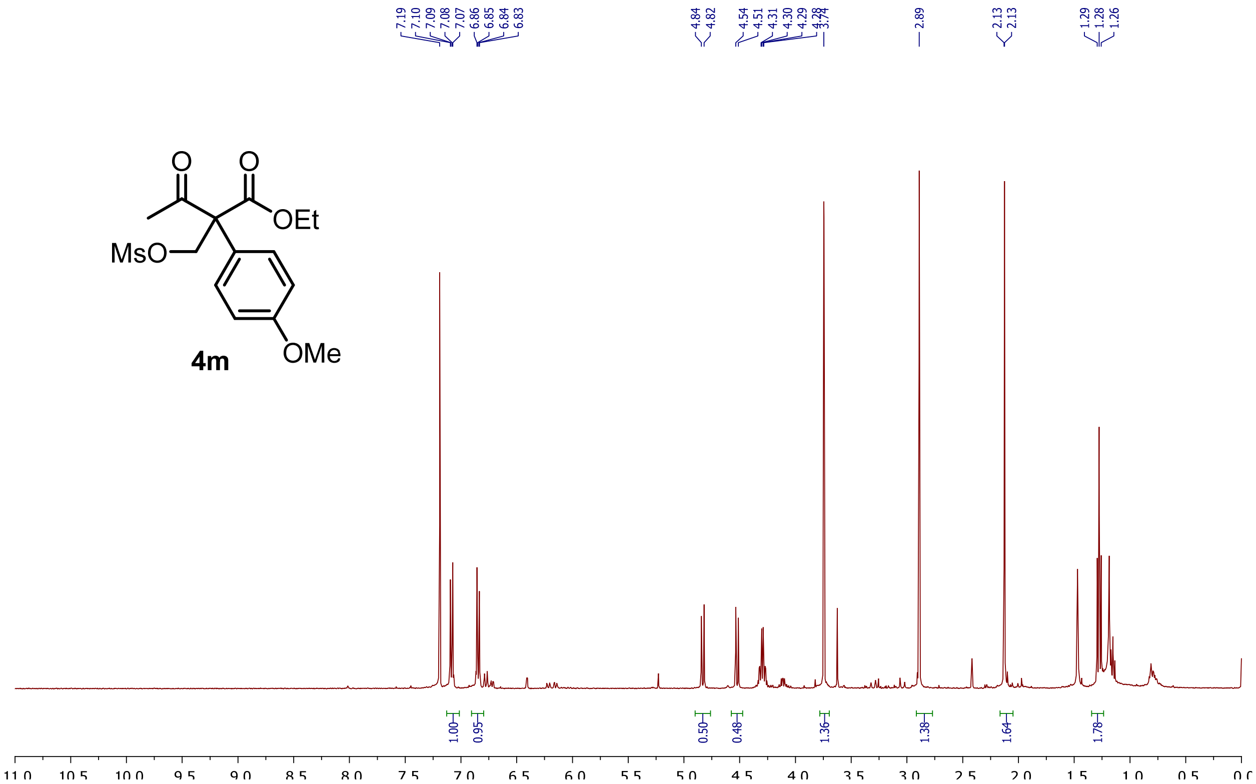

^{1}H NMR spectra of **4m**

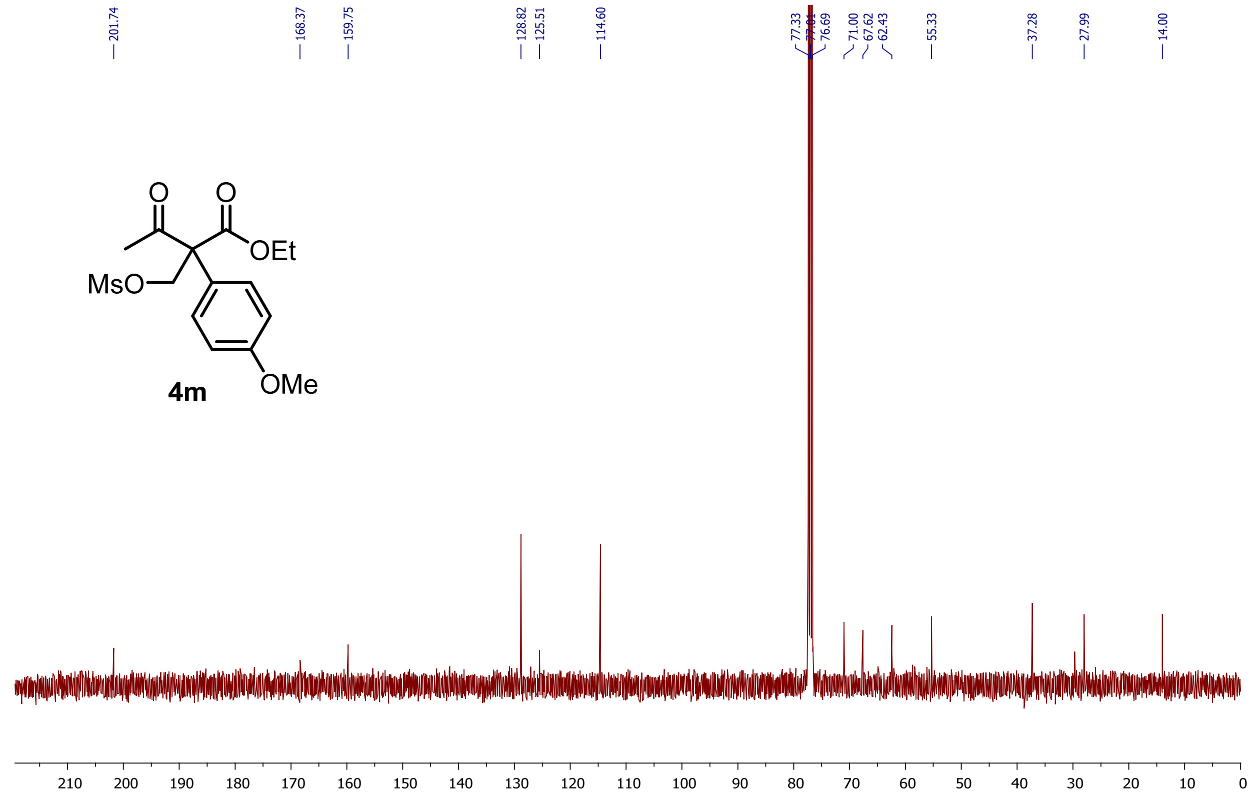

^{13}C NMR spectra of **4m**

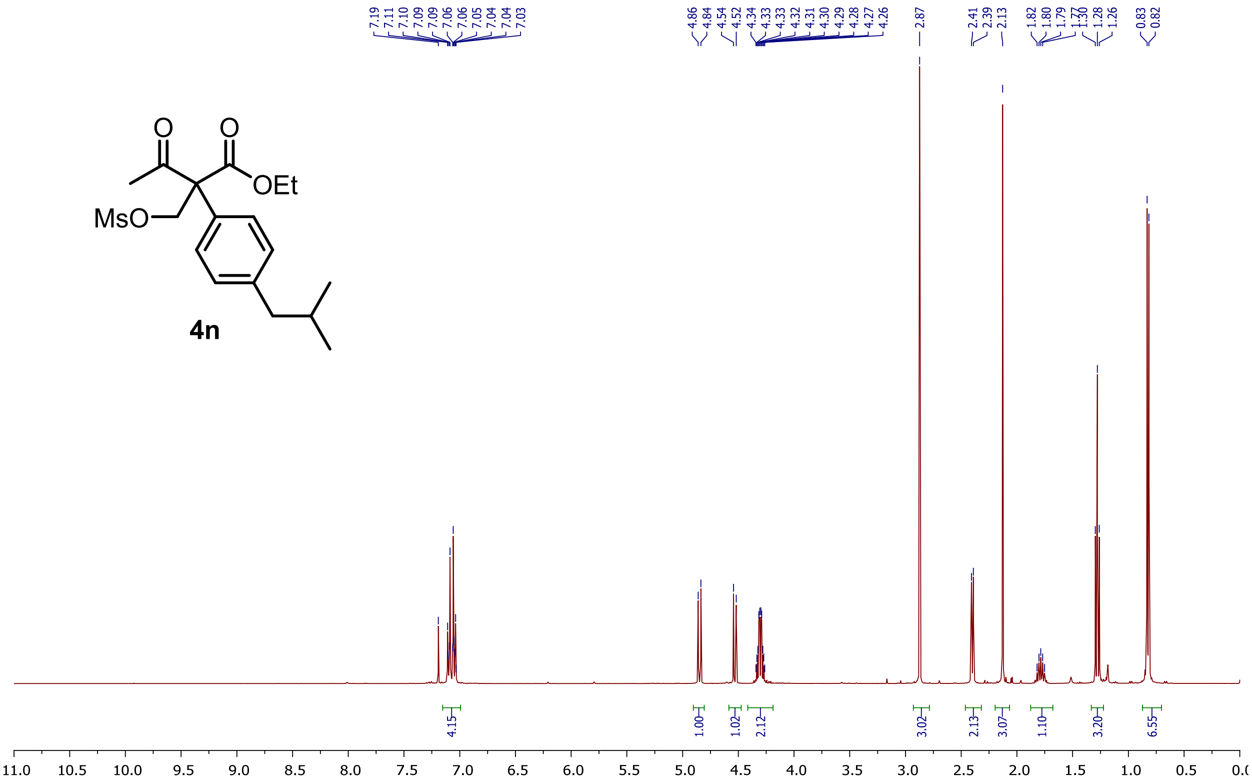

^{1}H NMR spectra of **4n**

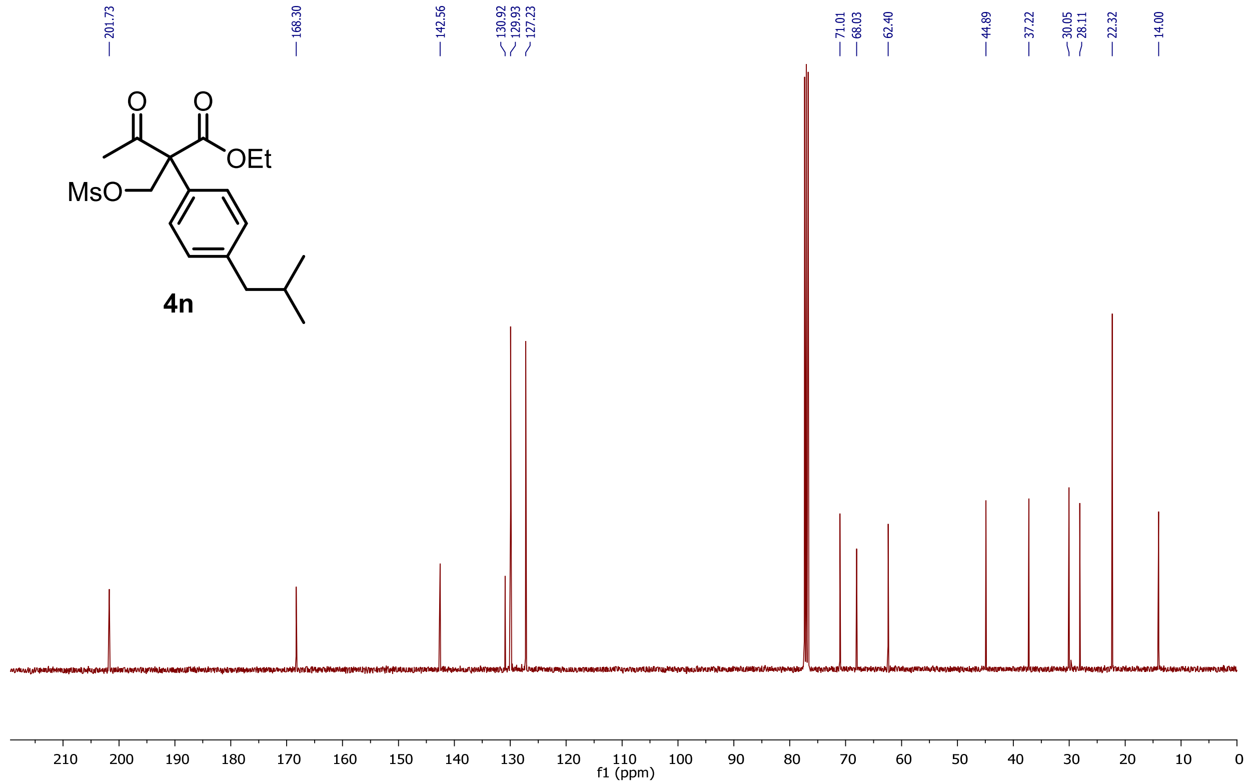

^{13}C NMR spectra of **4n**

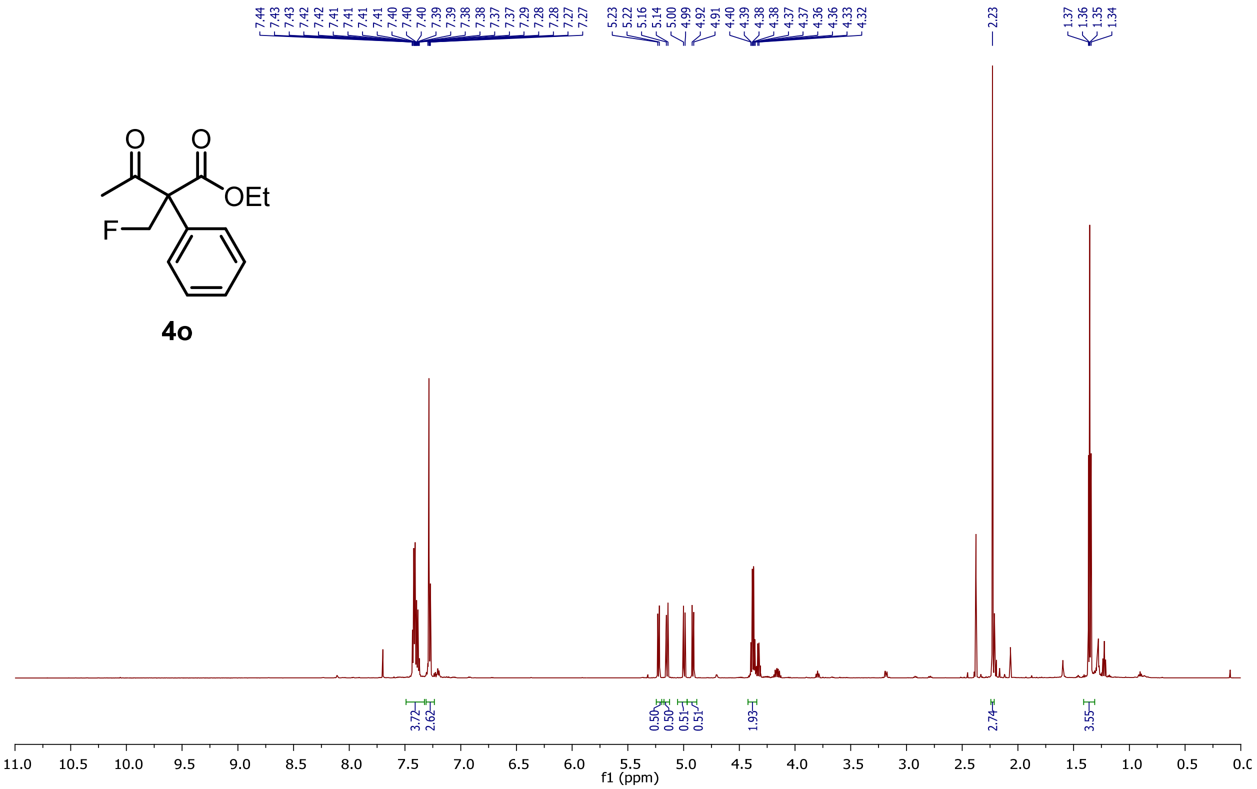

^{1}H NMR spectra of **4o**

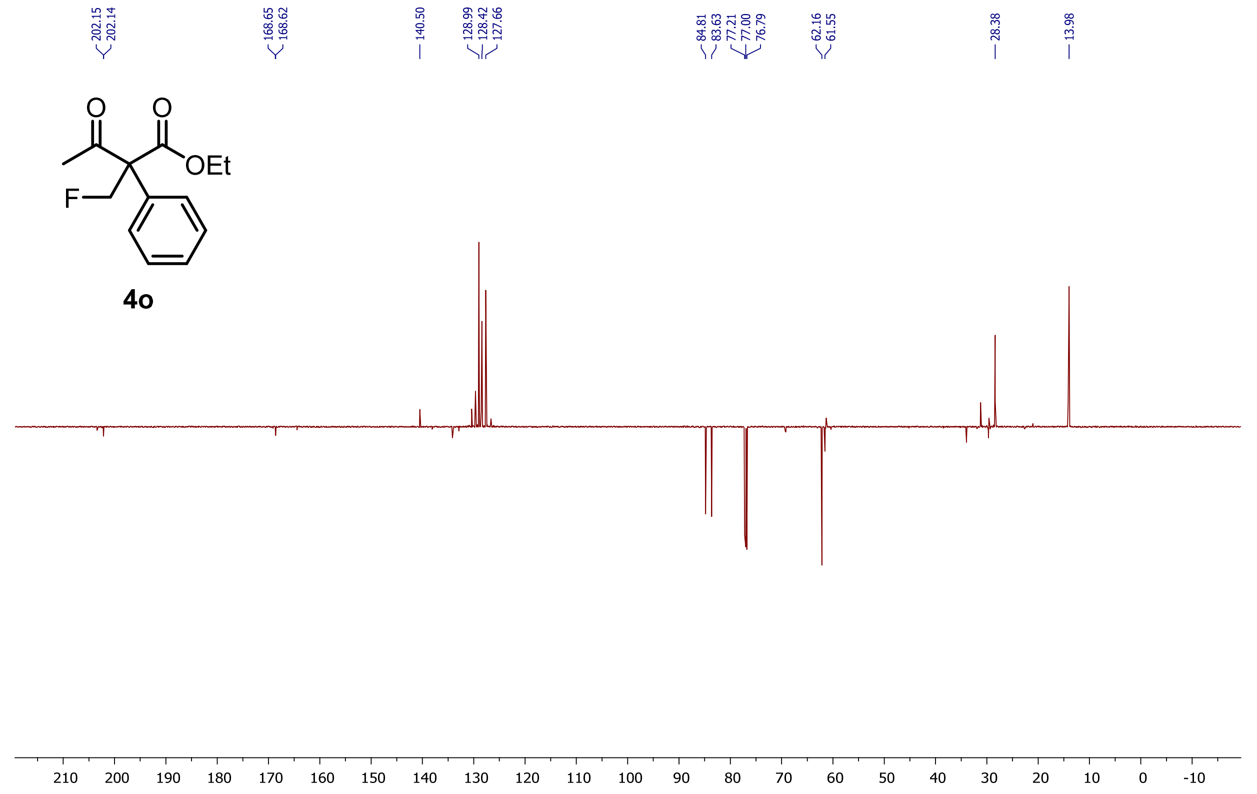

^{13}C NMR spectra of **4o**

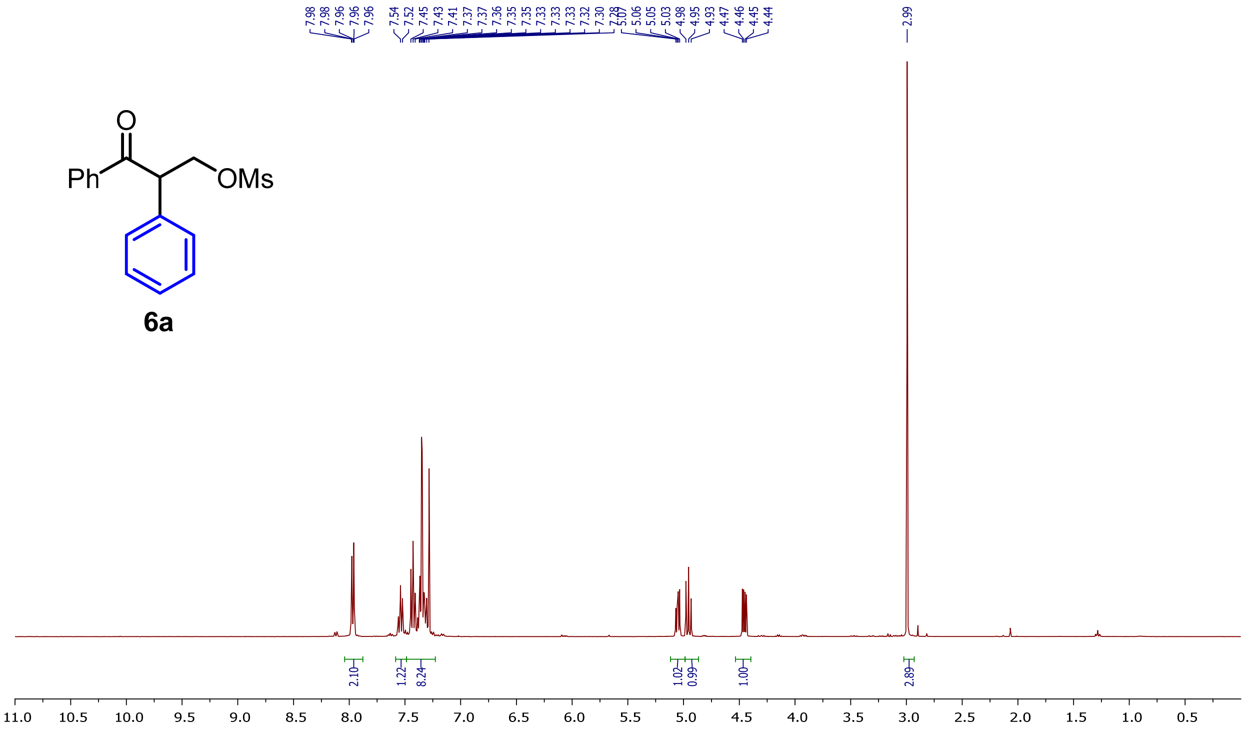

^{1}H NMR spectra of **6a**

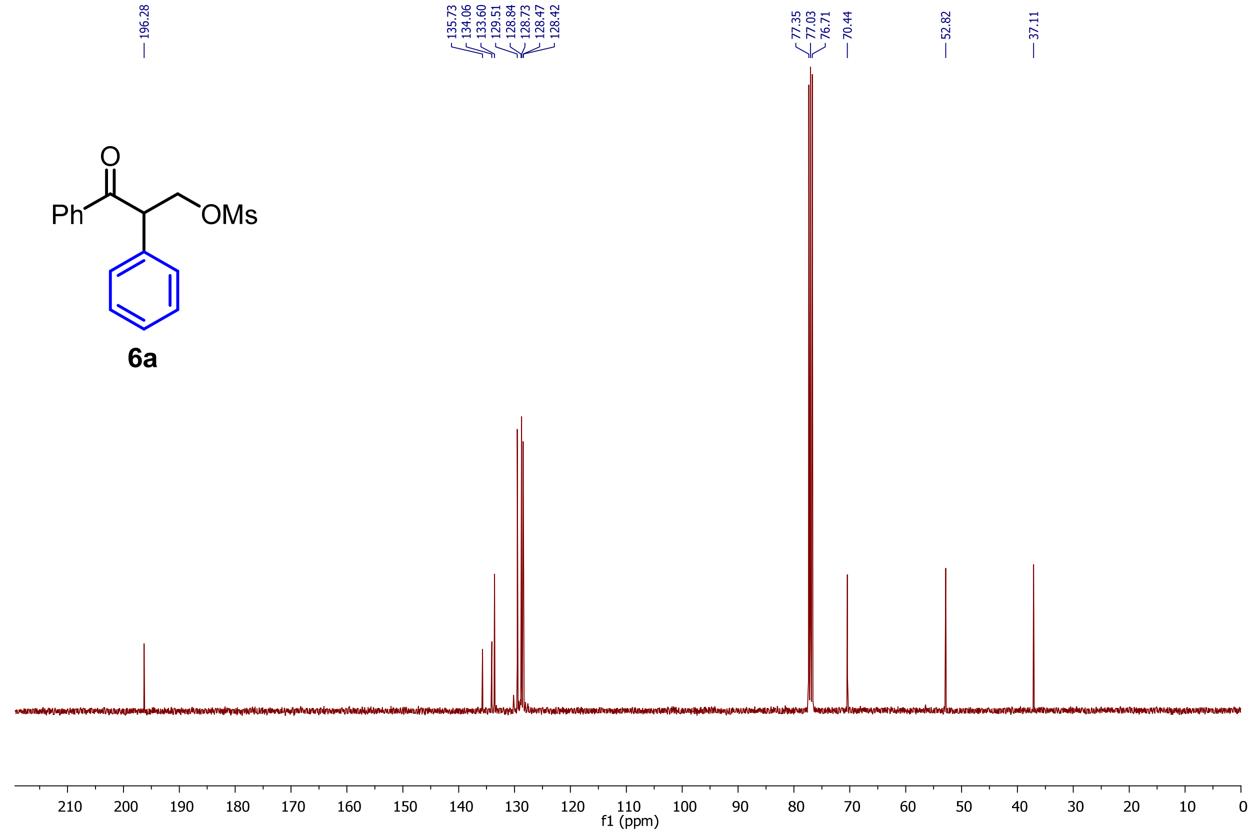

^{13}C NMR spectra of **6a**

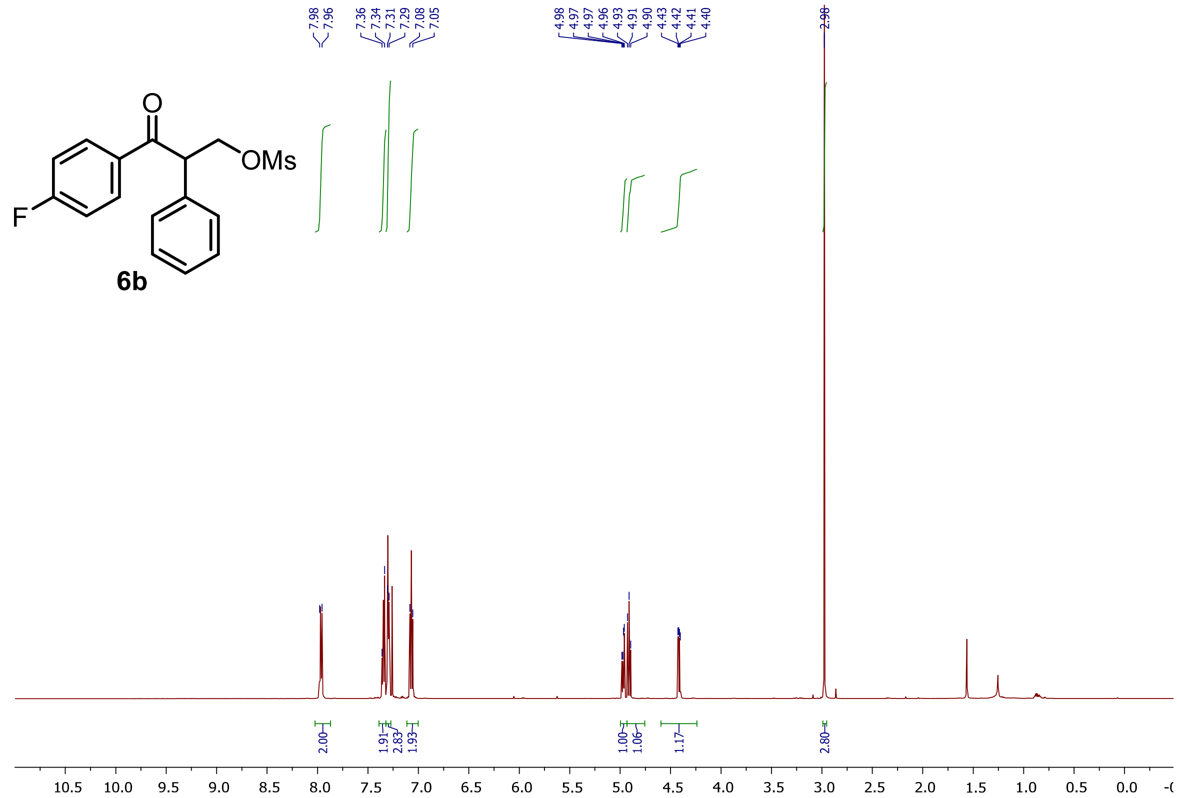

^{1}H NMR spectra of **6b**

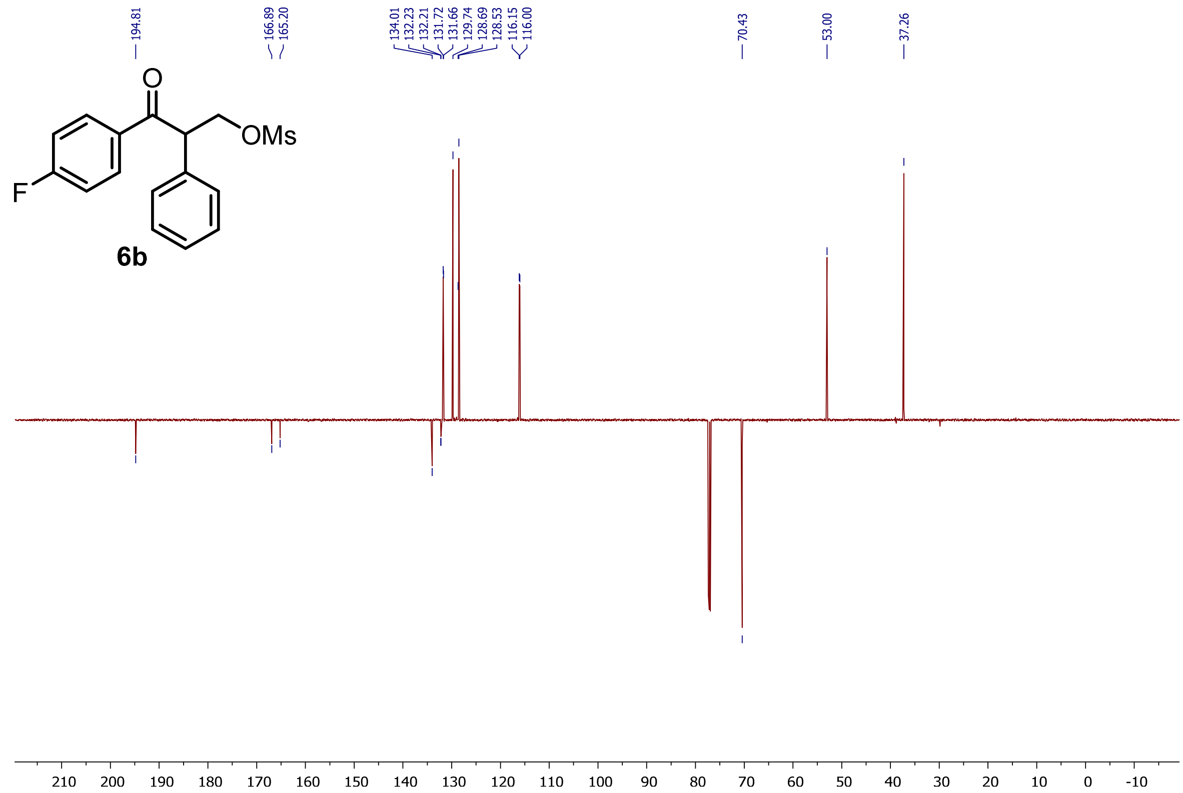

^{13}C NMR spectra of **6b**

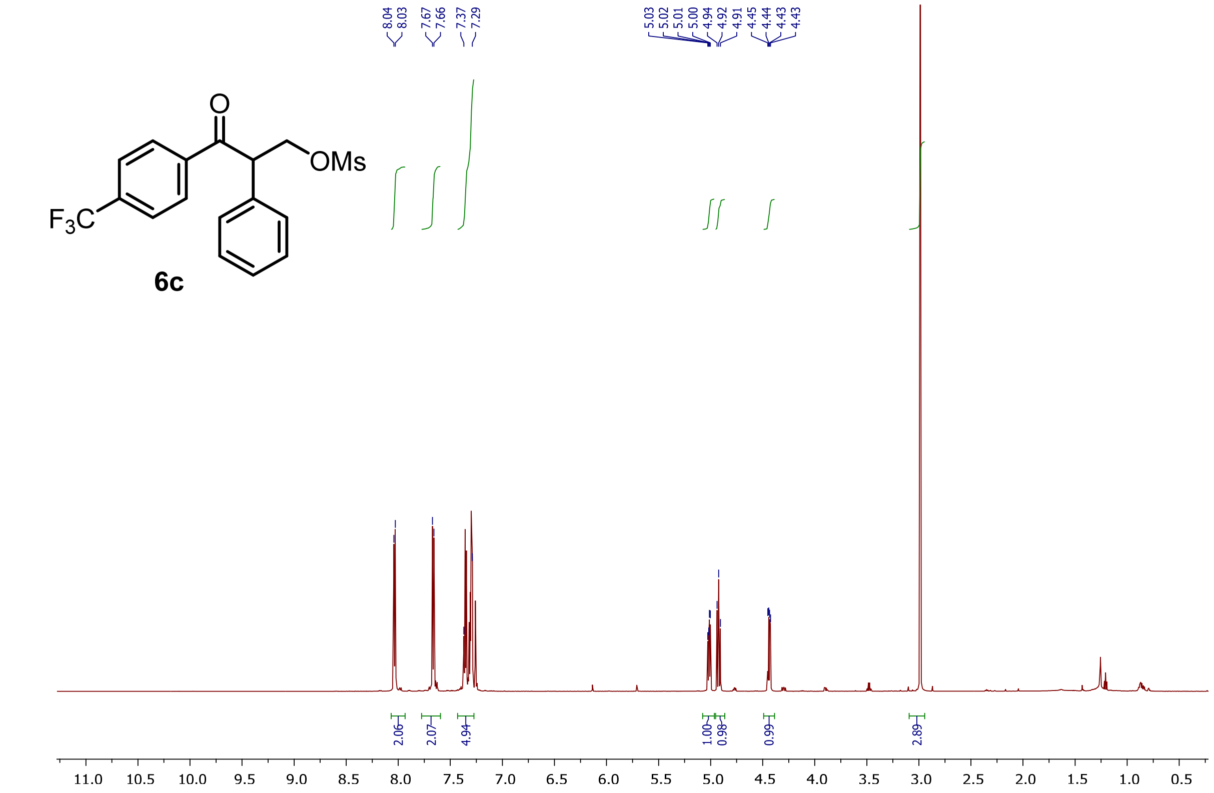

^{1}H NMR spectra of **6c**

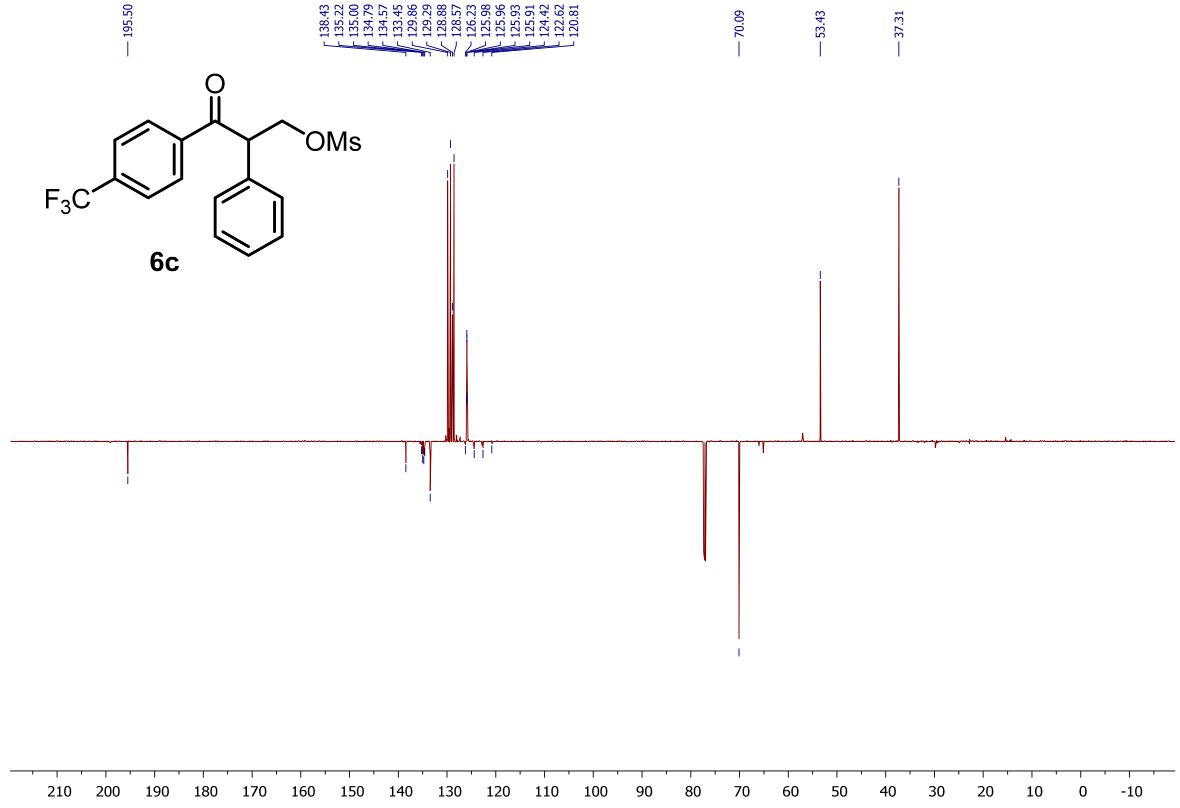

^{13}C NMR spectra of **6c**

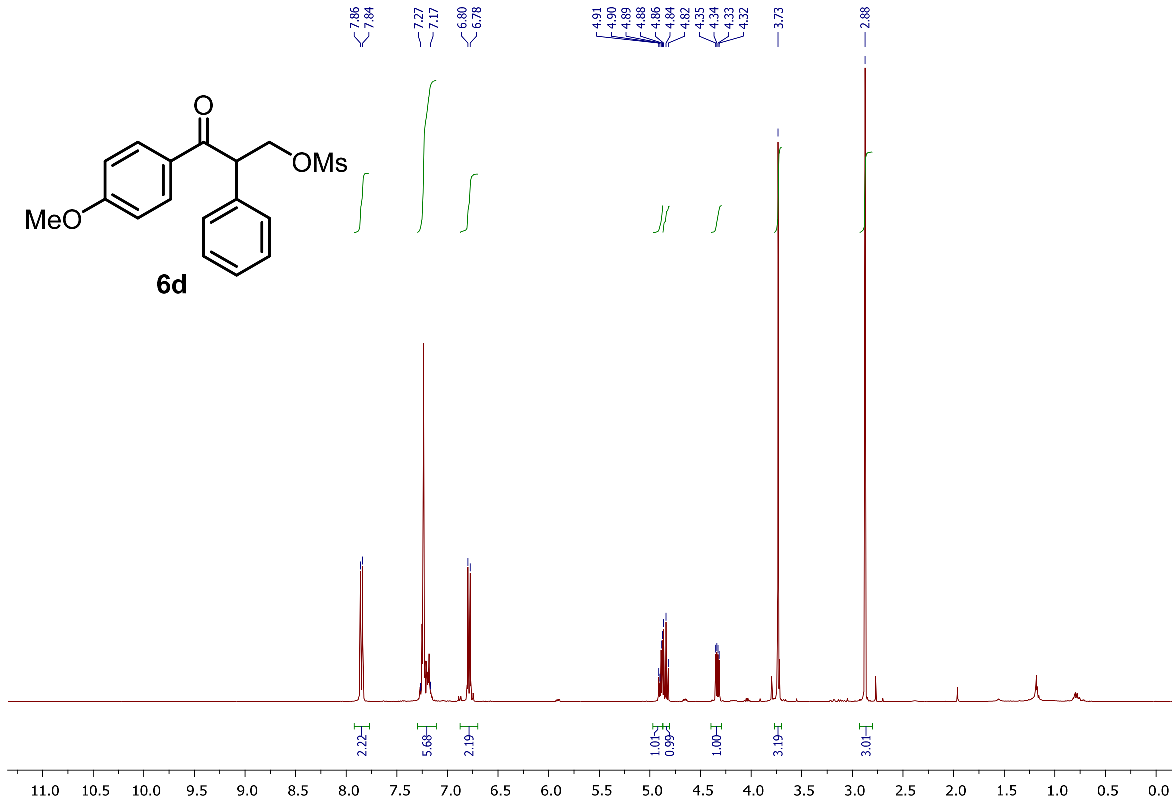

^{1}H NMR spectra of **6d**

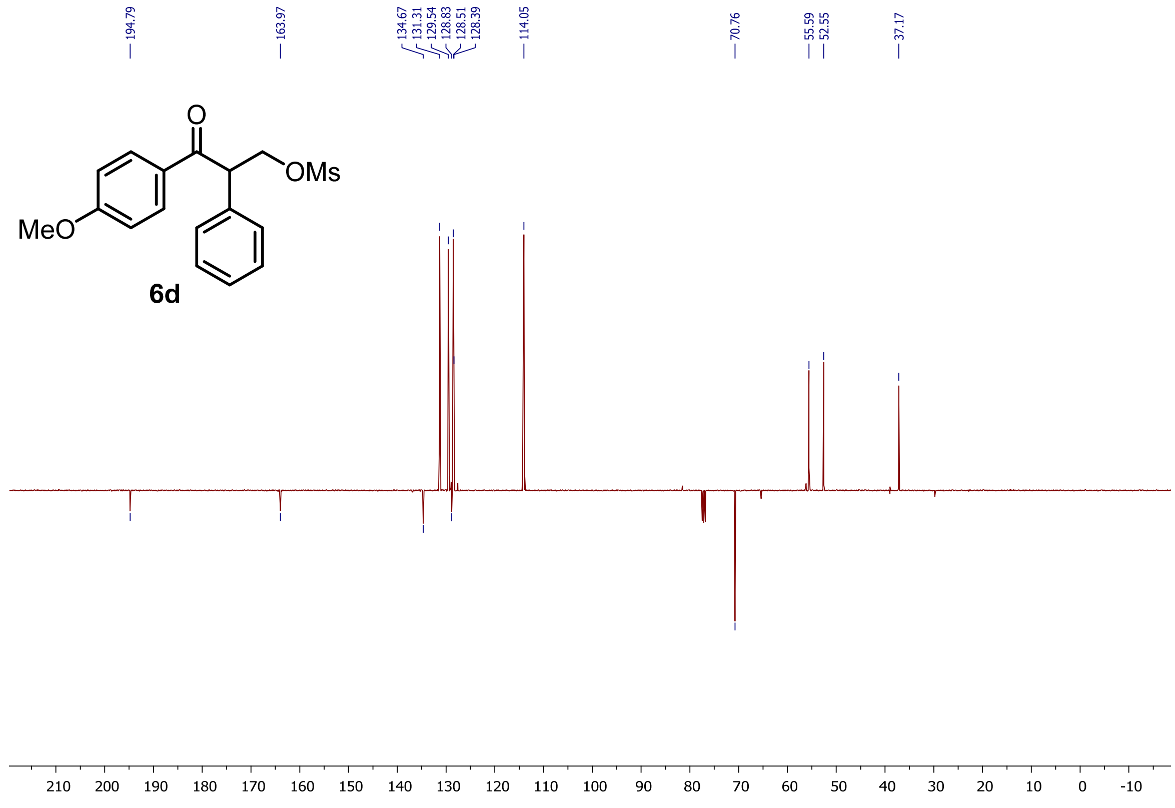

^{13}C NMR spectra of **6d**

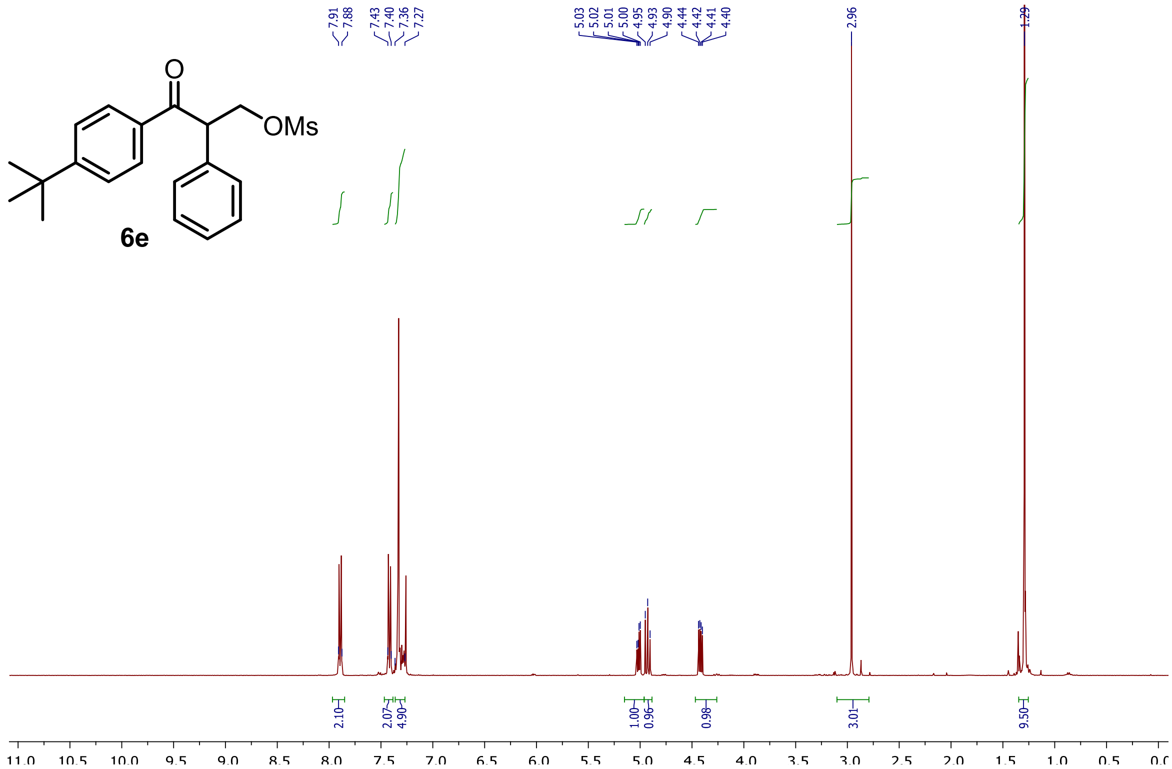

^{1}H NMR spectra of **6e**

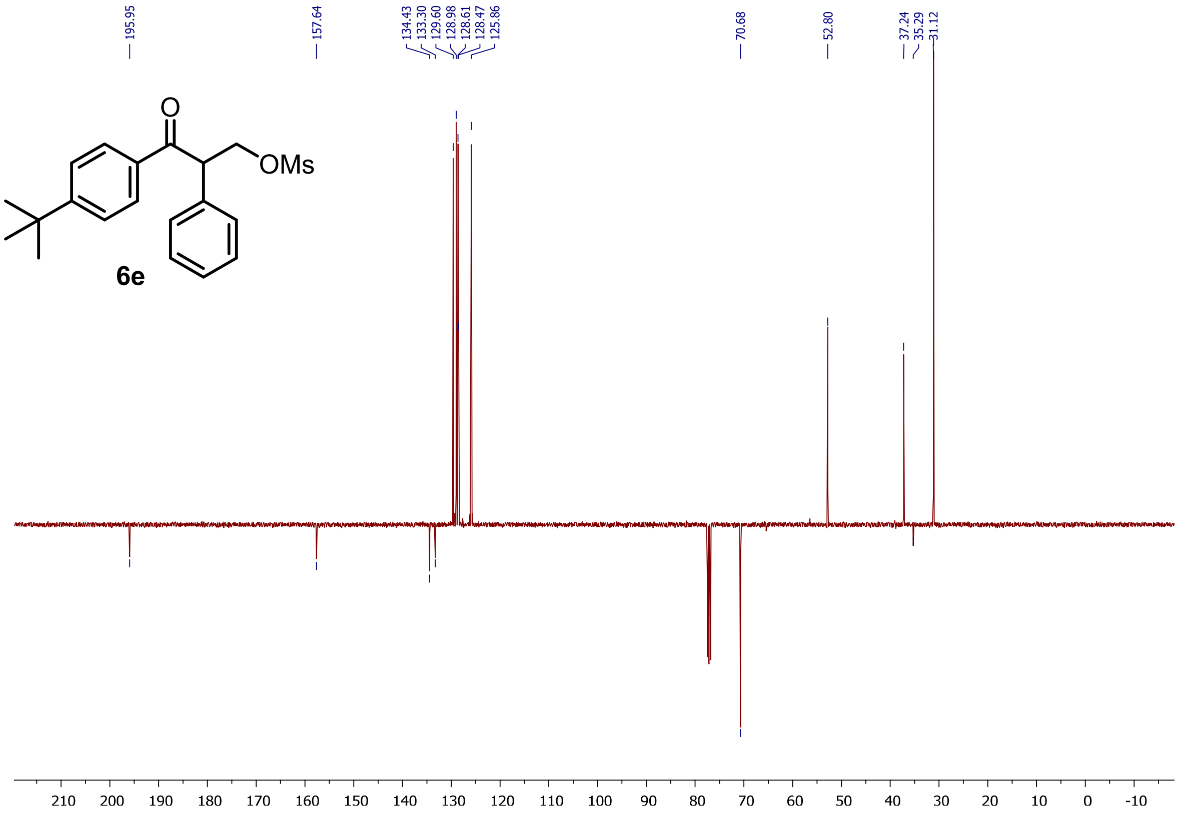

^{13}C NMR spectra of **6e**

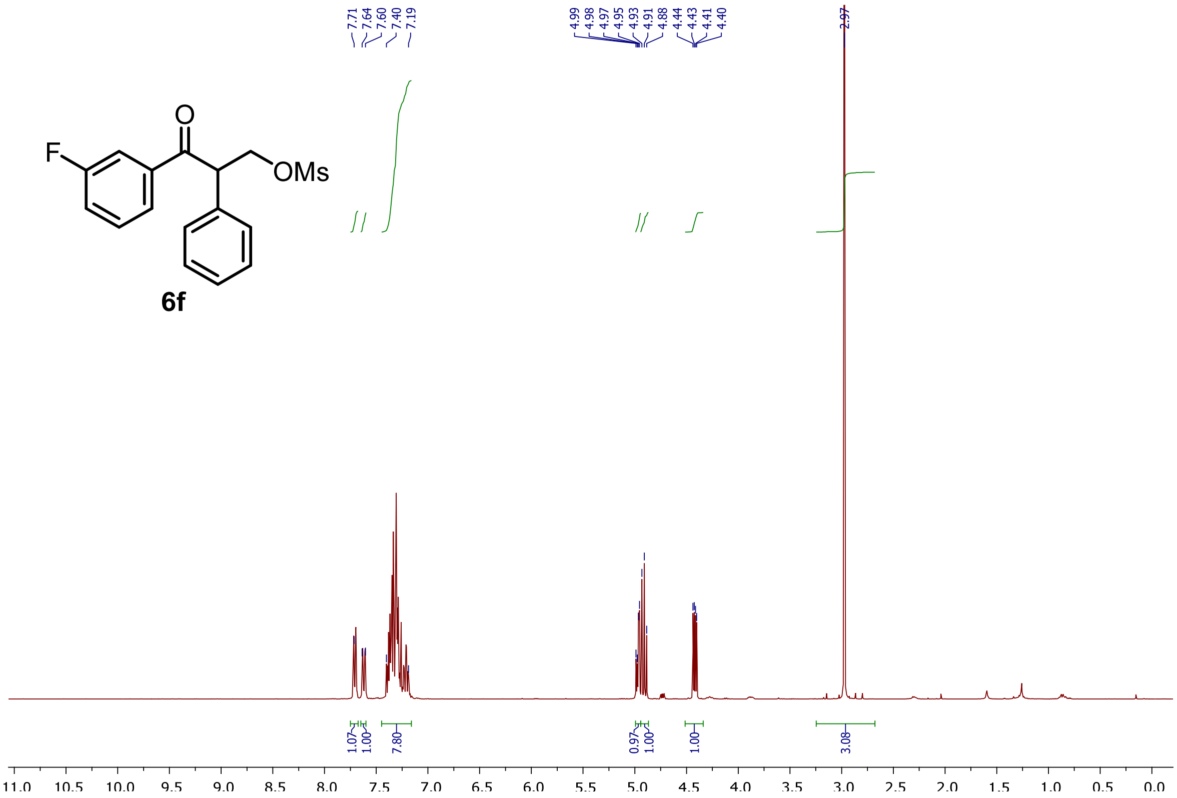

^{1}H NMR spectra of **6f**

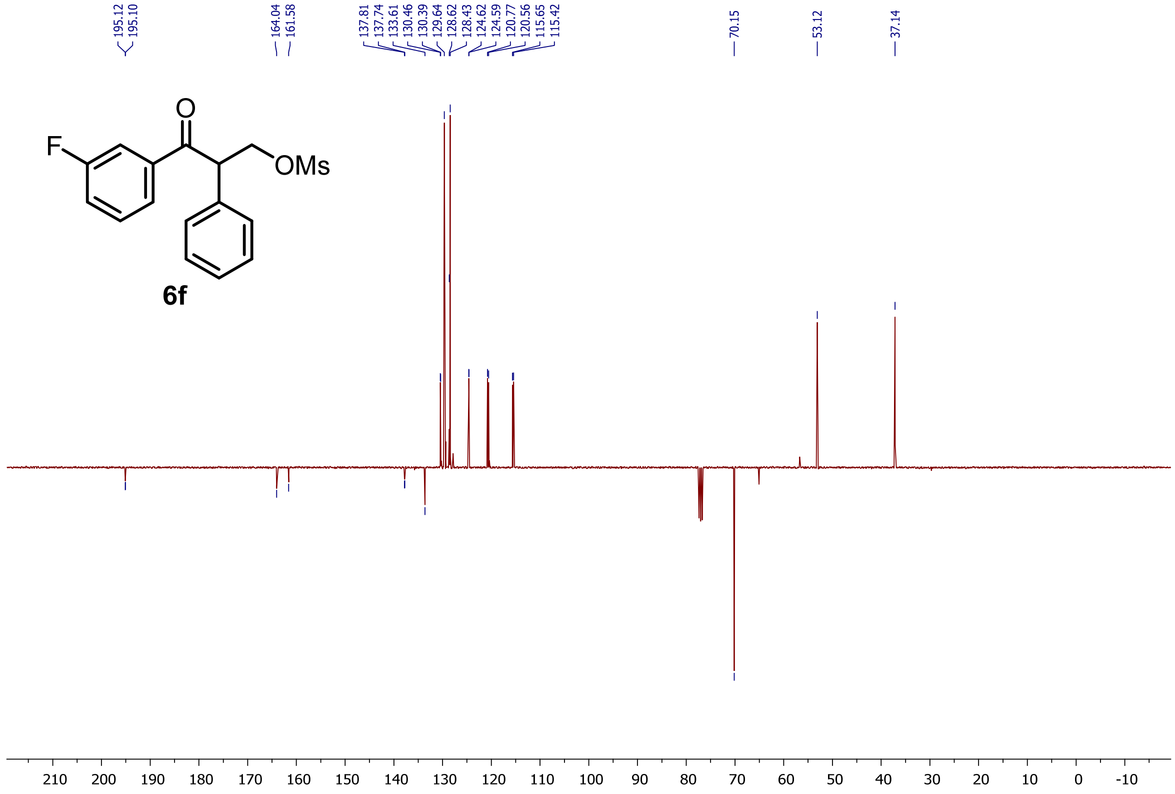

^{13}C NMR spectra of **6f**

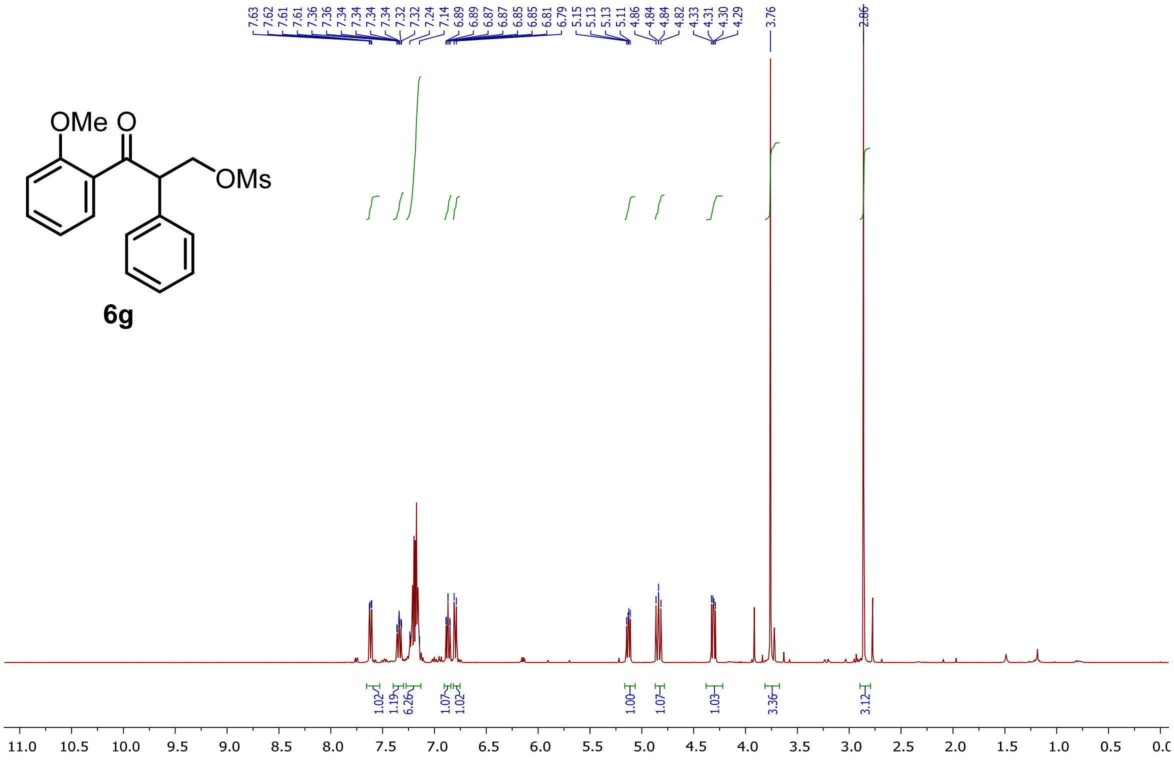

^{1}H NMR spectra of **6g**

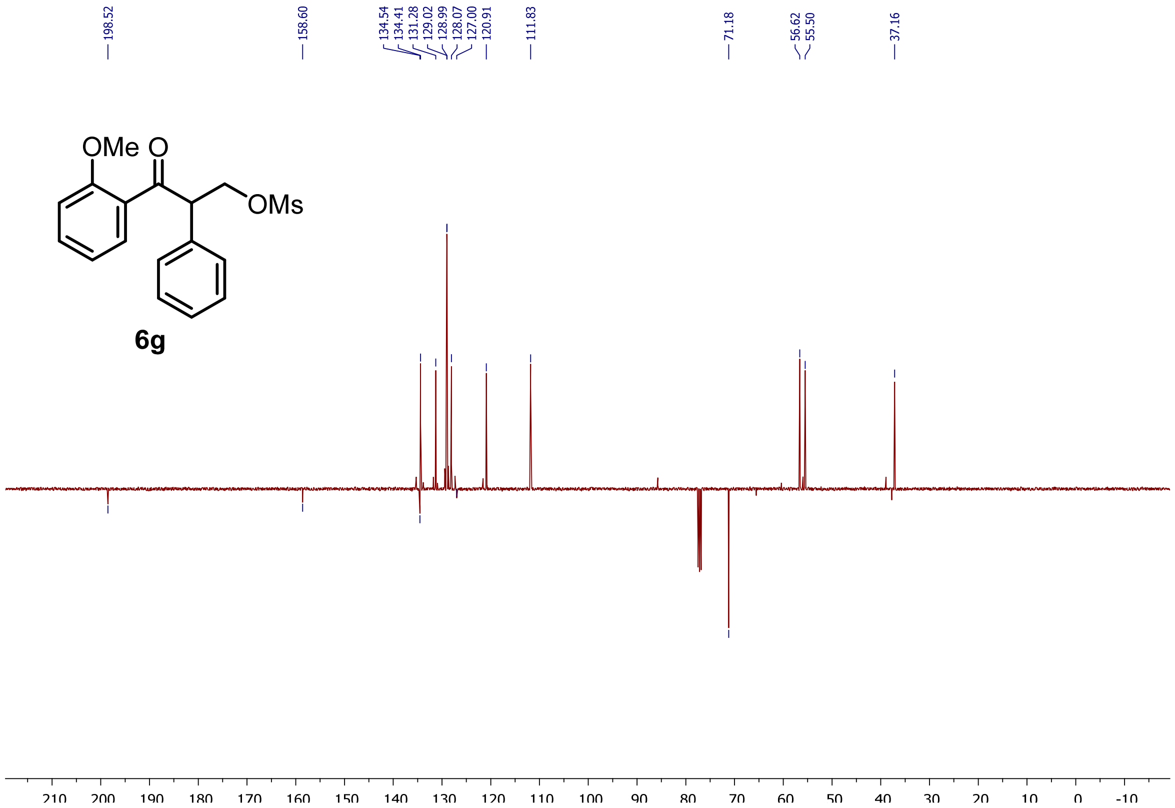

^{13}C NMR spectra of **6g**

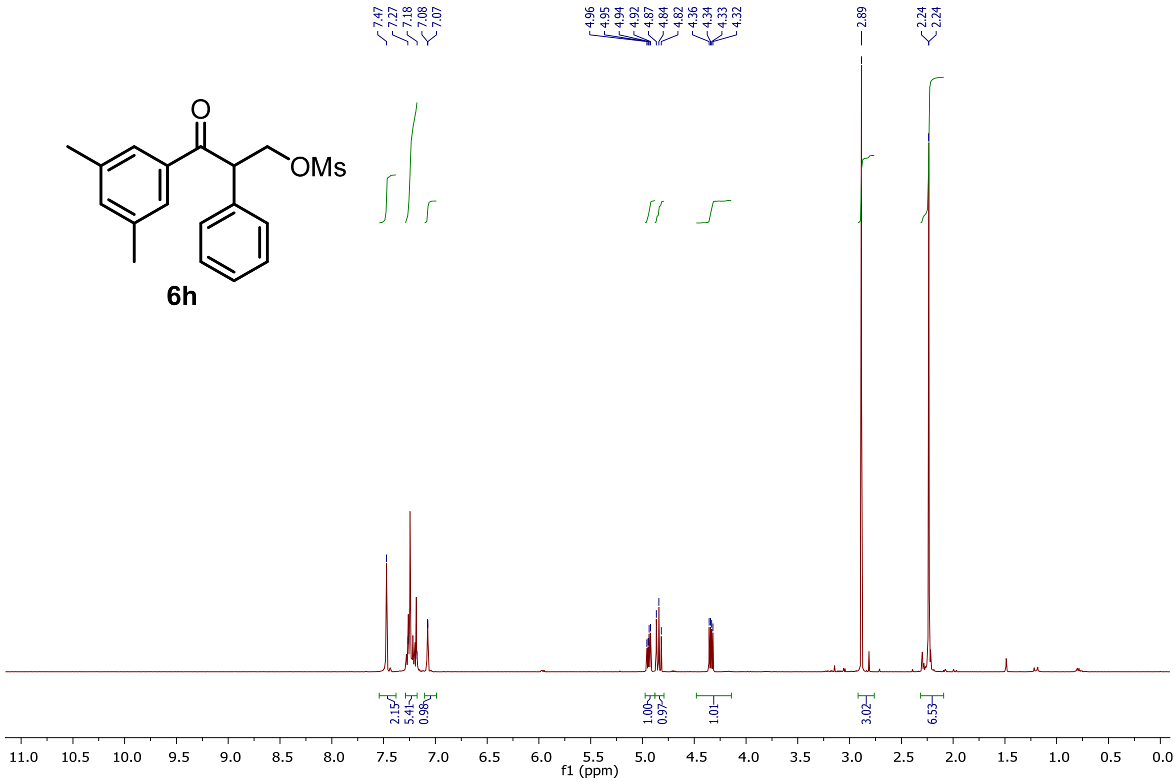

^{1}H NMR spectra of **6h**

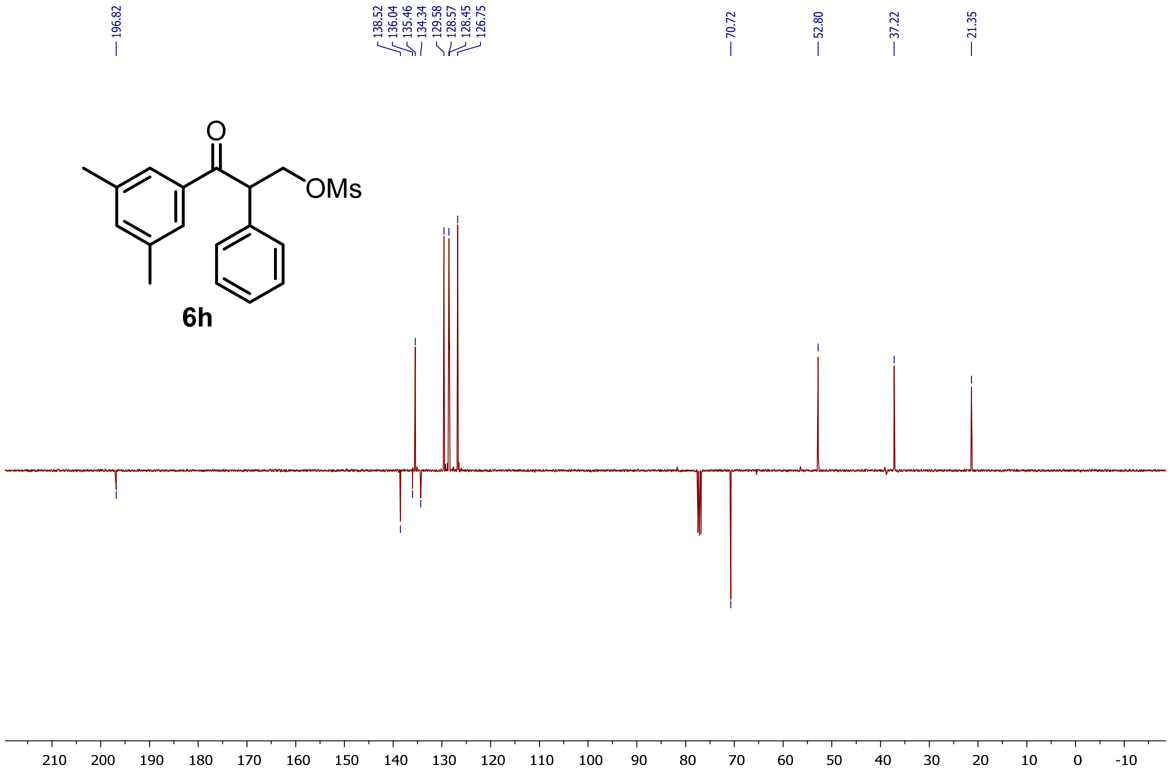

^{13}C NMR spectra of **6h**

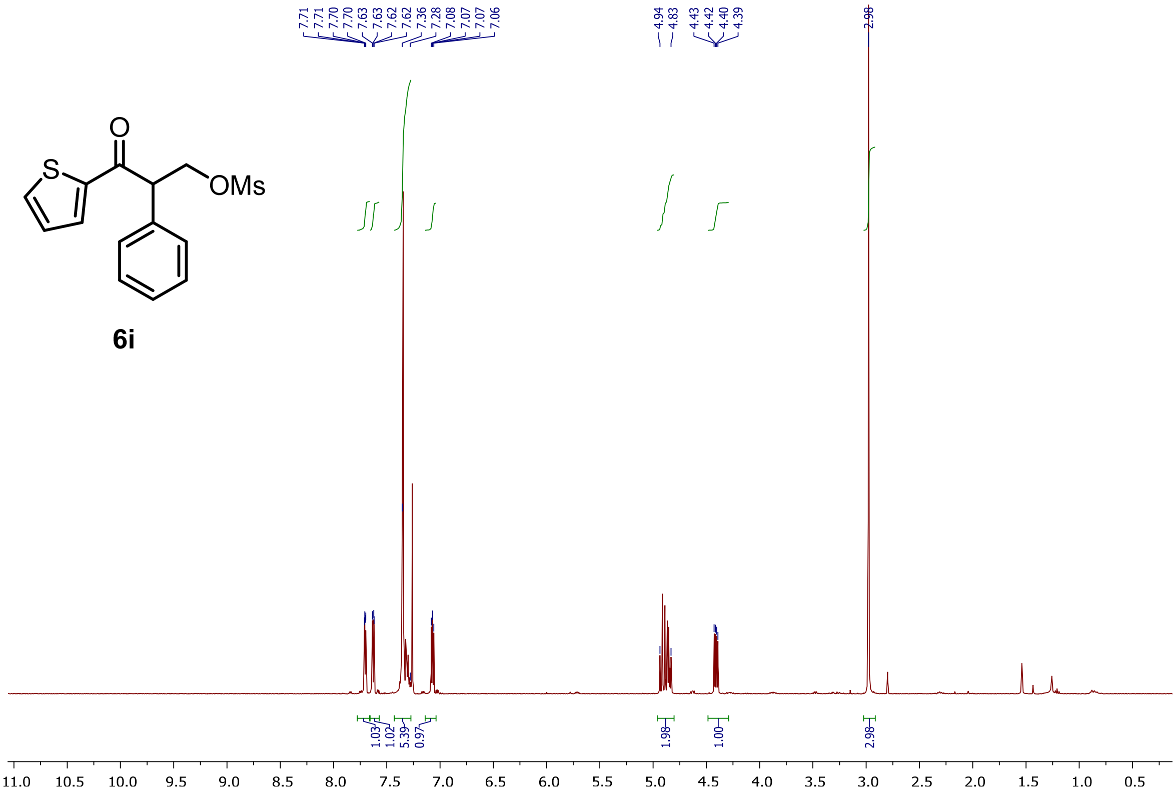

^{1}H NMR spectra of **6i**

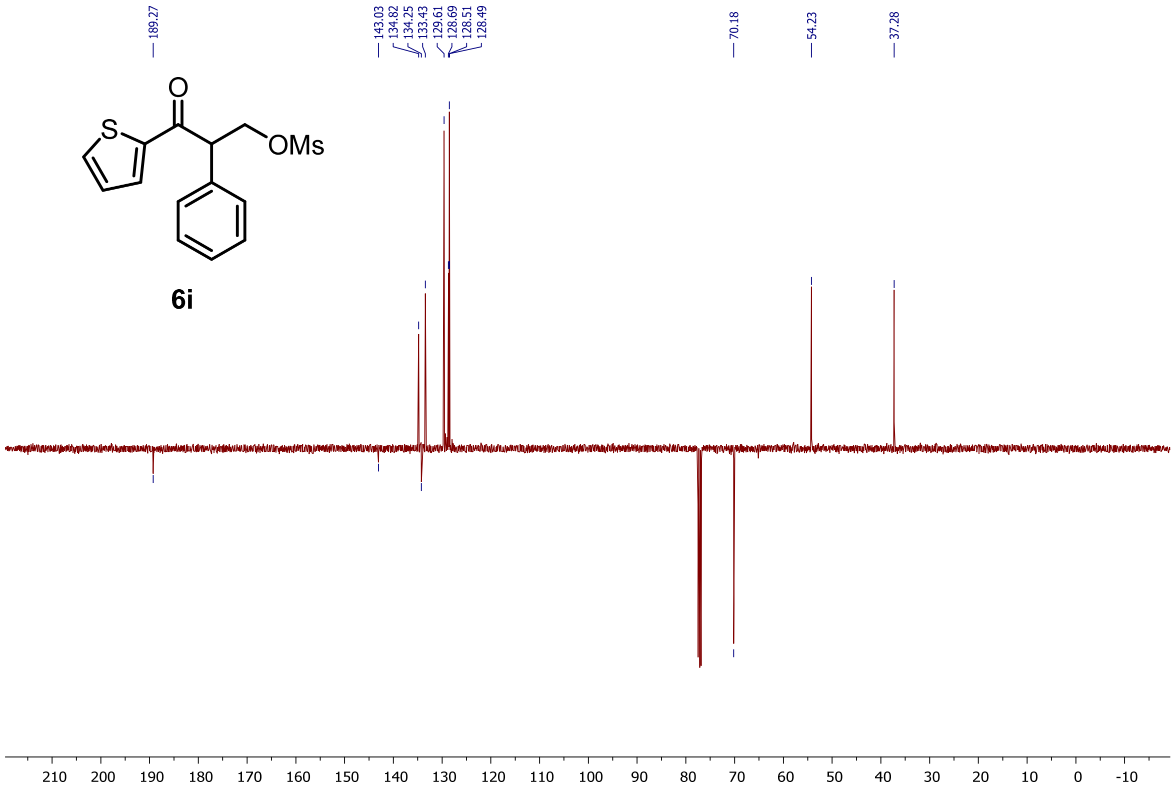

^{13}C NMR spectra of **6i**

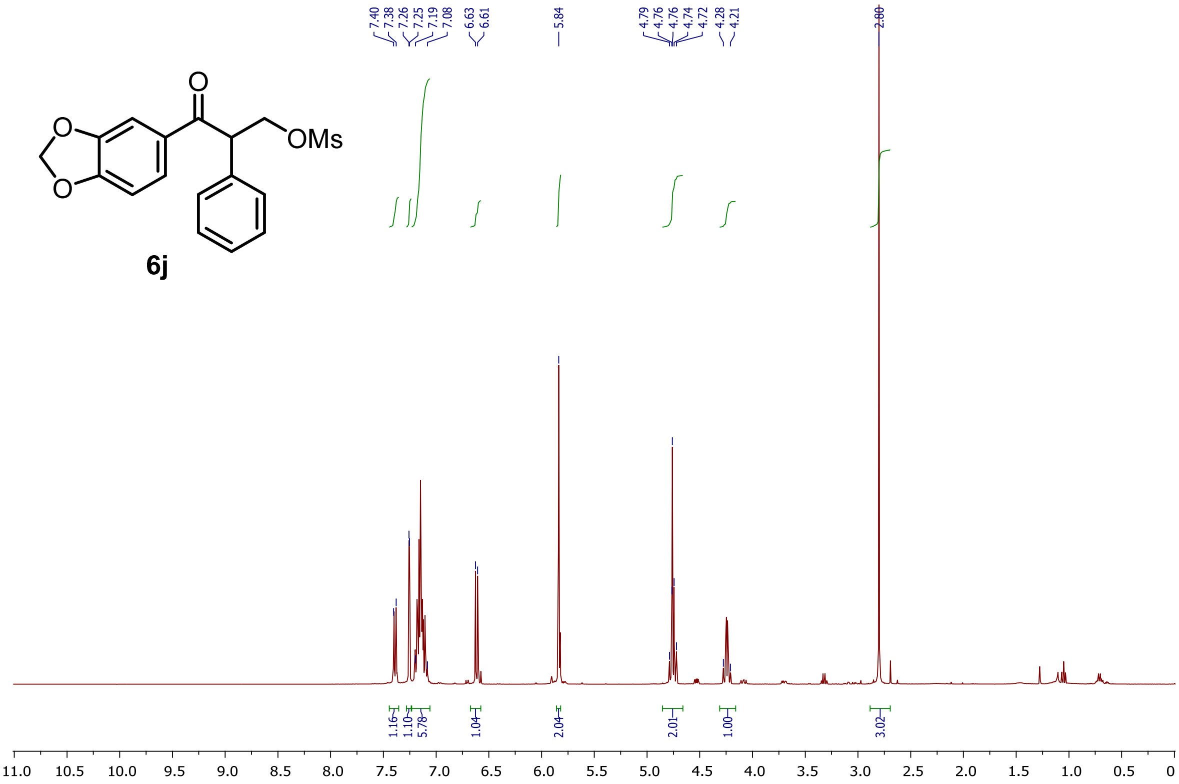

^{1}H NMR spectra of **6j**

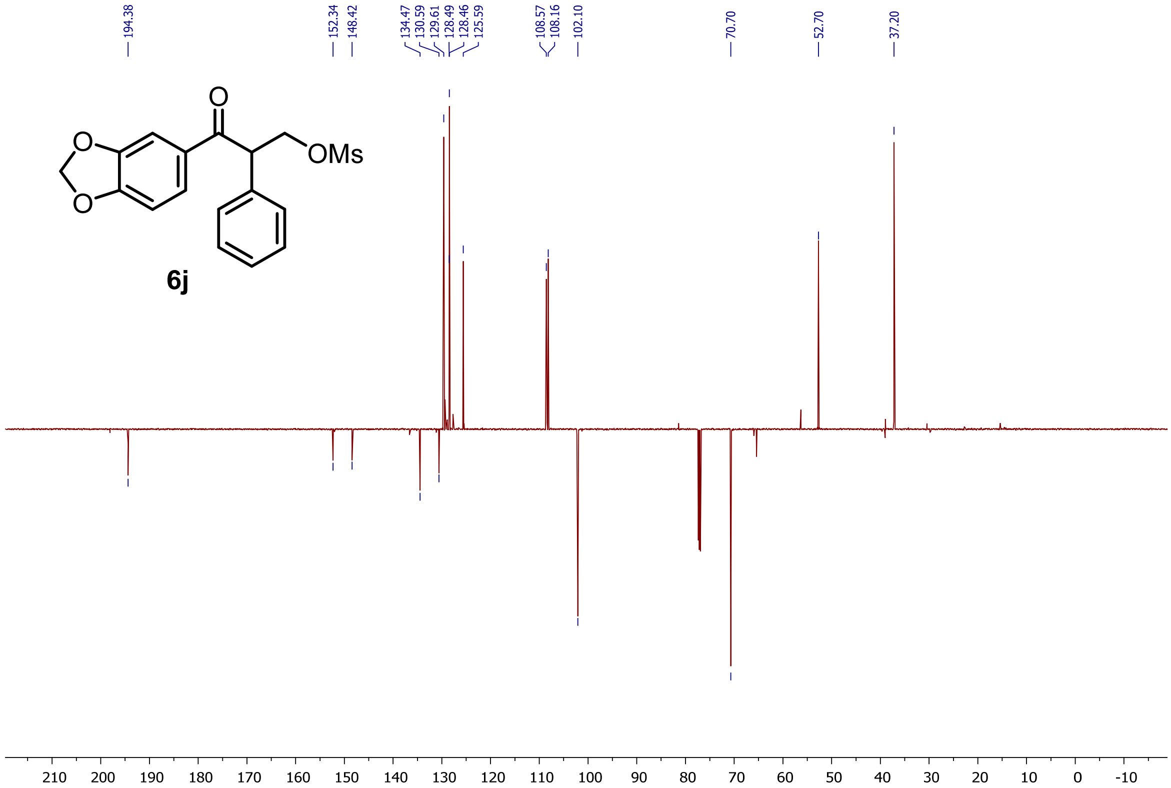

^{13}C NMR spectra of **6j**

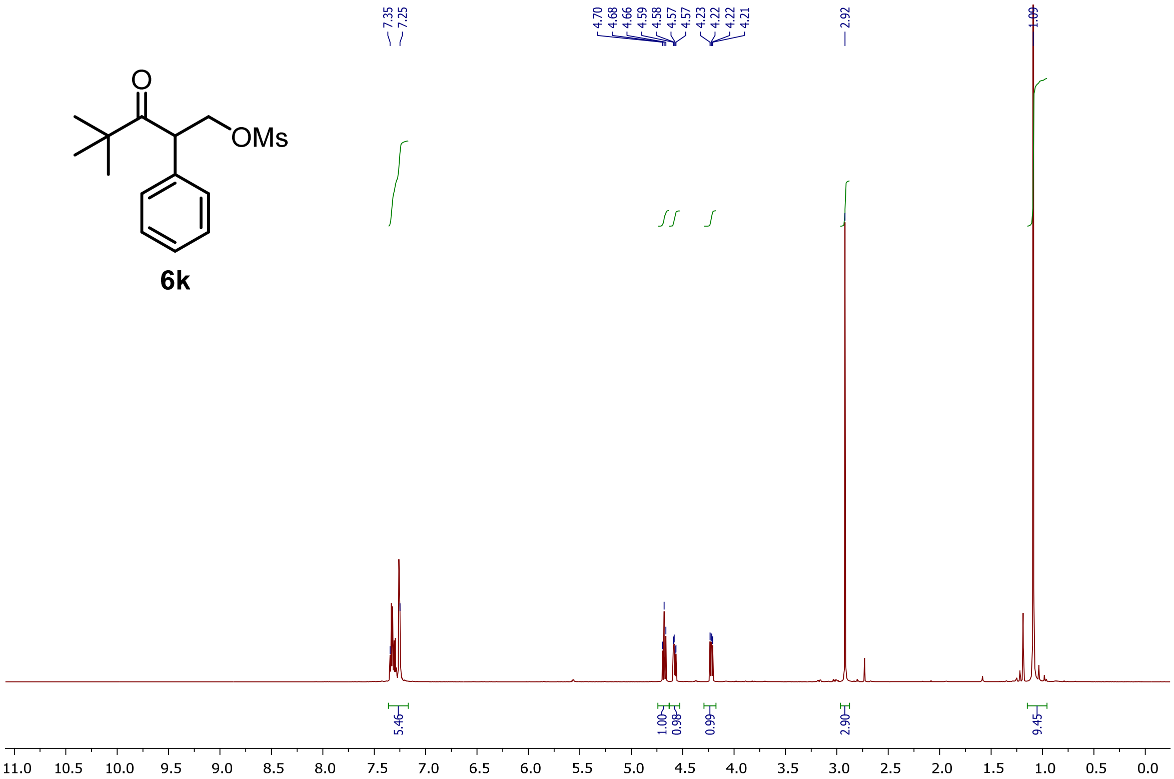

6k

^{1}H NMR spectra of **6k**

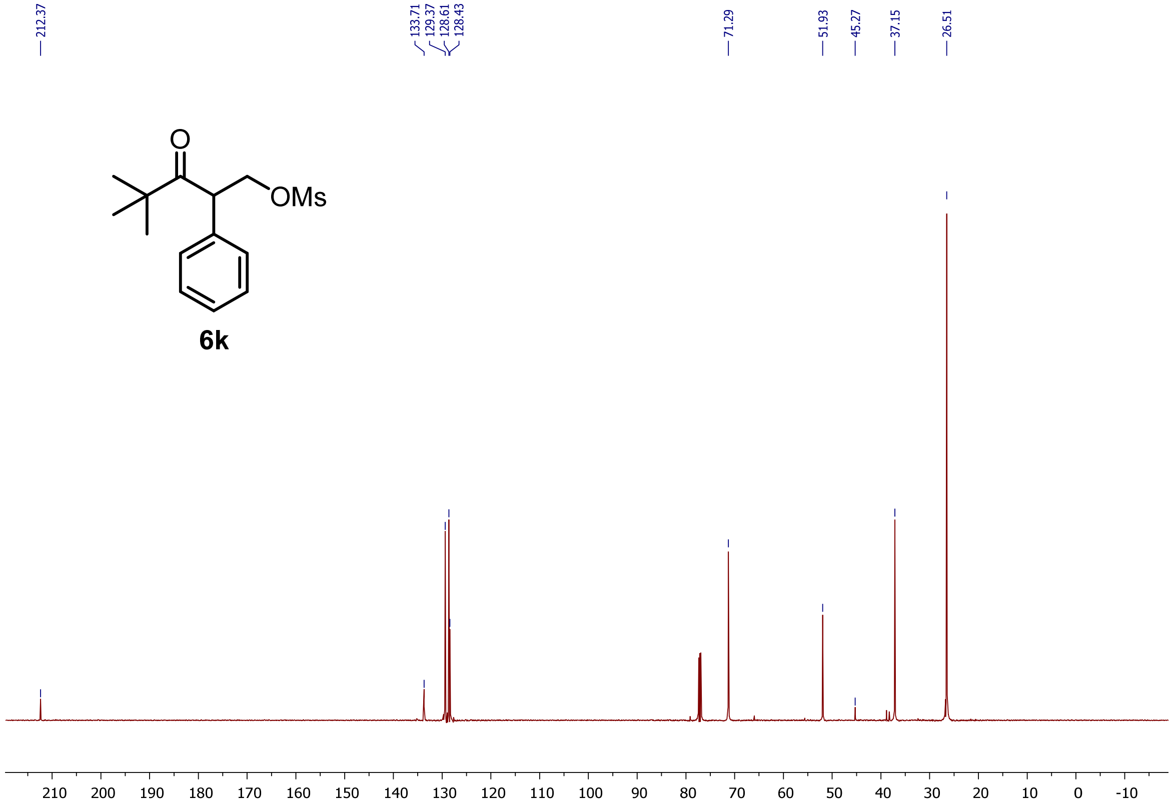

6k

^{13}C NMR spectra of **6k**

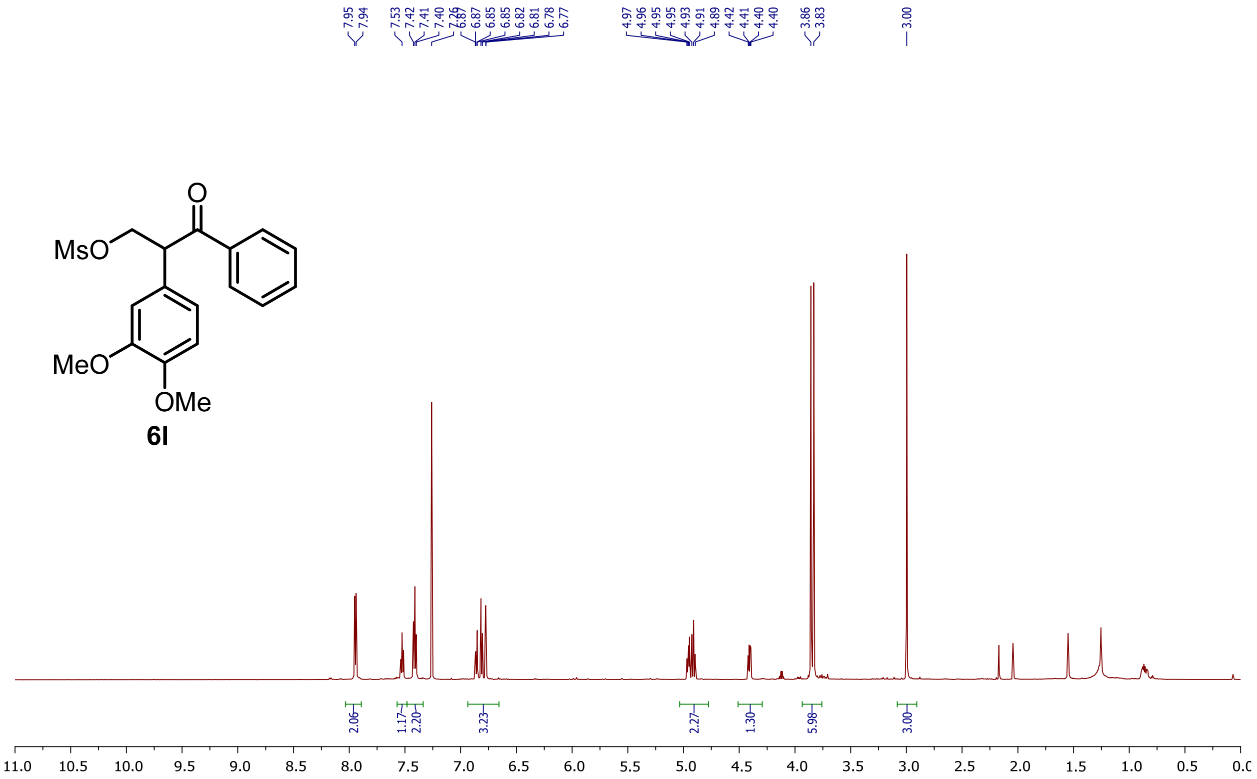

^{1}H NMR spectra of **6l**

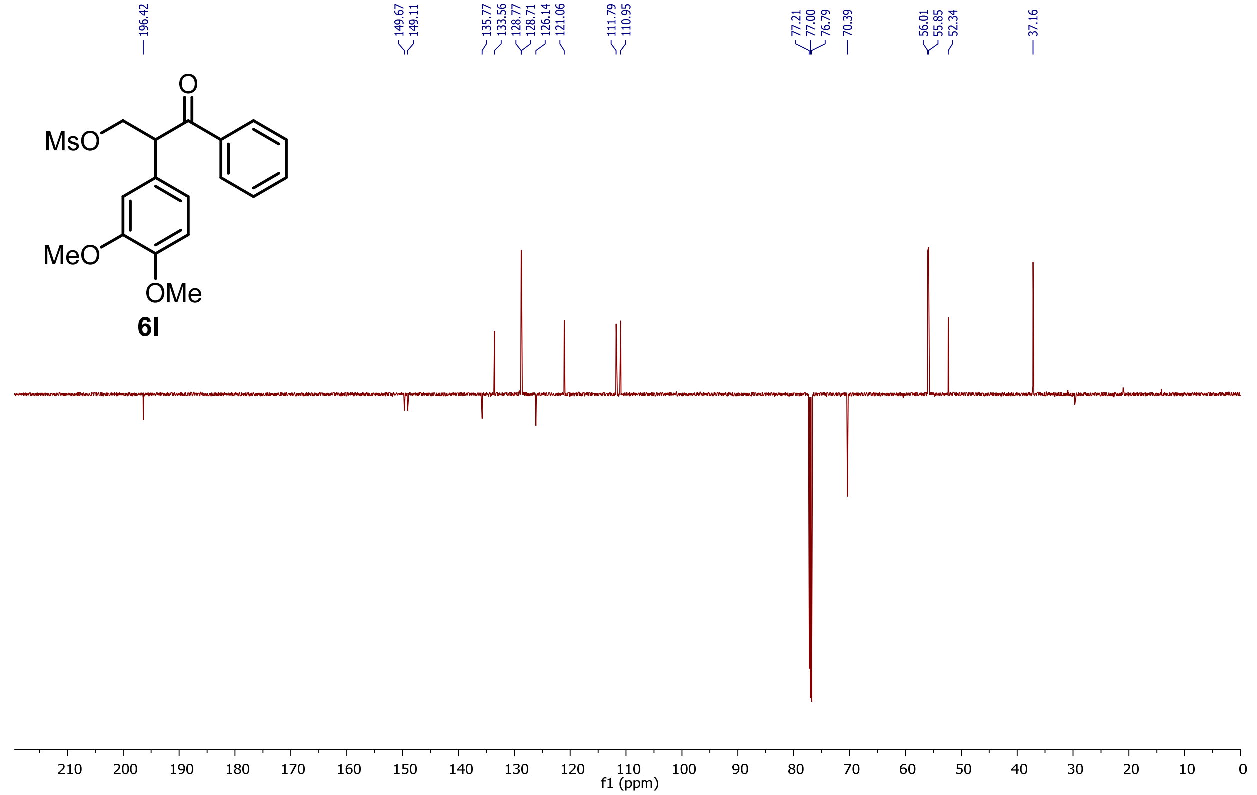

^{13}C NMR spectra of **6l**

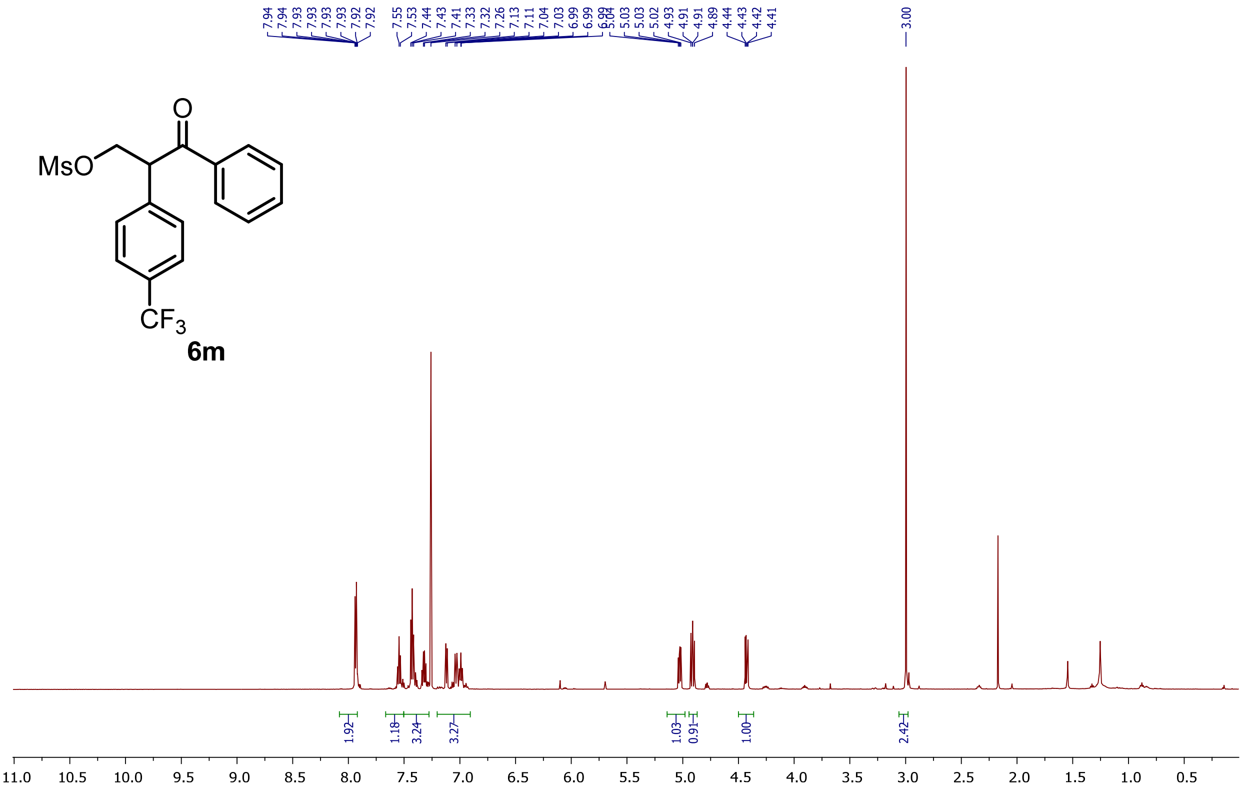

^{1}H NMR spectra of **6m**

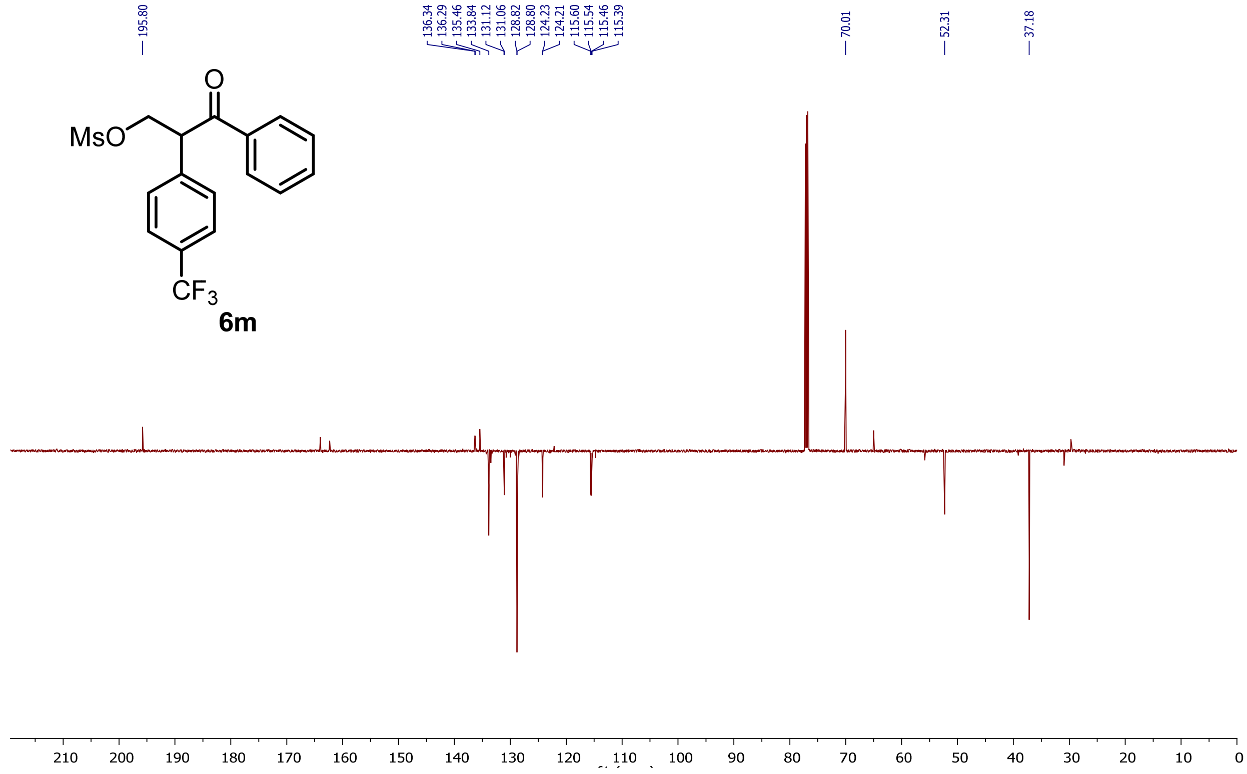

^{13}C NMR spectra of **6m**

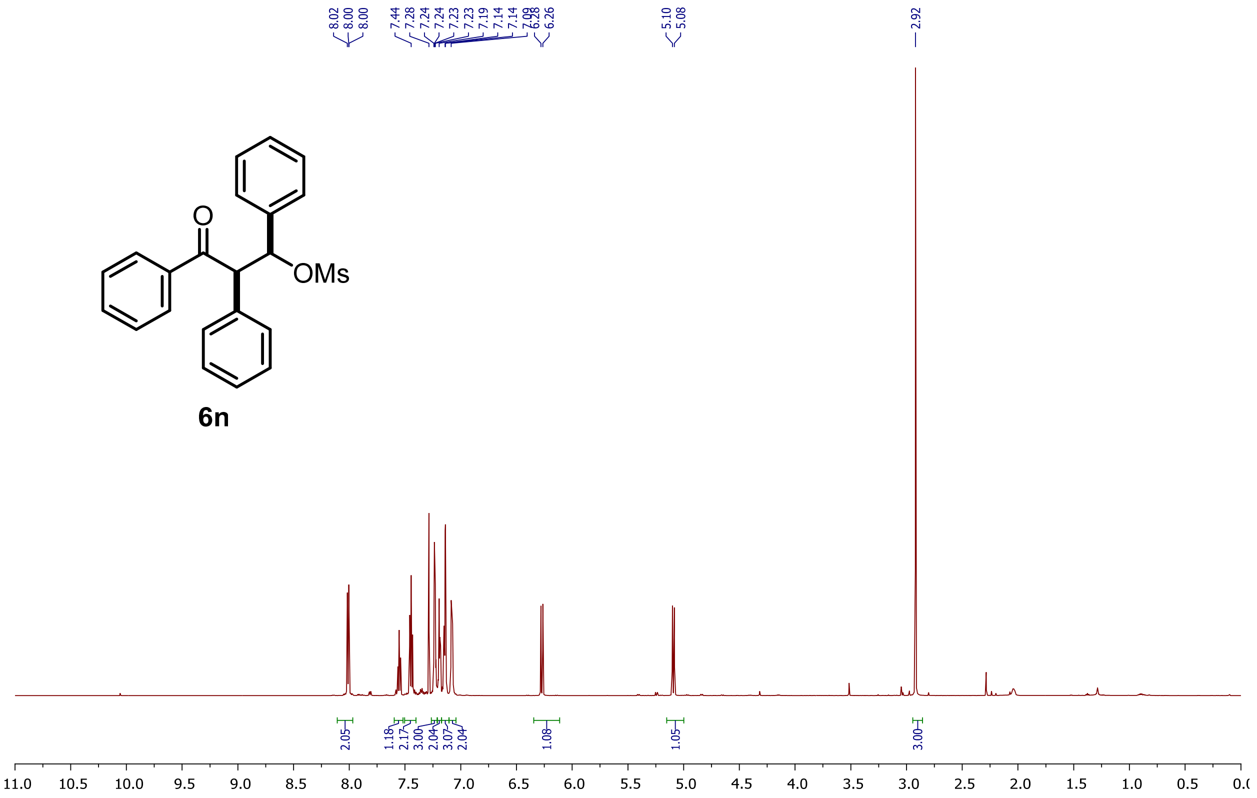

^{1}H NMR spectra of **6n**

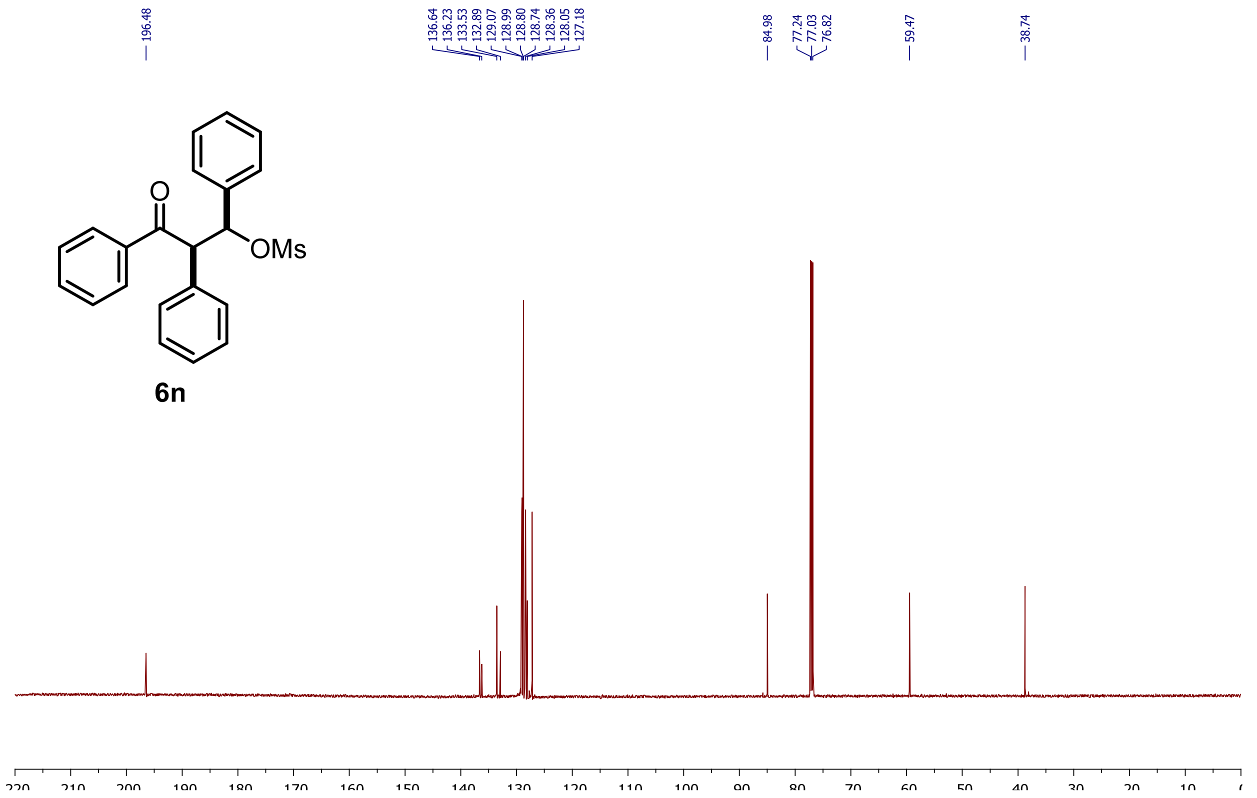

^{13}C NMR spectra of **6n**

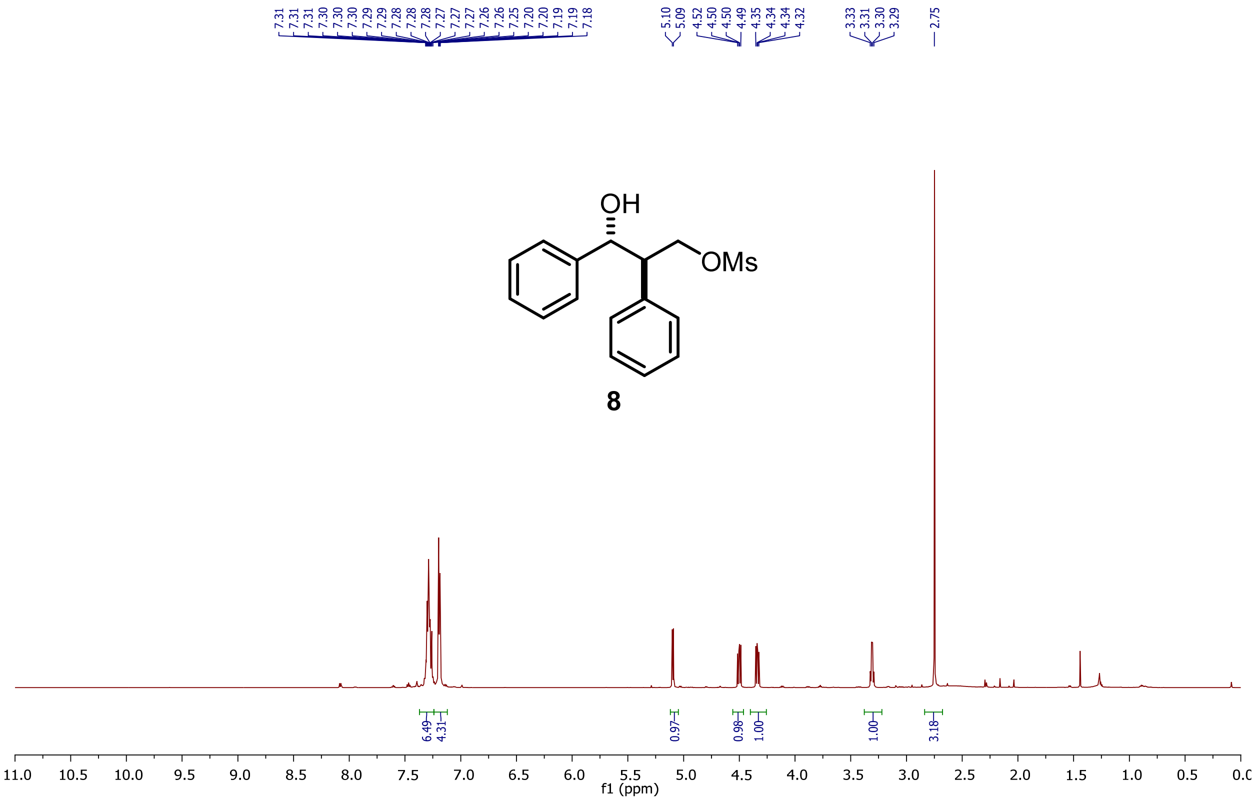

^{1}H NMR spectra of **8**

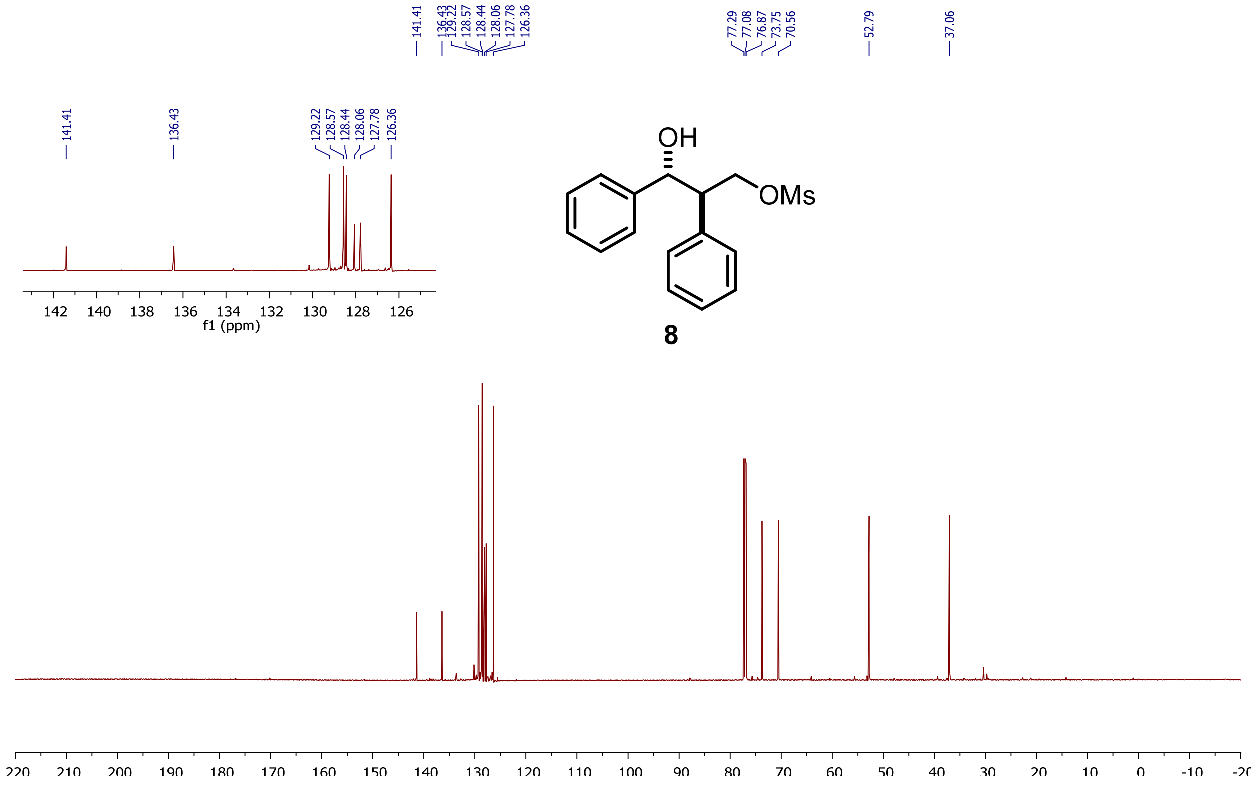

^{13}C NMR spectra of **8**

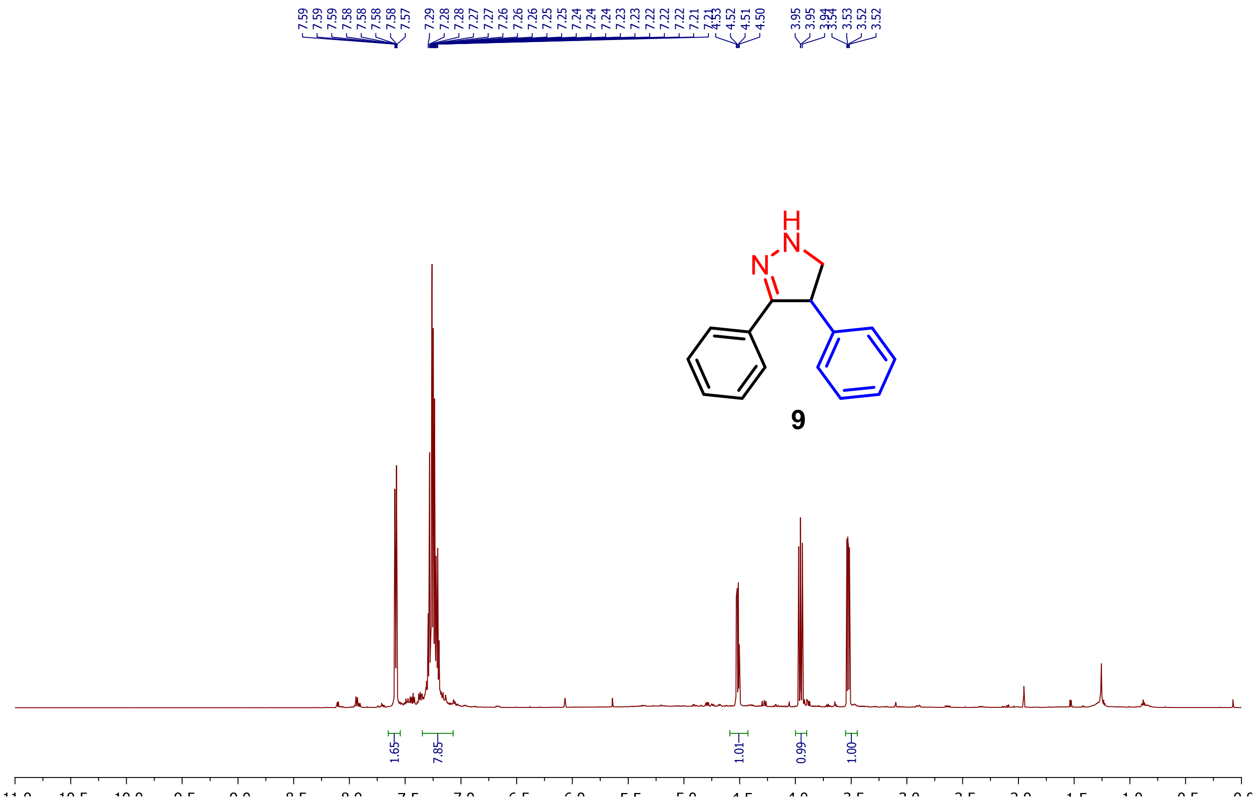

^{1}H NMR spectra of **9**

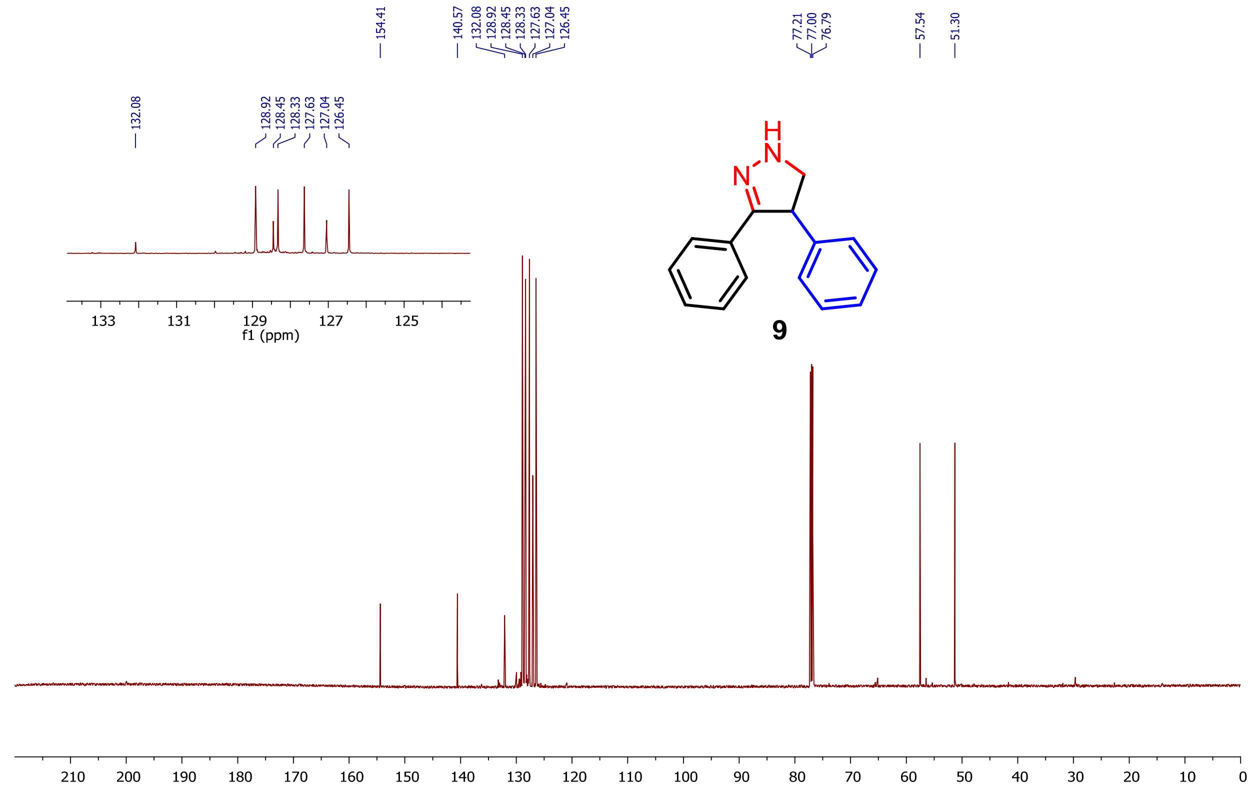

^{13}C NMR spectra of **9**

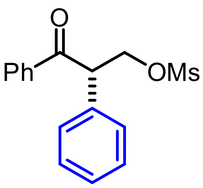

(-)-**6a**

Method Description:
Column:Chiralpak AS-3 150x4,6mm
Solvent System: n-Heptan+0,1%IPA/IPA 83:17
Flow:0,7 ml/min
T=25°C

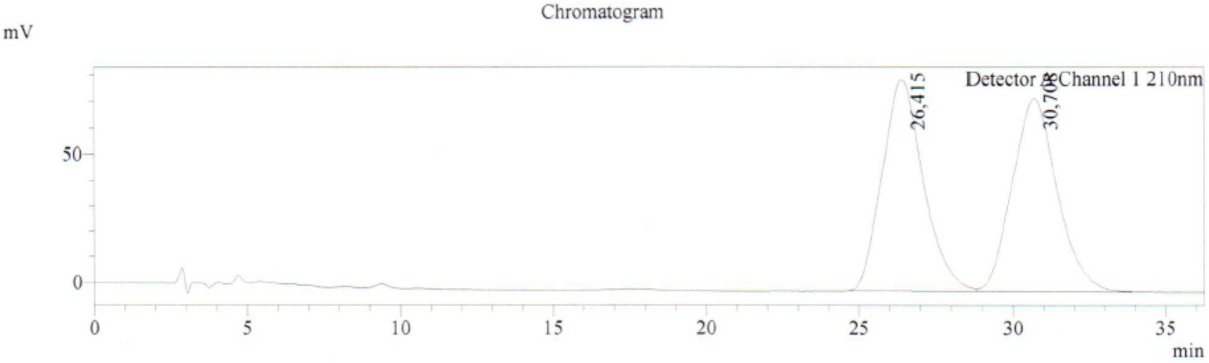

Detector A Channel 1 210nm

Peak#	Ret. Time	Area	Area%
1	26,415	7728981	50,054
2	30,708	7712252	49,946
Total		15441233	100,000

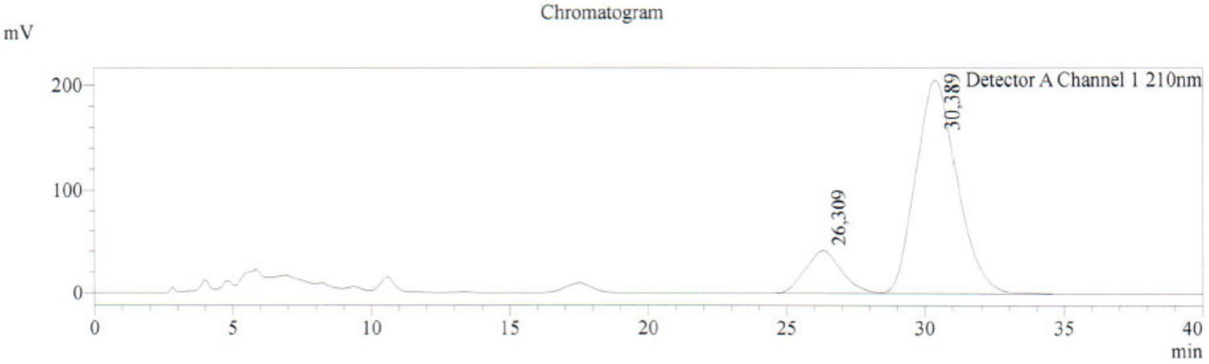

Detector A Channel 1 210nm

Peak#	Ret. Time	Area	Area%
1	26,309	3840528	15,303
2	30,389	21256676	84,697
Total		25097204	100,000
